# Effects of alcohol misuse on the evolution of anxiety during the COVID-19 pandemic in France: results from CONFINS cohort

**DOI:** 10.1136/bmjopen-2025-105567

**Published:** 2026-01-06

**Authors:** Charline Galesne, Julie Arsandaux, Mélissa Macalli, Nathalie Texier, Stéphane Schück, Christophe Tzourio, Shérazade Kinouani

**Affiliations:** 1 Bordeaux Population Health, University of Bordeaux College of Health Sciences, Bordeaux, France; 2 Laboratoire de psychologie des Pays de la Loire, Nantes University, Nantes, France; 3 Kappa Santé, Paris, France; 4 Kap Code, Paris, France; 5 Department of General Practice, University of Bordeaux College of Health Sciences, Bordeaux, France

**Keywords:** COVID-19, Anxiety disorders, Substance misuse

## Abstract

**Abstract:**

**Objectives:**

to describe the evolution of anxiety during the COVID-19 pandemic in France and to assess whether it differed according to pre-existing alcohol misuse.

**Design:**

A prospective longitudinal study.

**Setting:**

A French online cohort: CONFINS. Data has been collected since the first lockdown in April–May 2020 until January 2022.

**Participants:**

1868 participants being at least 18 years of age and who had been confined in France by government measures.

**Primary outcome measure:**

The primary outcome was anxiety, measured through *Generalised Anxiety Disorder – seven* items (GAD-7). Its association with alcohol misuse (defined using AUDIT-C score) was estimated using segmented linear mixed models. Interactions with gender and perceived loneliness at baseline were evaluated.

**Results:**

Of the 1868 included participants, 729 responded to at least one follow-up questionnaire (median follow-up time: 46 weeks). We identified 58% as having pre-existing alcohol misuse. Alcohol misuse was significantly associated with an increased GAD-7 score starting at the second lockdown in women (β=0.30; p=0.014) and in participants having a high perceived loneliness (β=0.59; p=0.011).

**Conclusions:**

Pre-existing alcohol misuse appeared to be a risk factor for anxiety during the COVID-19 pandemic, particularly for women and those with high perceived loneliness. Mental health support should be proposed to these vulnerable groups in the event of a future health crisis.

**STRENGTHS AND LIMITATIONS OF THIS STUDY**

- A longitudinal design: Data were collected during the three French lockdowns and a few months afterwards.
- A broad adjustment: tending to estimate unbiased association.
- A sensitivity analysis: to partly estimate the direction of the association between alcohol misuse and anxiety.
- A convenience sample, not representative of the French population.
- A high attrition rate during follow-up.

## Introduction

 Alcohol misuse is the term used to describe different types of alcohol consumption that may be harmful to mental or physical health because of the frequency of use, the amount consumed per occasion or the risks taken at the time of consumption. Beyond the individual health consequences, alcohol misuse has significant social repercussions, contributing to relationship difficulties, professional problems and an increase in accidents and violence.^1^ In France, the burden of alcohol use is high, with nearly 50 000 deaths attributed to alcohol consumption in 2009, representing 13% of total mortality among men and 5% among women.^2^

The bidirectional relationship between alcohol misuse and anxiety involves complex psychological, behavioural and neurobiological factors.^3^ Individuals with anxiety disorders often turn to alcohol use as a coping mechanism to reduce symptoms.^4 5^ Conversely, chronic alcohol misuse can induce or exacerbate anxiety disorders by disrupting emotional regulation and increasing stress levels, particularly during withdrawal periods.^4 5^ Research consistently shows that people with one condition are at higher risk of developing the other, emphasising the importance of addressing both issues together in treatment.^3^,^5^

During the COVID-19 pandemic, France established social distancing measures and a generalised containment strategy to curb the spread of the virus. Three lockdowns were implemented in France: the first from March 17 to May 11 of 2020; the second from October 30 to December 15 of 2020; and the third from April 3 to May 3 of 2021. Each lockdown was a distinct period, with different degrees of restrictions on the population’s movements and activities. In addition to the virus’ effects on physical health, social distancing measures and lockdowns had serious repercussions on mental health. Several studies reported an increased risk of anxiety symptoms as soon as the first lockdown was implemented.^6 7^ These symptoms were then exacerbated by the second wave of the COVID-19 pandemic.^8 9^ During the different lockdown periods, anxiety evolved differently depending on gender, with women reporting high levels of anxiety more frequently than men.^10 11^

Alcohol use is usually associated with perceived loneliness. Before the COVID-19 pandemic, an increase of perceived loneliness was found to be associated with higher levels of alcohol consumption.^12 13^ The implementation of social distancing and lockdowns for nearly 2 years exposed a large number of people to social isolation and loneliness. Studies reported an increase of perceived loneliness after the first wave of the COVID-19 pandemic, which was often associated with higher risk of depression and anxiety.^14^ Some studies have also shown a link between perceived loneliness and increased alcohol use during the COVID-19 pandemic.^15 16^

Only sparse longitudinal data are available regarding the association between declining mental health and pre-existing alcohol misuse during the COVID-19 pandemic. To understand the evolution of anxious symptoms in people suffering from alcohol misuse throughout the pandemic, it is necessary to consider personal vulnerability factors (e.g. perceived loneliness) and the different specific periods of the pandemic.

Our study is based on data from the CONFINS cohort, which examined several aspects of mental health during the COVID-19 pandemic, including anxiety, depression, stress and self-esteem. In our current analyses, we focused specifically on anxiety, investigating the relationship between pre-existing alcohol misuse and changes in anxiety in France. We considered the following hypotheses:

People with pre-existing alcohol misuse presented higher levels of anxiety symptoms during the first COVID-19 lockdown and more frequently experienced increases of these symptoms throughout the health crisis.

Being a woman and having high perceived loneliness were additional vulnerability factors for the development or increase of anxiety symptoms.

In people with low levels of anxiety during the first lockdown, pre-existing alcohol misuse facilitated the progression to higher levels of anxiety throughout the health crisis.

## Methods

This article follows the STROBE checklist for reporting cohort studies (see online supplemental files 1).

### Study design, participant recruitment and follow-up

The current analyses were performed using data from the CONFINS study, a prospective online cohort study, carried out in the adult population of France from April 2020 until January 2022. Participants were recruited through online and media channels, including social networks (LinkedIn, Twitter and Facebook) of the University of Bordeaux and the partner research organisation hosting the database, as well as several press releases and media interviews with the principal investigators. The inclusion criteria were being 18 years of age or older and having been confined in France by government measures. Participants first completed a baseline questionnaire (see online supplemental material 2) and were then asked to complete monthly follow-up questionnaires (see online supplemental material 3), with reminders sent via newsletters, emails and text messages.

We retained participants included in the cohort during the first French lockdown (between April 8 and May 11 of 2020), who had consumed alcohol in the past 12 months before inclusion and who did not have missing data regarding age, gender or their baseline anxiety level. These participants were then followed from 12 May 2020 to 13 January 2022.

### Measures

#### Main exposure: alcohol misuse

The main independent variable was the presence of alcohol misuse during the 12 months preceding inclusion in the CONFINS study, according to the Alcohol Use Disorders Identification Test-Consumption or AUDIT-C.^17 18^ The test comprises three questions and is scored on a scale of 0 to 12. Alcohol misuse was defined as a score of ≥4 for men and ≥3 for women. This score was only achievable by participants who reported drinking alcohol at least once per month during the past 12 months. People who reported drinking alcohol less than once per month were categorised as not having alcohol misuse.

#### Main dependent variable: level of anxiety

The level of anxiety was determined at baseline and during follow-up. Anxiety level was measured using the *Generalised Anxiety Disorder – seven* items (GAD-7).^19 20^ The anxiety level was treated as a quantitative variable. The score ranged from 0 to 21, with a higher score indicating more anxiety symptoms. For descriptive purposes, we also used the following categorisation: minimal (0–4), mild (5–9), moderate (10–14) and severe (15–21).

#### Covariates

We identified potential confounders at baseline that could be associated with the presence of alcohol misuse and mental health outcome, based on prior literature and theoretical considerations regarding factors likely to influence both exposure and outcome: age (<25 or ≥25 years), gender (man or woman), marital status (single, in a relationship/married, or divorced/widowed), education level (<2 years, 2 years, or >2 years post-high school diploma), reporting any medical diagnosis of a mental health disorder in one’s lifetime (yes, no, or don't know), tobacco smoking in the last 12 months (smoker, ex-smoker, or non-smoker) and perceived loneliness (low or high). Perceived loneliness was self-assessed with the question: “In the last 7 days, on a scale of 0–10 (0=not at all, 10=totally), how lonely do you feel?”. High perceived loneliness was defined as a loneliness score of ≥7, which corresponded to the variable’s upper quartile.

### Statistical analyses

First, descriptive statistics of the baseline characteristics were computed for the total sample and then stratified according to alcohol misuse status. We also compared participants without follow-up to those who answered at least one follow-up questionnaire. Categorical variables were described with frequencies and percentages, and continuous variables with median and IQR. Mean scores and SD were calculated to schematise the evolution of the outcome, measured at each period of the COVID-19 pandemic in France (see online supplemental table 1), and stratified by alcohol misuse, gender and perceived loneliness.

Second, we estimated the effect of pre-existing alcohol misuse on anxiety at baseline during the first lockdown (intercept) and on anxiety change (slope with GAD-7 score/trimester) using segmented linear mixed-effect models. We chose to model the evolution of the GAD-7 score using two segments connected at a single cut-off point positioned at the week corresponding to the beginning of the second lockdown (defined in CONFINS as the week of 1–7 November 2020). To determine the position of this cut-off point, we plotted the individual curves and an average curve of the evolution of GAD-7 scores. From this, we noticed that the GAD-7 scores seemed to evolve differently between the inclusion period until the end of the first deconfinement versus during the period from the second lockdown until the end of follow-up.

To study the fixed effect of the presence of alcohol misuse on the evolution of anxiety, we incorporated this variable into our models by including a two-interaction term between this variable and the slope of each segment. Therefore, we chose to add a random slope only to the second segment (eg, the slope representing the time after the second lockdown) since we expected to find greater inter-individual variability for this more distant effect. To handle the correlation between the intercept and the random slope, we used a covariance matrix of random effects fixed at 0. The parameters of our final models were estimated using maximum likelihood, which is adequate for large samples. The segmented mixed linear models were adjusted for parameters collected at baseline: gender, age, perceived loneliness, history of mental health disorders, smoking status, level of education and marital status. We tested interactions between gender or perceived loneliness and alcohol misuse, which were pre-specified based on prior literature suggesting possible differences. Stratified models were adjusted for the same covariates described above.

We performed a sensitivity analysis, repeating the segmented linear mixed-effect models only in participants with minimal-to-mild anxiety at baseline (GAD-7<10). These models explored the role of alcohol misuse in the onset and evolution of anxiety among participants with few symptoms at baseline (incident cases).

All segmented linear mixed effect models were presented with their coefficient β and 95% CI. Statistical significance was defined as a p value of<0.05. All analyses were performed using R 4.3.0.

## Results

### Participant and follow-up characteristics

**Figure 1:**
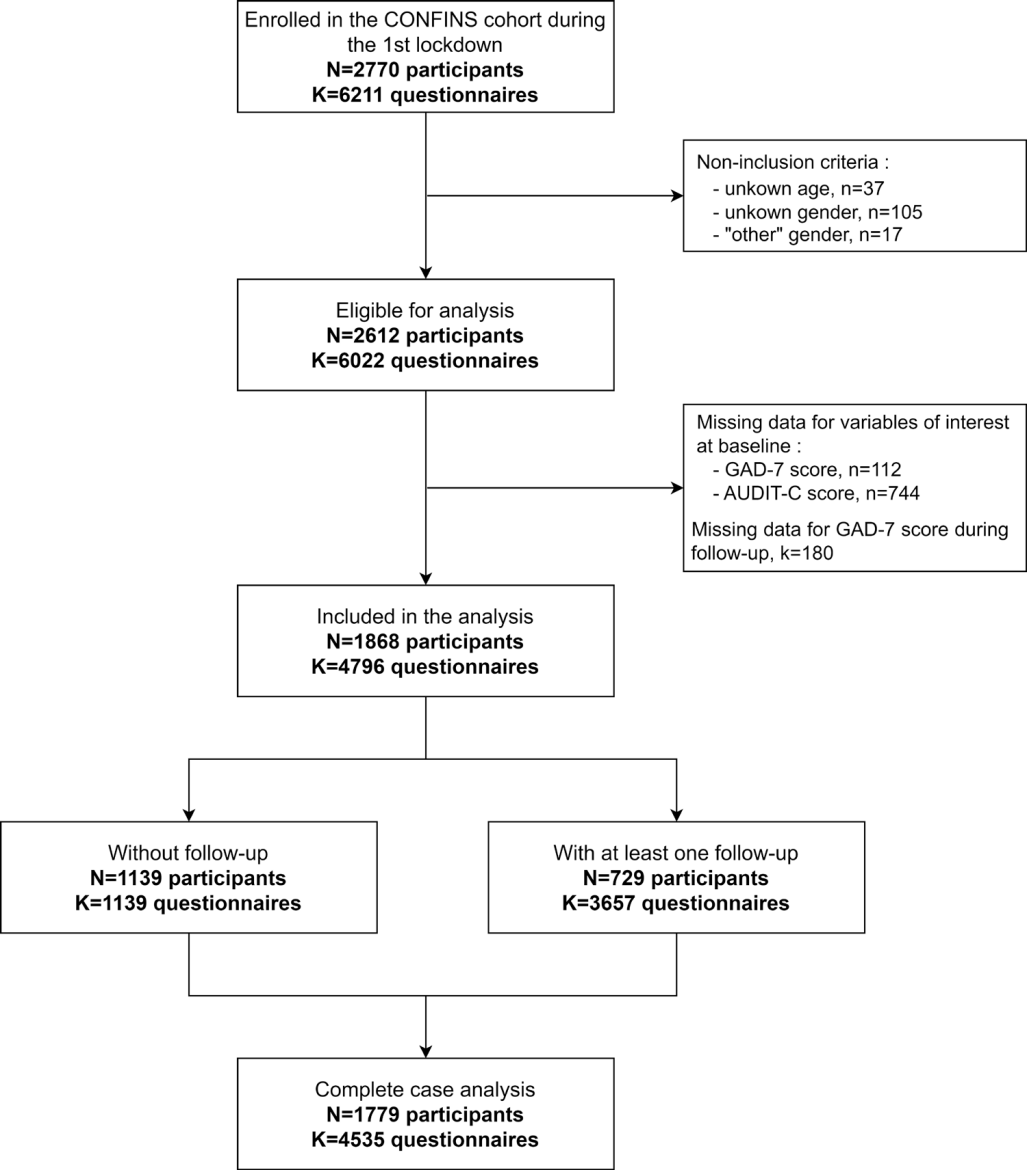
Flow chart for sample selection. CONFINS cohort, France, 2022. GAD-7, Generalised Anxiety Disorder – seven items.

During the first lockdown in France, 2770 subjects were included in the CONFINS cohort (figure 1). These participants completed a total of 6211 questionnaires (counting both inclusion and follow-up questionnaires). Our analyses included the participants who consumed alcohol and did not have missing data regarding their age, gender, AUDIT-C score or anxiety level. The final sample comprised 1868 participants. Due to additional missing data on covariates, the analytical sample for the mixed-effect models consisted of 1779 participants. Within this sample, 61% of participants completed only the inclusion questionnaire, and 39% completed at least one follow-up questionnaire. We compared the characteristics of the participants without follow-up versus those who answered at least one follow-up questionnaire: both groups included a similar proportion of participants with alcohol misuse (see online supplemental table 2). On the other hand, the group without follow-up included more participants under 25 years, current smokers and participants with moderate-to-severe anxiety.

The participants with at least one follow-up were followed for a median duration of 46 weeks, ranging from 8 to 92 weeks. The number of follow-up questionnaires answered and the length of follow-up did not differ between participants without alcohol misuse and those with alcohol misuse.

#### Description of the sample at baseline

Table 1 presents participants’ characteristics. In the total sample, 78.8% were women. Over half of the participants were more than 25 years old (56.9%), in a relationship or married (57.3%) and had completed more than 2 years of education post-high school diploma (66%). Within the whole population, 23.8% were current smokers, 10.7% were ex-smokers and 65.5% were non-smokers. Less than a quarter of the participants reported high perceived loneliness (22.4%) and having a history of mental health disorders (21.8%). Anxiety symptoms at baseline were mostly minimal (53.4%), while 24.1% reported mild, 13.2% moderate and 9.2% severe symptoms.

**Table 1:** Participants’ characteristics at baseline. CONFINS cohort, France, 2022.

Characteristics	General population n=1868		Without alcohol misuse n=**775**		With alcohol misuse n=**1093**	
	N	%	N	%	N	%
Gender						
Man	396	21.2	188	24.3	208	19.0
Woman	1472	78.8	587	75.7	885	81.0
Age						
<25 years	806	43.1	330	42.6	476	43.5
≥25 years	1062	56.9	445	57.4	617	56.5
Marital status						
Single	752	40.3	295	38.1	457	41.8
In a relationship or married	1071	57.3	455	58.7	616	56.4
Divorced or widowed	45	2.4	25	3.2	20	1.8
Years of education post-high school diploma						
<2 years	275	14.7	142	18.3	133	12.2
2 years	279	14.9	129	16.6	150	13.7
>2 years	1232	66.0	471	60.8	761	69.6
Missing data	82	4.4	33	4.3	49	4.5
History of mental health disorders						
Yes	408	21.8	164	21.2	244	22.3
No	1415	75.7	597	77.0	818	74.8
Don’t know	45	2.4	14	1.8	31	2.8
GAD-7 score, median(IQR†)	4(1–9)		4(1–9)		4(2–9)	
Anxiety level						
Minimal	998	53.4	421	54.3	577	52.8
Mild	451	24.1	174	22.5	277	25.3
Moderate	247	13.2	109	14.1	138	12.6
Severe	172	9.2	71	9.2	101	9.2
AUDIT-C score, median(IQR†)	4(3–5)		2(2–2)		4(3–5)	
Alcohol consumption in the past year						
Never	0	0.0	0	0.0	0	0.0
Less than monthly	326	17.5	326	42.1	0	0.0
Monthly	244	13.1	175	22.6	69	6.3
2–4 times per month	776	41.5	263	33.9	513	46.9
2–3 times per week	410	21.9	11	1.4	399	36.5
≥4 times per week	112	6.0	0	0.0	112	10.2
Number of standard drinks on a typical day when drinking in the past year						
1–2	1009	54.0	425	54.8	584	53.4
3–4	375	20.1	24	3.1	351	32.1
5–6	124	6.6	0	0.0	124	11.3
7–9	27	1.4	0	0.0	27	2.5
≥10	7	0.4	0	0.0	7	0.6
Missing data*	326	17.5	326	42.1	0	0.0
Consumption of at least six standard drinks on one occasion in the past year						
Never	455	24.4	339	43.7	116	10.6
Less than monthly	659	35.3	109	14.1	550	50.3
Monthly	317	17.0	1	0.1	316	28.9
Weekly	105	5.6	0	0.0	105	9.6
Daily or almost daily	6	0.3	0	0.0	6	0.5
Missing data*	326	17.5	326	42.1	0	0.0
Smoking status						
Smoker	445	23.8	89	11.5	356	32.6
Ex-smoker	200	10.7	67	8.6	133	12.2
Non-smoker	1223	65.5	619	79.9	604	55.3
Perceived loneliness						
High	418	22.4	164	21.2	254	23.2
Low	1443	77.2	610	78.7	833	76.2
Missing data	7	0.4	1	0.1	6	0.5

* Missing data for participants consuming alcohol less than once a month.

† IQR=IQR range.

GAD-7, Generalised Anxiety Disorder – seven items.

Pre-existing alcohol misuse was described by 58.5% of participants. There were more men in the alcohol misuse group (24%) compared with those without alcohol misuse (19%). Drinkers with alcohol misuse also had a higher level of education, and a greater proportion of them were smokers. Participants with alcohol misuse did not seem to differ from those without alcohol misuse in terms of age, marital status, history of mental health disorders, perceived loneliness or anxiety levels (table 1).

### Effect of alcohol misuse on anxiety throughout the COVID-19 pandemic

**Figure 2:**
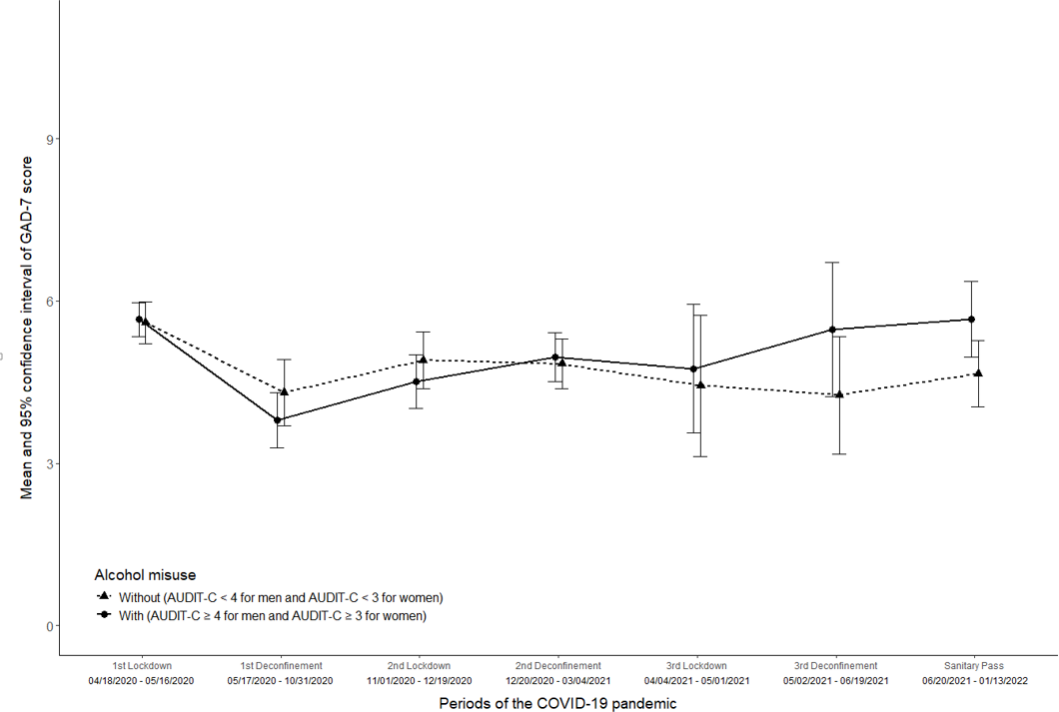
Means and SD of GAD-7 score through the different periods of the pandemic, stratified by alcohol misuse. CONFINS cohort, France, 2022. GAD-7, Generalised Anxiety Disorder – seven items.

Figure 2 presents the mean scores and SD of the GAD-7 score within the total sample according to alcohol misuse. The GAD-7 score followed the same trend in both groups between the first and second lockdown, with a decrease from baseline. However, in later periods, a slight increase in GAD-7 scores was observed among participants with alcohol misuse, whereas scores remained more stable among those without alcohol misuse.

The mixed-effect models showed that the effect of alcohol misuse on the evolution of the GAD-7 score at baseline, before and after the second lockdown, did not significantly differ based on gender and perceived loneliness. Despite these non-significant interactions, a significant association between alcohol misuse and anxiety evolution was observed among women and those with high perceived loneliness in stratified analyses. We chose to present our results stratified by gender and perceived loneliness in tables2  3 and online supplemental figures 1,2).

**Table 2:** Evolution of GAD-7 score stratified by gender, depending on the presence of alcohol misuse since the first lockdown (n=1779). CONFINS cohort, France, 2022.

	Women (n=1401)								
	GAD-7 score at baseline			Change in GAD-7 score (point/trimester) until the end of the first deconfinement			Change in GAD-7 score (point/trimester) starting at the second lockdown		
	β	95% CI	P value	β	95% CI	P value	β	95% CI	P value
Intercept/slope									
Without alcohol misuse	5.83	(5.11 to 6.55)	<0.001***	0.16	(−0.07 to 0.39)	0.181	0.01	(−0.16 to 0.18)	0.933
With alcohol misuse	5.62	(4.88 to 6.35)	<0.001***	−0.02	(−0.23 to 0.18)	0.824	0.31	(0.14 to 0.47)	<0.001***
Difference									
Without *vs* With alcohol misuse	−0.21	(−0.72 to 0.30)	0.420	−0.18	(−0.49 to 0.13)	0.250	0.30	(0.06 to 0.53)	0.014**
	**Men (n=378**)								
	**GAD-7 score at baseline**			**Change in GAD-7 score (point/trimester) until the end of the first deconfinement**			**Change in GAD-7 score (point/trimester) starting at the second lockdown**		
	**β**	**95% CI**	**P value**	**β**	**95% CI**	**P value**	**β**	**95% CI**	**P value**
Intercept/slope									
Without alcohol misuse	4.67	(3.76 to 5.58)	<0.001***	−0.18	(−0.59 to 0.24)	0.409	0.03	(−0.27 to 0.33)	0.841
With alcohol misuse	4.30	(3.40 to 5.19)	<0.001***	−0.13	(−0.54 to 0.28)	0.528	−0.01	(−0.30 to 0.29)	0.962
Difference									
Without *vs* With alcohol misuse	−0.37	(−1.31 to 0.57)	0.436	0.04	(−0.54 to 0.63)	0.884	−0.04	(−0.46 to 0.38)	0.860

Models adjusted for age, perceived loneliness, mental health disorder history, smoking status, level of education and marital status. ***p<0.01, **p<0.05, *p<0.10.

GAD-7, Generalised Anxiety Disorder – seven items.

**Table 3:** Evolution of GAD-7 score stratified by perceived loneliness, depending on the presence of alcohol misuse since the first lockdown (n=1779). CONFINS cohort, France, 2022

	High perceived loneliness (n=385)								
	GAD-7 score at baseline			Change in GAD-7 score (point/trimester) until the end of the first deconfinement			Change in GAD-7 score (point/trimester) starting at the 2nd lockdown		
	β	95% CI	P value	β	95% CI	P value	β	95% CI	P value
Intercept/slope									
Without alcohol misuse	7.78	(6.82 to 8.74)	<0.001***	−0.09	(−0.56 to 0.38)	0.703	−0.18	(−0.52 to 0.16)	0.297
With alcohol misuse	7.81	(6.80 to 8.73)	<0.001***	−0.42	(−0.83 to −0.02)	0.041**	0.4	(0.11 to 0.70)	0.008***
Difference									
Without *vs* With alcohol misuse	0.03	(−0.90 to 0.97)	0.942	−0.33	(−0.95 to 0.29)	0.296	0.59	(0.13 to 1.04)	0.011**
	**Low perceived loneliness (n=1384)**								
	**GAD-7 score at baseline**			**Change in GAD-7 score (point/trimester) until the end of the first deconfinement**			**Change in GAD-7 score (point/trimester) starting at the second lockdown**		
	**β**	**95% CI**	**P value**	**β**	**95% CI**	**P value**	**β**	**95% CI**	**P value**
Intercept/slope									
Without alcohol misuse	4.49	(3.70 to 5.27)	<0.001***	0.12	(−0.11 to 0.34)	0.301	0.06	(−0.10 to 0.23)	0.461
With alcohol misuse	4.15	(3.34 to 4.96)	<0.001***	0.06	(−0.15 to 0.27)	0.556	0.18	(0.01 to 0.34)	0.036**
Difference									
Without *vs* with alcohol misuse	−0.34	(−0.84 to 0.17)	0.193	−0.06	(−0.36 to 0.25)	0.717	0.12	(−0.12 to 0.35)	0.329

Models adjusted for age, gender, mental health disorder history, smoking status, level of education, and marital status. ***p<0.01, **p<0.05, *p<0.10.

GAD-7, Generalised Anxiety Disorder – seven items .

Table 2 shows our results stratified by gender. Alcohol misuse was not significantly associated with either the GAD-7 score at baseline and its evolution until the end of the first deconfinement for men and women. Among women, alcohol misuse was associated with a significant increase of 0.31 point/trimester of GAD-7 score starting at the second lockdown (p<0.001), while there was no change in women without alcohol misuse (p=0.933). The difference in anxiety evolution between women with and without alcohol misuse during this period was significant (β=0.30; p=0.014). In contrast, there was no association between alcohol misuse and anxiety after the second lockdown for men.

Table 3 shows our results stratified by perceived loneliness. Alcohol misuse was not significantly associated with GAD-7 score at baseline for participants with low or high perceived loneliness. Among participants with high perceived loneliness, alcohol misuse was then associated with a significant decrease of 0.42 point/trimester of GAD-7 score until the end of the first deconfinement (p=0.041), but this change was not significantly different from those without alcohol misuse (p=0.296). For both high and low perceived loneliness participants, alcohol misuse was associated with a significant increase of 0.40 and 0.18 point/trimester, respectively, from the second lockdown (p=0.008 and p=0.036). The difference in anxiety evolution between participants with and without alcohol misuse during this period was only significant among those with high perceived loneliness (β=0.59; p=0.011).

### Sensitivity analysis

The results are shown in online supplemental tables 3,4. Considering only participants who had minimal-to-mild anxiety at baseline, the effect of alcohol misuse on the evolution of the GAD-7 score at baseline, before and after the second lockdown, did not significantly differ based on gender, but was significantly associated with perceived loneliness from the second lockdown (p of interaction=0.045). In all subgroups, there was no significant difference in GAD-7 score between drinkers with and without alcohol misuse at baseline and before the second lockdown. However, the presence of alcohol misuse tended to increase the GAD-7 score by 0.30 point/trimester starting at the second lockdown among participants with high perceived loneliness (p=0.083), and significantly increased the GAD-7 score by 0.30 point/trimester starting at the second lockdown among women (p=0.001). The difference in anxiety evolution between drinkers with and without alcohol misuse was significant in participants with high perceived loneliness (β=0.64; p=0.016) and almost significant in women (β=0.24; p=0.059).

## Discussion

### Findings of the study

In this prospective study, we first found that the effect of alcohol misuse on the evolution of anxiety during the COVID-19 pandemic between March 2020 and January 2022 did not significantly globally differ based on gender and perceived loneliness. However, when we examined the results stratified by these factors, we found that anxiety increased significantly starting at the second lockdown in women and individuals with high perceived loneliness when alcohol misuse was present, compared with those without alcohol misuse. This suggests that being a woman and experiencing loneliness are vulnerability factors in the relationship between alcohol misuse and anxiety. Our sensitivity analysis in incident cases yielded similar findings: the same trend of increased anxiety from the second lockdown was observed among women or individuals with high perceived loneliness in the presence of alcohol misuse.

A few longitudinal studies have examined the evolution of anxiety throughout the COVID-19 health crisis. The initial shock of the pandemic generally increased people’s anxiety levels.^6 7^ One study led at the beginning of the pandemic reported an association between anxiety and a history of alcohol use disorder.^21^ In one student population, anxiety or depression symptoms worsened with the pandemic, especially during Fall 2020, with increased depression being linked to higher levels of pre-pandemic alcohol use.^22^ These results corroborate our current findings, as does previous research not related to COVID-19.^23^

During the COVID-19 pandemic, women were consistently identified as being at greater risk of high anxiety and depressive symptoms than men in the general population.^24^,^26^ Biological vulnerabilities, combined with socialisation processes and gender role expectations, have long been recognised as contributing to women’s higher susceptibility to anxiety.^27^,^29^ Moreover, among individuals with substance use disorders, women also appear more vulnerable to anxiety and depression, partly due to higher levels of neuroticism and greater reliance on emotion-focused coping strategies.^30 31^ The pandemic context may have further exacerbated these gender differences. Women were disproportionately affected by the social and economic consequences of lockdowns, such as increased childcare responsibilities, work interruptions and job losses.^32^ In addition, women are overrepresented in healthcare and caregiving professions, sectors that faced particularly high levels of stress and emotional burden during the pandemic.^33^,^37^ These combined stressors may explain the higher anxiety levels observed among women during the pandemic, as also highlighted by studies in perinatal populations.^38^

Perceived loneliness was already recognised as a risk factor for severe mental health disorders.^39^ Among people with substance use problems, loneliness is also consistently linked to poor mental health.^40^ During the pandemic, greater perceived loneliness was reported to be significantly associated with higher levels of anxiety,^14^ which in turn was associated with harmful levels of alcohol use.^41^ Studies focusing on perceived social support found it to be a protective factor for mental health early in the pandemic.^42^ We might assume that in the context of the COVID-19 crisis, strong perceived social support was an important resource for people with alcohol misuse. In line with this, a study examining the associations between alcohol consumption and mental health during the pandemic found that loneliness mediated the relationship between alcohol use and anxiety or depressive symptoms, highlighting its central role in this context.^43^

### Strengths and limitations

Our current study had several limitations. First, the participants in this study were recruited through convenience and snowball sampling using online networks and press communications and, therefore, cannot be representative of the French population at large. This recruitment strategy, while allowing for rapid data collection, may have introduced self-selection bias, as individuals who are more active on social media or more interested in health-related topics were more likely to participate. Consequently, there is an overrepresentation of certain sociodemographic categories, notably women and young adults. Such biases, inherent in studies based on online cohorts and snowball non-probability sampling, may limit the generalisability of our results to the entire population. Similar limitations have been reported in studies using online social media recruitment strategies.^44 45^ Because women generally report lower levels of alcohol misuse but higher levels of anxiety than men, their overrepresentation may have led to an underestimation of the overall prevalence of alcohol misuse and an overestimation of anxiety symptoms in our sample. Nevertheless, our analyses were adjusted for age and stratified for gender, which should have limited the potential impact of this bias on the observed associations. Furthermore, the overrepresentation of younger and more educated individuals, who may have experienced the pandemic differently from older or less connected groups, could also affect the generalisability of our results. Second, the participants self-reported their pre-pandemic and current information, which may have led to recall or social desirability bias and biased declaration of perceived loneliness. However, it is unlikely that this type of bias could have led to a biased estimate of the association, as it should have affected both groups - with and without alcohol misuse. Third, only 39% of participants completed at least one follow-up questionnaire. However, this limitation is inherent to online cohort studies, particularly during a pandemic when young adults may have faced additional constraints (eg, isolation, increased mental burden). This low response rate may have led to an underestimation of the association between anxiety and alcohol misuse, as participants with moderate to severe anxiety were more likely to be lost to follow-up. It is also important to highlight that all participants were included in the statistical models, even if they did not have follow-up information. This increased the validity of the mixed model in the estimation of baseline parameters. Fourth, although the AUDIT-C is an internationally validated instrument and is widely recognised for its brevity and ease of use, it is essential to emphasise that it is a screening tool and not a diagnostic tool. A high score on the AUDIT-C indicates an increased likelihood of alcohol misuse but does not systematically confirm harmful use. Indeed, not all individuals identified as misusers by the AUDIT-C necessarily have a high-risk alcohol use or an alcohol use disorder, although they are more likely to have one. Fifth, alcohol misuse was only assessed at baseline, preventing us from examining bidirectional relationships through cross-lagged analyses. As a result, we could not explore whether anxiety symptoms contributed to changes in alcohol consumption over time. To mitigate this limitation, we first adjusted our models for a history of mental health disorders, which could confound the association between baseline alcohol misuse and subsequent anxiety symptoms. Second, we conducted a sensitivity analysis excluding participants with moderate-to-severe anxiety at baseline, ensuring that pre-existing anxiety did not drive baseline alcohol misuse.

Despite these limitations, the main strength of this study was its prospective design, with repeated measures over a period of almost 2 years after the first lockdown. This allowed the use of segmented linear mixed models, which require a large sample size and sufficient repeated measures. The segmentation highlighted some differences between the short and long term effects of the COVID-19 pandemic on anxiety. Comparing people without alcohol misuse to those with it, we found that among the latter, women and people with high perceived loneliness were more affected by the persistence of the pandemic over time than by its initial shock.

## Conclusions

Our current findings highlight the impact of a pre-existing alcohol misuse on the increase in anxiety after the second COVID-19 lockdown in France. We identified women and people with high perceived loneliness as groups particularly vulnerable to anxiety, with a worsening of their psychic state as the crisis persisted. Future studies should continue to explore changes in anxiety symptoms among people with alcohol misuse, to obtain a more comprehensive understanding of how the recent health crisis affected this specific population. Additionally, more research is needed to explore perceived loneliness or perceived social support among people with alcohol misuse.

Importantly, our current findings suggest that certain groups may benefit now from targeted psychological support in primary care or in addiction centres. Implementing a simple non-intrusive question about perceived loneliness in clinical practice could help to screen those at the greatest risk for high anxiety. In the event of another crisis leading to the implementation of social distancing measures, establishing group therapies or facilitating access to existing support associations would help improve mental health by strengthening the social support.

## Supplementary material

10.1136/bmjopen-2025-105567 online supplemental file 1

10.1136/bmjopen-2025-105567 online supplemental file 2

10.1136/bmjopen-2025-105567 online supplemental file 3

10.1136/bmjopen-2025-105567 online supplemental file 4

## Data Availability

Data are available upon reasonable request.
