# Supplementary material for: Effects of alcohol misuse on the evolution of anxiety during the COVID-19 pandemic in France: results from CONFINS cohort
Source: BMJ Open. 2026 Jan 6;16(1):e105567. doi: 10.1136/bmjopen-2025-105567 (PMC12778323; doi:10.1136/bmjopen-2025-105567)
Supplement: online supplemental file 2 [file bmjopen-16-1-s002.pdf]

Table S1. COVID-19 mitigation policies in France, 2020–2021.

	1st lockdown<br>**March 17 to May 11, 2020**	2nd lockdown<br>**October 30 to December 15, 2020**	3rd lockdown<br>**April 3 to May 3, 2021**
Schools/universities	Primary schools, secondary schools, and universities are closed	Only universities are closed but can re-open for exams only	Primary schools and secondary schools are closed, universities can be accessed once a week by students
Mandatory mask wearing in closed places	No	Yes	Yes
Travel restrictions	Travel is authorized for work or business travel, groceries, for health reasons, for family emergencies, for childcare, for individual physical activity, or for walking a pet	Travel is authorized for work or business travel, groceries, for health reasons, for family emergencies, for childcare, for individual physical activity, walking a pet, to accompany a child to school, walk near your home for an hour, visits family members in retirement homes and nursing homes, go to a judicial or administrative summons, or go to an exam	Same as 2nd lockdown
Curfew	No	Yes (9 pm–6 am)	Yes (6 pm–6 am)
Access to alcohol	Yes	Yes	Yes, but alcohol consumption is prohibited in public
Closure of public places	Closure of places of sociability and leisure, such as bars, restaurants, cafes, cinemas, casinos, and retail businesses, with the exception of pharmacies and grocery stores, establishments of worship are allowed to remain open but meetings or gatherings are prohibited	Same as 1st lockdown	Only businesses selling essential goods and services are allowed to open, including pharmacies and grocery stores but also bookstores, record stores, hardware stores, plant and flower shops, hairdressers, shoemakers, chocolatiers, car dealerships and real estate agencies
Financial aids	• Solidarity funds for closed small/medium enterprises<br>• Delays of social and fiscal charges for small/medium enterprises and self-employed professionals<br>• Rental support for small/medium enterprises<br>• Extended partial unemployment allocation		
Workplaces	Closure of all non-essential shops and businesses	Generalization of working from home when possible, work in factories, on farms, in construction and public works can proceed	Work from home was systematized wherever possible, for a minimum of 4 days per week
Closure of borders	Closure of the Schengen area borders	Closure of the European Union's external borders	Travel authorized with a mandatory negative PCR test dated within the past 72 hours
Access to the Covid-19 vaccines	No	No	Available for people 55 and over, healthcare workers, and people with comorbidities

Table S2. Comparison between participants without follow-up and those with at least one follow-up. CONFINS cohort, France, 2022.

Characteristics	General population N=1868		Without follow-up N=1139		With at least one follow-up N=729	
	n	%	n	%	n	%
Gender						
Man	396	21.2	243	21.3	153	21.0
Woman	1472	78.8	896	78.7	576	79.0
Age						
<25 years	806	43.1	555	48.7	251	34.4
≥25 years	1062	56.9	584	51.3	478	65.6
Marital status						
Single	752	40.3	492	43.2	260	35.7
In a relationship or married	1071	57.3	621	54.5	450	61.7
Divorced or widowed	45	2.4	26	2.3	19	2.6
Years of education post-high school diploma						
<2 years	275	14.7	174	15.3	101	13.9
2 years	279	14.9	181	15.9	98	13.4
>2 years	1232	66.0	743	65.2	489	67.1
Missing data	82	4.4	41	3.6	41	5.6
History of mental health disorders						
Yes	408	21.8	249	21.9	159	21.8
No	1415	75.7	860	75.5	555	76.1
Don't know	45	2.4	30	2.6	15	2.1
GAD-7 score	4 [1–9]		4 [2–9]		3 [1–7]	
Anxiety level						
Minimal	998	53.4	574	50.4	424	58.2
Mild	451	24.1	283	24.8	168	23.0
Moderate	247	13.2	153	13.4	94	12.9
Severe	172	9.2	129	11.3	43	5.9
Alcohol misuse						
Without	775	41.5	452	39.7	323	44.3
With	1093	58.5	687	60.3	406	55.7
AUDIT-C score: median [IQR*]	4 [3–5]		5 [3–5]		3 [2–5]	
Alcohol consumption in the past year						
Never	0	0.0	0	0.0	0	0.0
Less than monthly	326	17.5	198	17.4	128	17.6
Monthly	244	13.1	152	13.3	92	12.6
2–4 times per month	776	41.5	472	41.4	304	41.7
2–3 times per week	410	21.9	257	22.6	153	21.0
≥4 times per week	112	6.0	60	5.3	52	7.1
Number of standard drinks on a typical day when drinking in the past year						
1–2	1009	54.0	602	52.9	407	55.8
3–4	375	20.1	233	20.5	142	19.5
5–6	124	6.6	82	7.2	42	5.8
7–9	27	1.4	19	1.7	8	1.1
≥10	7	0.4	5	0.4	2	0.3
Missing data†	326	17.5	198	17.4	128	17.6
Consumption of at least six standard drinks on one occasion in the past year						
Never	455	24.4	255	22.4	200	27.4
Less than monthly	659	35.3	405	35.6	254	34.8
Monthly	317	17.0	205	18.0	112	15.4
Weekly	105	5.6	71	6.2	34	4.7
Daily or almost daily	6	0.3	5	0.4	1	0.1
Missing data†	326	17.5	198	17.4	128	17.6
Smoking status						
Smoker	445	23.8	303	26.6	142	19.5
Ex-smoker	200	10.7	119	10.4	81	11.1
Non-smoker	1223	65.5	717	62.9	506	69.4
Perceived loneliness						
High	418	22.4	268	23.5	150	20.6
Low	1443	77.2	864	75.9	579	79.4

Note: † Missing data for participants consuming alcohol less than once a month. * IQR: interquartile range

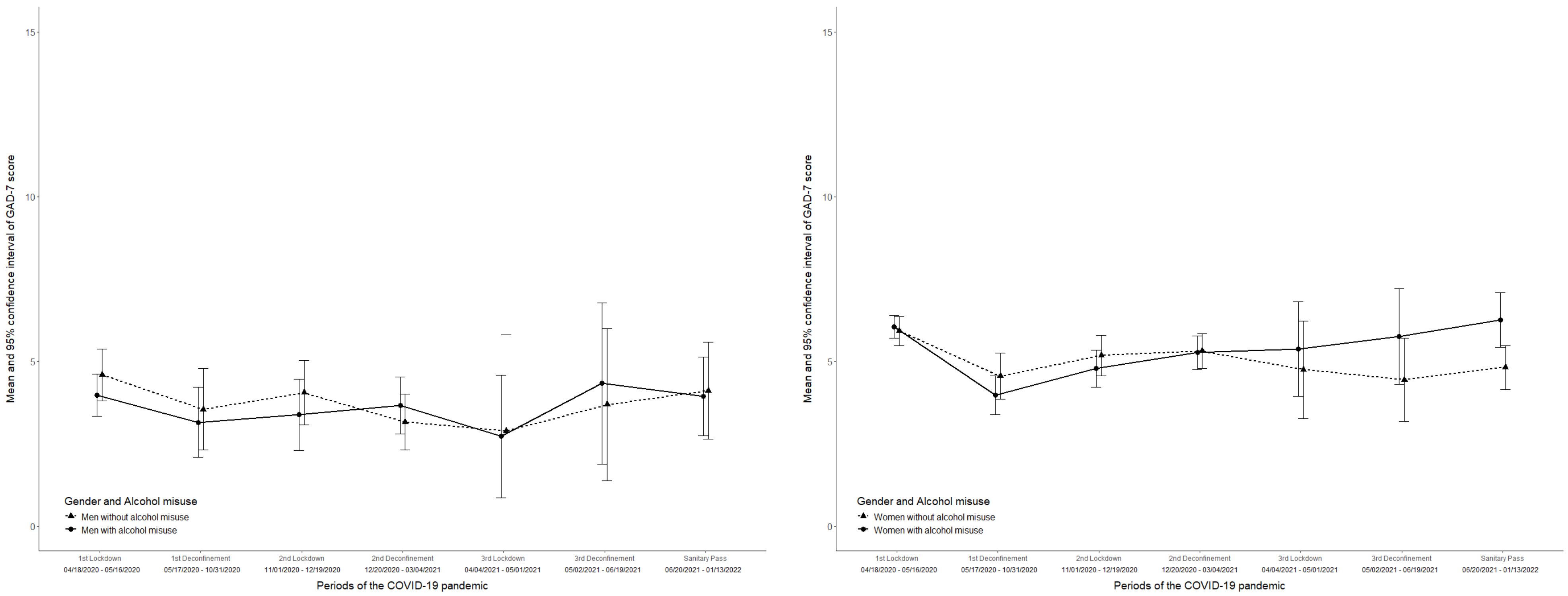

Fig. S1. Means and standard deviations of GAD-7 score through the different periods of the pandemic stratified by gender and alcohol misuse. CONFINS cohort, France, 2022.

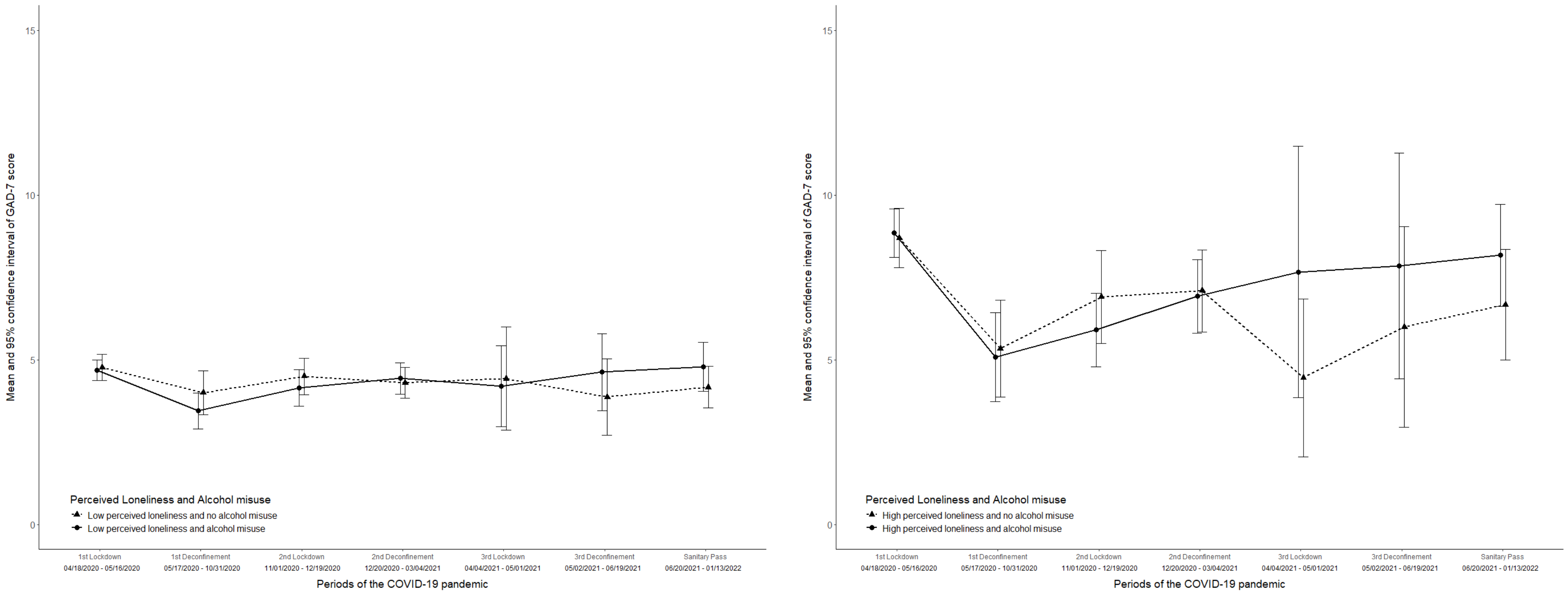

Fig. S2. Means and standard deviations of GAD-7 score through the different periods of the pandemic stratified by perceived loneliness and alcohol misuse. CONFINS cohort, France, 2022.

Table S3. Evolution of GAD-7 score stratified by gender, depending on the presence of alcohol misuse since the first lockdown, among participants with minimal-to-mild anxiety at baseline (N=1378). CONFINS cohort, France, 2022.

	**Women (n=1064)**								
	GAD-7 score at baseline			Change in GAD-7 score (point/trimester) until the end of the 1st deconfinement			Change in GAD-7 score (point/trimester) starting at the 2nd lockdown		
	β	95% CI	p value	β	95% CI	p value	β	95% CI	p value
Intercept/slope									
Without alcohol misuse	3.60	[3.05 ; 4.15]	<0.001***	0.54	[0.33 ; 0.75]	<0.001***	0.06	[-0.11 ; 0.24]	0.495
With alcohol misuse	3.69	[3.13 ; 4.25]	<0.001***	0.41	[0.23 ; 0.59]	<0.001***	0.30	[0.12 ; 0.47]	0.001**
Difference									
Without *vs* With alcohol misuse	0.09	[-0.31 ; 0.48]	0.664	-0.13	[-0.41 ; 0.14]	0.344	0.24	[-0.01 ; 0.48]	0.059*
	**Men (n=314)**								
	GAD-7 score at baseline			Change in GAD-7 score (point/trimester) until the end of the 1st deconfinement			Change in GAD-7 score (point/trimester) starting at the 2nd lockdown		
	β	95% CI	p value	β	95% CI	p value	β	95% CI	p value
Intercept/slope									
Without alcohol misuse	2.59	[1.89 ; 3.29]	<0.001***	0.20	[-0.17 ; 0.57]	0.297	0.01	[-0.31 ; 0.33]	0.940
With alcohol misuse	2.80	[2.12 ; 3.48]	<0.001***	0.39	[0.04 ; 0.75]	0.029**	-0.11	[-0.42 ; 0.20]	0.498
Difference									
Without *vs* With alcohol misuse	0.21	[-0.50 ; 0.92]	0.559	0.20	[-0.31 ; 0.71]	0.450	-0.12	[-0.57 ; 0.33]	0.600

Note: Models adjusted for age, perceived loneliness, mental health disorder history, smoking status, level of education, and marital status
*** p<0.01, **p<0.05, *p<0.10

Table S4. Evolution of GAD-7 score stratified by perceived loneliness, depending on the presence of alcohol misuse since the first lockdown, among participants with minimal-to-mild anxiety at baseline (N=1378). CONFINS cohort, France, 2022.

	High perceived loneliness (n=223)								
	GAD-7 score at baseline			Change in GAD-7 score (point/trimester) until the end of the 1st deconfinement			Change in GAD-7 score (point/trimester) starting at the 2nd lockdown		
	β	95% CI	p value	β	95% CI	p value	β	95% CI	p value
Intercept/slope									
Without alcohol misuse	3.86	[3.05 ; 4.67]	<0.001***	0.60	[0.15 ; 1.06]	0.010**	-0.34	[-0.73 ; 0.05]	0.091*
With alcohol misuse	3.97	[3.21 ; 4.73]	<0.001***	0.46	[0.07 ; 0.84]	0.019**	0.30	[-0.04 ; 0.64]	0.083*
Difference									
Without *vs* With alcohol misuse	0.11	[-0.73 ; 0.95]	0.798	-0.15	[-0.74 ; 0.45]	0.631	0.64	[0.12 ; 1.16]	0.016**
	**Low perceived loneliness (n=1155)**								
	GAD-7 score at baseline			Change in GAD-7 score (point/trimester) until the end of the 1st deconfinement			Change in GAD-7 score (point/trimester) starting at the 2nd lockdown		
	β	95% CI	p value	β	95% CI	p value	β	95% CI	p value
Intercept/slope									
Without alcohol misuse	2.56	[1.96 ; 3.17]	<0.001***	0.44	[0.24 ; 0.63]	<0.001***	0.12	[-0.04 ; 0.29]	0.149
With alcohol misuse	2.67	[2.06 ; 3.29]	<0.001***	0.40	[0.22 ; 0.58]	<0.001***	0.18	[0.01 ; 0.35]	0.040**
Difference									
Without *vs* With alcohol misuse	0.11	[-0.27 ; 0.49]	0.561	-0.04	[-0.30 ; 0.23]	0.792	0.05	[-0.18 ; 0.29]	0.657

Note: Models adjusted for gender, age, mental health disorder history, smoking status, level of education, and marital status
*** p<0.01, **p<0.05, *p<0.10.
