# Supplementary material for: Effects of alcohol misuse on the evolution of anxiety during the COVID-19 pandemic in France: results from CONFINS cohort
Source: BMJ Open. 2026 Jan 6;16(1):e105567. doi: 10.1136/bmjopen-2025-105567 (PMC12778323; doi:10.1136/bmjopen-2025-105567)
Supplement: online supplemental file 3 [file bmjopen-16-1-s003.pdf]

VACINFOVAV

Cohorte confins

Questionnaire annoté

v2.5 16/11/2020

Etude CONFINS

Date	Version	Commentaire
14/04/2020	v1.0	Questionnaire inclusion Population Générale + étudiants (en 2 parties)<br>retours utilisateurs sur la partie 1
15/04/2020	v1.1	Annotation questionnaire hebdo
05/05/2020	v1.2	Ajout question université
15/05/2020	V2.0	Questionnaire inclusion post confinement
22/05/2020	V2.1	Questionnaire à destination des étudiants et personnels de santé
15/06/2020	v2.2	Questionnaire déconfinement
28/08/2020	v2.3	Questionnaire déconfinement 2
28/09/2020	v2.4	Ajout partie perception santé dans questionnaire déconfinement 2
16/11/2020	v2.5	Mise à jour questionnaire inclusion reconfinement<br>mise à jour questionnaire de suivi reconfinement

Table des matières

Critères d'éligibilité 4

QUESTIONNAIRE INCLUSION - Première partie (période confinement) 5

PARTIE A. Vos données sociodémographiques 5

PARTIE B. Vos perceptions sur l'épidémie en cours 9

PARTIE C. Les consignes : les suivez-vous ? Qu'en pensez-vous ? 13

PARTIE D. Votre confinement 15

PARTIE E. Impact du confinement 18

PARTIE F. Votre avis sur traitements et vaccins 21

QUESTIONNAIRE INCLUSION - Seconde partie (période confinement) 25

PARTIE G. Votre profil médical 25

PARTIE H. Vos activités en confinement 28

PARTIE I. Vos perceptions sur la santé 30

PARTIE J. Vous gardez le moral ? 31

QUESTIONNAIRE SUIVI HEBDOMADAIRE (période confinement) 37

QUESTIONNAIRE INCLUSION - Première partie (période post confinement) 43

Critères d'éligibilité 43

PARTIE A. Vos données sociodémographiques 43

PARTIE B. Vos perceptions sur l'épidémie en cours 49

PARTIE C. Les consignes : les suivez-vous ? Qu'en pensez-vous ? 53

PARTIE D. Votre situation actuelle 55

PARTIE E. Impact de l'épidémie 56

PARTIE F. Votre avis sur traitements et vaccins 59

PARTIE G. Votre profil médical 60

PARTIE H. Vos activités 64

PARTIE I. Vos perceptions sur la santé 65

PARTIE J. Vous gardez le moral ? 66

Questions complémentaires à destination des étudiants en médecine et autres études en santé 70

Questionnaire DECONFINEMENT 1 77

Partie A. Vos perceptions sur l'épidémie en cours 77

Partie B. Votre quotidien face à l'épidémie 79

Partie C. Votre moral 81

Partie D. Votre avis sur les traitements et les vaccins 85

Questionnaire DECONFINEMENT 2 89
Partie A. Vos perceptions sur l'épidémie en cours 89
Partie B. Votre quotidien face à l'épidémie 91
Partie C. Votre moral 92
Partie D. Vos perceptions sur la santé 95
QUESTIONNAIRE INCLUSION - (période reconfinement nov) 97
Critères d'éligibilité 97
PARTIE A. Vos données sociodémographiques 97
PARTIE B. Vos perceptions sur l'épidémie en cours 102
PARTIE C. Les consignes : les suivez-vous ? Qu'en pensez-vous ? 107
PARTIE D. Votre confinement 110
PARTIE E. Impact de l'épidémie 111
PARTIE F. Votre avis sur traitements et vaccins 113
PARTIE G. Votre profil médical 117
PARTIE H. Vos activités 121
PARTIE I. Vos perceptions sur la santé 122
PARTIE J. Vous gardez le moral ? 123
Questionnaire SUIVI MENSUEL 127
Partie A. Vos perceptions sur l'épidémie en cours 127
Partie B. Votre quotidien face à l'épidémie 130
Partie C. Votre moral 132
Partie D. Votre confinement 136

Critères d'éligibilité

**Q1. Date de naissance :** |__|__|/ |__|__|__|__| (≥ 18 ans)<br>**Pays de naissance** : ☐ France ☐ Autre<br>*# Si France, #* Précisez la commune |__|__|__|__|__| (code postal)	naiss_date MMYY10.<br>naiss_pays pays.<br>naiss_commune 5.
**Q2. Êtes-vous confiné.e par mesures gouvernementales** :<br>☐ Oui ☐ Non (Oui)<br>**Dans quel pays êtes-vous confiné.e** :<br>☐ France<br>☐ Autre, préciser : ....<br>(France/autre)<br><br>*Patient éligible si :*<br>• *Age ≥ 18 ans*<br>• *Confiné*	confine ouinon.<br>redis_pays pays.<br>redis_pays_txt $100.

QUESTIONNAIRE INCLUSION - Première partie (période confinement)

PARTIE A. Vos données sociodémographiques

(page 1)

**A1. Sexe :** ☐ Masculin ☐ Féminin ☐ Autre<br><br>**A2. Taille : |__|__|__|cm** **Poids : |__|__|__|kg**<br><br>**A3. Votre situation de famille**<br>☐ Célibataire<br>☐ En couple (depuis au moins 3 mois) sans être marié.e ni pacsé.e<br>☐ Marié.e, Pacsé.e<br>☐ Autre (divorcé.e, veuf.ve)<br><br>**A4. Avez-vous des enfants ?**<br>☐ Oui ☐ Non<br># Si oui, # combien : |__|__|	sexe sexe.<br><br>taille 3.<br>poids 3.<br><br>situation_fam sitfam.<br><br>etude_enfant ouinon.<br><br>enfant_nb 2.
**A5. Quelle est votre filière d'études ?**<br>*#menu déroulant#*<br>1. Administration économique et sociale<br>2. Administration publique<br>3. Agronomie, agroalimentaire<br>4. Architecture, design<br>5. Arts (plastiques, du spectacle)<br>6. Commerce, vente<br>7. Droit<br>8. Economie et gestion<br>9. Electronique, énergie électrique, automatique<br>10. Génie civil<br>11. Géographie et aménagement<br>12. Histoire<br>13. Histoire de l'art et archéologie<br>14. Hôtellerie, restauration, tourisme<br>15. Information-communication<br>16. Informatique<br>17. Lettres, langues et civilisations étrangères et régionales<br>18. Mathématiques<br>19. Mathématiques et informatique appliquées aux sciences humaines et sociales	etude_filiere filet.

20. Mécanique<br>21. Médecine<br>22. Philosophie<br>23. Physique, chimie<br>24. Psychologie<br>25. Science politique<br>26. Sciences cognitives<br>27. Sciences de l'environnement<br>28. Sciences de la vie et de la Terre<br>29. Sciences de l'éducation<br>30. Sciences de l'homme, anthropologie, ethnologie<br>31. Sciences des matériaux<br>32. Sciences du langage<br>33. Sciences et techniques des activités physiques et sportives<br>34. Sciences et technologies<br>35. Sciences pour la santé autres que médecine<br>36. Sciences pour l'ingénieur<br>37. Sciences sanitaires et sociales<br>38. Sciences sociales<br>39. Télécommunications<br>40. Autre	
**A6. En quelle année d'études êtes-vous ?**<br>☐ 1ère ☐ 2ème ☐ 3ème ☐ 4ème ☐ 5ème ☐ > 5ème	etude_an annee.
**A7. Participez- vous à l'étude i-Share ?**<br>❒ Oui<br>❒ Non	Ishare ouinon.<br><br>Ishare_num $255.
**A8. Concernant la situation économique de la famille qui vous a élevé(e) durant votre enfance et adolescence diriez-vous qu'elle était :**<br>☐ Très confortable<br>☐ Confortable<br>☐ Correcte<br>☐ Difficile<br>☐ Très difficile	enfance_siteco siteco.
**A9. Quelle est votre <u>principale</u> source de revenus pour le financement de votre année universitaire ? *(Une seule réponse possible)***<br>☐ Famille<br>☐ Bourse(s)<br>☐ Activités rémunérées<br>☐ Autre (économies, épargnes, prêt étudiant)	etude_revenu revenu.

**A10. Quel est votre diplôme le plus élevé ?**<br>☐ Aucun<br>☐ BEPC (Brevet des collèges), BEP, CAP, BP (Brevet professionnel)<br>☐ Baccalauréat (général, technologique ou professionnel)<br>☐ Bac+2 (DEUG, BTS, DUT ou équivalent)<br>☐ Bac+3 à Bac+4 (Licence, Master 1, DEI ou équivalent)<br>☐ Bac+5 ou plus (Master 2, école d'ingénieur, doctorat ou équivalent)	niveau_etude nivet.
**A11. Quand vous étiez étudiant, avez-vous participé à l'étude i-Share ?**<br>☐ Oui ☐ Non	nonet_ishare ouinon.
**A12. Quelle est votre profession ?**<br>☐ Agriculteur.trice (ex. éleveur.se, viticulteur.trice)<br>☐ Artisan, commerçant.e, chef.fe d'entreprise (ex. électricien.ne à son compte, gérant.e)<br>☐ Profession intermédiaire (ex. professeur.e des écoles, technicien.ne, infirmièr.e)<br>☐ Cadre, ingénieur.e, profession libérale (ex. médecin, journaliste)<br>☐ Employé.e administratif d'entreprise ou de la fonction publique (ex. secrétaire, hôtesse de l'air/stewart, policier.e, aide-soignant.e)<br>☐ Employé.e de commerce (ex. caissièr.e, vendeur.se en magasin, pompiste)<br>☐ Personnel des services directs aux particuliers (ex. serveur.se, assistant.e maternelle)<br>☐ Ouvrier.e (ex. opérateur.e, chauffeur.e, peintre en bâtiment, magasinier.e)<br>☐ Vous n'avez pas de profession	profession prof.
# Si profession cochée (autre que « pas de profession ») #<br>Votre profession est-elle en rapport avec le domaine de la santé ?<br>☐ Oui ☐ Non	prof_sante ouinon.
# Si oui, # Etes-vous soignant au contact de malades ?<br>☐ Oui ☐ Non	prof_soigne ouinon.

**A13. Quelle était votre situation professionnelle <u>avant le confinement</u> ?**<br>☐ En CDI ou fonctionnaire<br>☐ En CDD ou autre contrat court (saisonnier, vacataire, intérim, pigiste, CESU)<br>☐ Indépendant (y compris auto-entrepreneur, en freelance, libéral)<br>☐ Stagiaire, volontaire ou apprenti.e<br>☐ Vous avez un petit boulot non déclaré (ex. babysitting)<br>☐ Vous avez un autre type de contrat de travail<br>☐ Sans travail et à la recherche d'un emploi<br>☐ Au foyer<br>☐ Vous ne travaillez pas actuellement et vous ne cherchez pas d'emploi<br>☐ Retraité	situation_pro sitpro.
**A14. Avant le début du confinement, où viviez-vous :**<br>☐ Dans une maison, un appartement<br>**A15. Précisez** :<br>☐ En couple<br>☐ Seul<br>☐ En colocation<br>☐ Autre : hôtel, caravane, voiture, squat…	etude_lieu lieuet<br><br>etude_lieu_seul seul.

PARTIE B. Vos perceptions sur l'épidémie en cours

(page 2/3)

M. 1. Pensez-vous ou savez-vous si vous êtes actuellement atteint(e) par le COVID-19 ?

covid_atteint
atteint.

- ☐ Oui, j'ai été testé(e) positif
- ☐ Oui, cela a été évoqué par un médecin mais je n'ai pas été testé
- ☐ C'est possible, je présente des symptômes (fièvre, toux, courbatures, fatigue intense, diarrhées, douleur thoracique, perte de l'odorat, gêne respiratoire)
- ☐ C'est peu probable, je ne me sens pas malade
- ☐ Je suis certain(e) de ne pas être atteint(e)
- ☐ Je ne sais pas

M. 2. Avez-vous déjà eu le COVID-19 et êtes-vous actuellement rétabli.e?

covid_sympt
ontest.

- ☐ Oui avec un diagnostic confirmé (test positif)
- ☐ Oui mais sans qu'un test ait été réalisé
- ☐ Non

M. 3. Y a-t-il, dans votre entourage ou votre famille, des personnes qui ont eu le COVID-19 ou des signes de maladie laissant à penser que c'était le COVID-19 ?

covid_fam
ouinon.

☐ Oui ☐ Non

M. 4. Dans le contexte de cette épidémie, quel est votre degré d'inquiétude à propos de :

	1<br>Pas du tout inquiet(ète)	2	3	4	5<br>Très inquiet(ète)	Non concerné(e)	
Votre santé (infection par le COVID-19)	❑	❑	❑	❑	❑	❑	inq_sante inquiet.
La santé de vos parents	❑	❑	❑	❑	❑	❑	inq_parent inquiet.
La santé d'un de vos proches, de vos amis	❑	❑	❑	❑	❑	❑	inq_amis inquiet.

Votre situation financière	❑	❑	❑	❑	❑	❑	inq_finance inquiet.
De manquer de quelque chose (par ex., médicaments, pain, …)	❑	❑	❑	❑	❑	❑	inq_manque inquiet.
De manquer de soutien moral et affectif	❑	❑	❑	❑	❑	❑	inq_soutien inquiet.

M. 5. *# Si profession cochée (autre que « pas de profession ») #* **Indiquez votre degré d'inquiétude par rapport à votre emploi (fin de CDD, licenciement,…) :**
Donnez une note entre 0 (pas du tout inquiet(ète)) et 10 (très inquiet(ète)).

inq_travail likert.

0 1 2 3 4 5 6 7 8 9 10

M. 6. **Indiquez votre degré d'inquiétude par rapport à vos études (report des examens, validation de l'année, report de la rentrée, report des soutenances…) :**

Donnez une note entre 0 (pas du tout inquiet(ète)) et 10 (très inquiet(ète)).

inq_etude likert.

0 1 2 3 4 5 6 7 8 9 10

M. 7. **A quel degré situez-vous la dangerosité du COVID-19 pour vous-même ou vos proches ?**
Donnez une note entre 0 (pas dangereux) et 10 (très dangereux).

inq_danger likert.

0 1 2 3 4 5 6 7 8 9 10

M. 8. **Que pensez-vous de l'évolution de l'épidémie ? (Plusieurs réponses possibles)**

- ☐ Tout va rentrer dans l'ordre dans les semaines qui viennent et nous reprendrons nos vies comme avant — epidemie_evol1 coch.
- ☐ L'épidémie va durer plusieurs mois et nos vies seront toujours très perturbées — epidemie_evol2 coch.
- ☐ Après l'épidémie, nous reprendrons nos vies comme avant — epidemie_evol3 coch.
- ☐ Après l'épidémie, nos vies seront toujours très perturbées — epidemie_evol4 coch.
- ☐ Sans opinion — epidemie_evol5 coch.

**Q. Selon vous, les affirmations suivantes sont-elles vraies ou fausses ?**	
M. 9. Le virus du COVID-19 a été fabriqué dans un laboratoire<br>☐ Vrai ☐ Faux ☐ Je ne sais pas	fakenews1<br>vraifaux.
M. 10. Le virus peut se transmettre par l'air<br>☐ Vrai ☐ Faux ☐ Je ne sais pas	fakenews2<br>vraifaux.
M. 11. Seules les personnes âgées de plus de 70 ans peuvent décéder du COVID-19<br>☐ Vrai ☐ Faux ☐ Je ne sais pas	fakenews4<br>vraifaux.
M. 12. Toutes les personnes contaminées par le virus développent des symptômes<br>☐ Vrai ☐ Faux ☐ Je ne sais pas	fakenews6<br>vraifaux.
M. 13. Boire des boissons très chaudes permet d'éviter la contamination par le virus<br>☐ Vrai ☐ Faux ☐ Je ne sais pas	fakenews8<br>vraifaux.
M. 14. Le virus ne peut être transmis que par des personnes qui ont des symptômes comme de la fièvre et de la toux<br>☐ Vrai ☐ Faux ☐ Je ne sais pas	fakenews5<br>vraifaux.
M. 15. Le virus survit sur des surfaces inertes comme les poignées de porte ou les barres dans les transports en commun<br>☐ Vrai ☐ Faux ☐ Je ne sais pas	fakenews3<br>vraifaux.
M. 16. Tout le monde devrait porter un masque pour se protéger efficacement du virus<br>☐ Vrai ☐ Faux ☐ Je ne sais pas	fakenews7<br>vraifaux.
M. 17. **Est-ce que vous suivez volontairement l'actualité en lien avec le COVID-19 ? (Lire le journal, consulter des nouvelles sur internet, regarder les nouvelles à la télévision)**<br>☐ Oui ☐ Non<br>M. 18. **A quelle fréquence avez-vous des discussions au sujet du COVID-19 ?**	suivi_actu<br>ouinon.

☐ Moins d'une fois par semaine<br>☐ Quelques fois par semaine mais pas tous les jours<br>☐ 1 fois par jour<br>☐ 2 à 4 fois par jour<br>☐ 5 à 9 fois par jour<br>☐ 10 fois et plus par jour	covid_discut<br>freqfois.
**Quelle durée par jour consultez-vous chacune des sources de nouvelles ou moyen de communications suivants ? (pour l'actualité en lien avec le COVID-19)**<br>M. 19. **Média traditionnels (Journal, Radio, Télévision etc.)**<br>☐ Jamais<br>☐ Moins d'une heure par jour<br>☐ 1 à 3 heures par jour<br>☐ 4 à 8 heures par jour<br>☐ > 8 heures par jour	actu_media<br>freqjour.
M. 20. **Fil d'actualité de réseaux sociaux et sites web (Facebook, Twitter, etc.)**<br>☐ Jamais<br>☐ Moins d'une heure par jour<br>☐ 1 à 3 heures par jour<br>☐ 4 à 8 heures par jour<br>☐ > 8 heures par jour	actu_web<br>freqjour.

PARTIE C. Les consignes : les suivez-vous ? Qu'en pensez-vous ?

(page 4)

**C1.** **Si vous n'êtes pas obligé d'aller sur votre lieu de travail, respectez-vous strictement les consignes de confinement telles que préconisées par l'état ?**	respect_cons1 ech5.

1 Pas du tout	2	3	4	5 Tout à fait	Non concerné(e)
❑	❑	❑	❑	❑	❑

**C2.** **Quand vous faites des courses ou sur votre lieu de travail, gardez-vous une distance d'au moins un mètre entre vous et d'autres personnes ?**	respect_cons2 ech5.

1 Pas du tout	2	3	4	5 Tout à fait	Non concerné(e)
❑	❑	❑	❑	❑	❑

**C3.** **Vous lavez-vous plus fréquemment les mains que d'habitude ?**	respect_cons3 ech5.

1 Pas du tout	2	3	4	5 Tout à fait	Non concerné(e)
❑	❑	❑	❑	❑	❑

Q. A quelle fréquence êtes-vous sorti(e) de votre domicile ces 7 derniers jours ?

		Plusieurs fois par jour	Une fois par jour	2 à 5 fois cette semaine	Une fois cette semaine	Aucu sort	
C4.	Pour aller dans votre lieu de travail/stage	❑	❑	❑	❑	❑	freq_sortie1 freqsem.
C5.	Pour des achats de première nécessité	❑	❑	❑	❑	❑	freq_sortie2 freqsem.
C6.	Pour une consultation ou des soins	❑	❑	❑	❑	❑	freq_sortie3 freqsem.
C7.	Pour motif familial impérieux, pour l'assistance aux personnes vulnérables ou la	❑	❑	❑	❑	❑	freq_sortie4 freqsem.

	**garde d'enfants**						
**C8.**	**Convocation judiciaire ou administrative**	❑	❑	❑	❑	❑	freq_sortie5 freqsem.
**C9.**	**Pour l'activité physique individuelle**	❑	❑	❑	❑	❑	freq_sortie6 freqsem.
**C10.**	**Pour les besoins des animaux de compagnie**	❑	❑	❑	❑	❑	freq_sortie7 freqsem.
**C11.**	**Pour une activité non couverte par les autorisations exceptionnelles de déplacement**	❑	❑	❑	❑	❑	freq_sortie8 freqsem.

C12. Diriez-vous que la réaction du gouvernement face à l'épidémie actuelle est :

- ☐ Trop exagérée
- ☐ Plutôt exagérée
- ☐ Appropriée
- ☐ Plutôt insuffisante
- ☐ Bien trop insuffisante

reaction_gouv reac.

C13. Diriez-vous que la réaction de la population face à l'épidémie actuelle est :

- ☐ Trop exagérée
- ☐ Plutôt exagérée
- ☐ Appropriée
- ☐ Plutôt insuffisante
- ☐ Bien trop insuffisante

reaction_pop reac.

C14. Trouvez-vous, à titre personnel, que les mesures de confinement sont faciles à respecter ?

- ☐ Oui, tout à fait
- ☐ Oui, plutôt
- ☐ Non, plutôt pas
- ☐ Non, pas du tout

mesure_facil ech4.

C15. Faites-vous confiance aux pouvoirs publics pour contrôler l'épidémie de COVID-19 ?

Donnez une note entre 0 (pas du tout confiance) et 10 (tout à fait confiance).

conf_controle likert.

0 1 2 3 4 5 6 7 8 9 10<br><br>**C16. Faites-vous confiance aux pouvoirs publics pour vous informer sur le COVID-19 ?**<br>*Donnez une note entre 0 (pas du tout confiance) et 10 (tout à fait confiance).*<br><br>0 1 2 3 4 5 6 7 8 9 10<br><br>**Exprimez-vous : Qu'est-ce que vous pensez des mesures prises par les autorités publiques concernant le confinement ?**<br>*Nous vous proposons de vous exprimer librement en quelques mots ou quelques phrases.*	conf_info likert.<br><br>exprime_autorite $3000.

PARTIE D. Votre confinement

(Page 5/6)

**D1. Département du lieu de confinement : |__|__|**<br>*(si confiné(e) hors France, renseigner « 00 »)*	conf_dep 3.
**D2. Taille de la commune du lieu de confinement :**<br>☐ Zone rurale<br>☐ <5000 habitants<br>☐ 5 000 à 20 000 habitants<br>☐ 20 000 à 100 000 habitants<br>☐ > 100 000 habitants<br>☐ Ile de France	conf_commune taille.
**D3. Quel est votre lieu de confinement ?**<br>☐ Mon domicile habituel :	conf_lieu conflieu.<br><br>conf_lieu_type lieutype.

☐ un appartement<br>☐ une maison<br>☐ une chambre en cité universitaire ou foyer<br>☐ Autre : hôtel, caravane, voiture, squat…<br><br>☐ Un autre endroit :<br>☐ un appartement<br>☐ une maison<br>☐ une chambre en cité universitaire ou foyer<br>☐ Autre : hôtel, caravane, voiture, squat…	`conf_lieu_type lieutype.`
**D4. Quelle est la surface de votre lieu de confinement ?**<br>I_I_I_I m^2	`conf_lieu_surf 4.`
**Votre lieu de confinement dispose t'il :**<br>**D5.** D'un **balcon** ☐ Oui ☐ Non<br>**D6.** D'une **terrasse** ☐ Oui ☐ Non<br>**D7.** D'un **jardin** ☐ Oui ☐ Non<br>**D8.** De **fenêtres** avec la lumière directe du jour ☐ Oui ☐ Non	`conf_balcon ouinon.`<br>`conf_terrase ouinon.`<br>`conf_jardin ouinon.`<br>`conf_fenetre ouinon.`
**D9. Disposez-vous d'une connexion Internet ?**<br>☐ Oui ☐ Non<br><br># Si oui, # est-elle suffisamment performante :<br>**D10. Dans le cadre de votre travail ou de vos études ?** ☐ Oui ☐ Non ☐ Non concerné(e)<br>**D11. Afin de maintenir un contact visuel (type Skype) avec vos proches ?** ☐ Oui ☐ Non ☐ Non concerné(e)<br>**D12. Pour vous divertir (téléchargement séries ou musique, streaming) ?** ☐ Oui ☐ Non ☐ Non concerné(e)	`conf_internet ouinon.`<br>`conf_int_teletr onnc.`<br>`conf_int_contact onnc.`<br>`conf_int_loisir onnc.`
**D13. Nombre de personnes avec qui vous êtes confiné.e :**<br>☐ Je suis seul(e)<br>☐ 1 ☐ 2 ☐ 3 ☐ 4 ☐ 5 ☐ plus de 5<br><br>**D14.** # Si nombre de personnes ≥ 1, # **Ces personnes sont :** *(plusieurs réponses possibles)*<br>☐ Des membres de ma famille :<br>☐ Mon conjoint<br>☐ Des enfants (les miens ou ceux de mon cercle familial)	`conf_nbpers nbpers2.`<br>`conf_famille coch.`<br>`conf_conjoint coch.`<br>`conf_enfant coch.`

Pour ajouter un enfant cliquer sur (+). Pour les enfants e moins de 1 an, renseigner 0.<br>Age de l'enfant : ___<br>Age de l'enfant : ___<br>(+)<br><br>☐ Vos parents<br>☐ Mes frère(s)/ soeur(s)<br>☐ Autre(s) membre(s) de la famille élargie<br><br>☐ Des amis ou colocataires<br><br>**A l'intérieur de votre lieu de confinement, disposez-vous pour vous-même de :**<br>D15.Un **smartphone** ☐ Oui ☐ Non<br>D16.Une **tablette** ☐ Oui ☐ Non<br>D17.Un **ordinateur** ☐ Oui ☐ Non<br>D18.Une **liseuse** électronique ☐ Oui ☐ Non<br>D19.Une **télévision** ☐ Oui ☐ Non<br><br>**D20.Avez-vous un animal de compagnie ?**<br>☐ Oui ☐ Non	enfant_age 2.<br><br>conf_pere coch.<br>conf_frere coch.<br>conf_autrefam coch.<br><br>conf_amis coch.<br><br>conf_phone ouinon.<br>conf_tabl ouinon.<br>conf_ordi ouinon.<br>conf_liseuse ouinon.<br>conf_tele ouinon.<br><br>conf_animal ouinon.
**D21.Quelle est votre situation professionnelle <u>actuelle</u> :**<br>☐ Vous travaillez normalement<br>☐ Vous télétravaillez<br>☐ Vous avez diminué votre temps de travail ou ne travaillez que sur certains lieux<br>☐ Vous êtes au chômage technique ou partiel<br>☐ Vous êtes en congé forcé<br>☐ Vous êtes en arrêt de travail pour garde d'enfants<br>☐ Vous êtes en arrêt de travail pour maladie<br>☐ Vous avez perdu votre emploi du fait de la crise sanitaire<br>☐ Autre<br><br>**D22.Si vous devez travailler de chez vous, comment évaluez-vous votre capacité à effectuer les tâches qui sont attendues de vous dans votre environnement actuel ?**<br>☐ Je suis loin de fournir le travail attendu et c'est un problème pour moi	situation_act sitact.<br><br>eval_travail evaltr.

☐ Je ne suis pas aussi performant(e)/concentré(e) que d'habitude mais j'arrive à travailler<br>☐ Je suis satisfait(e) de ce que j'arrive à faire<br>☐ Je travaille plutôt mieux que d'habitude<br>☐ *Je ne suis pas concerné(e) par cette question*	
**D23.** **<u>Au cours des 7 derniers jours</u>, avez-vous pratiqué une activité physique <u>en intérieur</u> (gymnastique, vélo d'appartement)**<br>☐ Non<br>☐ Oui mais pas tous les jours<br>☐ Oui tous les jours	sport_int tsjours.
**<u>Au cours des 7 derniers jours</u>, avez-vous pratiqué une activité physique <u>en extérieur</u> (marche dehors,…)**<br>☐ Non<br>☐ Oui mais pas tous les jours<br>☐ Oui tous les jours	sport_ext tsjours.

PARTIE E. Impact du confinement

(page 7/8)

Q. <u>Au cours des 7 derniers jours</u>, à quelle fréquence avez-vous été dérangé(e) par les problèmes suivants ?

		Presque jamais	Plusieurs jours	Plus de la moitié du temps	Presque tous les jours	
**E1.**	Peu d'intérêt ou de plaisir à faire les choses	❑	❑	❑	❑	pb_derang1 freq4.
**E2.**	Se sentir triste, déprimé(e) ou désespéré(e)	❑	❑	❑	❑	pb_derang2 freq4.
**E3.**	Difficultés à s'endormir ou à rester endormi(e), ou trop dormir	❑	❑	❑	❑	pb_derang3 freq4.
**E4.**	Se sentir fatigué(e) ou avoir peu d'énergie	❑	❑	❑	❑	pb_derang4 freq4.

**E5.**	Peu d'appétit ou trop manger	❑	❑	❑	❑	pb_derang5 freq4.
**E6.**	Mauvaise perception de vous-même — ou vous pensez que vous êtes un perdant ou que vous n'avez pas satisfait vos propres attentes ou celles de votre famille	❑	❑	❑	❑	pb_derang6 freq4.
**E7.**	Difficultés à se concentrer sur des choses telles que lire le journal ou regarder la télévision	❑	❑	❑	❑	pb_derang7 freq4.
**E8.**	Vous bougez ou parlez si lentement que les autres personnes ont pu le remarquer. Ou au contraire – vous êtes si agité(e) que vous bougez beaucoup plus que d'habitude	❑	❑	❑	❑	pb_derang8 freq4.
**E9.**	Vous avez pensé que vous seriez mieux mort(e) ou vous avez pensé à vous blesser d'une façon ou d'une autre	❑	❑	❑	❑	pb_derang9 freq4.

Q. <u>Au cours des 7 derniers jours</u>, à quelle fréquence avez-vous été gêné(e) par les problèmes suivants ?

		Presque jamais	Plusieurs jours	Plus de la moitié du temps	Presque tous les jours	
**E10.**	Un sentiment de nervosité, d'anxiété ou de tension	❑	❑	❑	❑	pb_gene1 freq4.
**E11.**	Une incapacité à arrêter de s'inquiéter ou à contrôler ses inquiétudes	❑	❑	❑	❑	pb_gene2 freq4.
**E12.**	Une inquiétude excessive à propos de différentes choses	❑	❑	❑	❑	pb_gene3 freq4.
**E13.**	Des difficultés à me détendre	❑	❑	❑	❑	pb_gene4 freq4.
**E14.**	Une agitation telle qu'il m'est difficile de tenir en	❑	❑	❑	❑	pb_gene5

	place					freq4.
**E15.**	Une tendance à être facilement contrarié(e) ou irritable	❑	❑	❑	❑	pb_gene6 freq4.
**E16.**	Un sentiment de peur comme si quelque chose de terrible risquait de se produire	❑	❑	❑	❑	pb_gene7 freq4.

PARTIE F. Votre avis sur traitements et vaccins

(page 9/10)

**F1. Si vous étiez atteint.e du COVID-19, seriez-vous prêt.e à prendre un traitement dont l'efficacité n'est pas certaine ??**<br>☐ Oui, même s'il y a un risque à prendre ce traitement<br>☐ Oui, mais à condition que les risques liés au traitement soient faibles<br>☐ Oui, mais à condition qu'il n'y ait absolument aucun risque lié au traitement, même faible<br>☐ Non, jamais<br>☐ Je ne sais pas	trait_prendre prendre.
**F2. Quelle est votre opinion sur l'exposition médiatique de l'hydroxychloroquine, présentée par les équipes du Dr. Raoult comme un traitement potentiel du COVID-19 ?**<br>☐ Je n'ai pas d'opinion sur le sujet, c'est aux experts de s'exprimer<br>☐ Je pense qu'il faut davantage de temps et de recherche pour pouvoir conclure<br>☐ Je pense qu'on empêche le développement de cette solution pour des raisons financières ou politiques<br>☐ Je ne sais pas	trait_chloroquine chloroq.
**F3. Pensez-vous que les traitements de médecine alternative ou homéopathique sont efficaces contre le COVID-19 ?**<br>☐ Oui ☐ Non ☐ Je ne sais pas	trait_homeo ouinon.
**F4. Avez-vous confiance dans le fait qu'un traitement efficace contre le COVID-19 sera trouvé ?**<br>☐ Oui, dans quelques mois<br>☐ Oui, dans quelques années<br>☐ Non<br>☐ Je ne sais pas	trait_covid trait.
**F5. Seriez-vous prêt à vous faire vacciner contre le COVID-19 même si le vaccin n'a pas encore fait complètement la preuve de son efficacité ?**<br>☐ Oui, même s'il y a un risque lié au vaccin<br>☐ Oui, mais à condition que le risque lié au vaccin soit faible<br>☐ Oui, mais à condition qu'il n'y ait absolument aucun risque lié au vaccin, même faible<br>☐ Non, jamais<br>☐ Je ne sais pas	vaccovid vaccovid.

**F6. Si un vaccin contre le COVID-19 était produit dans 12 mois, alors que l'épidémie actuelle serait passée mais qu'il y aurait un risque qu'elle revienne chaque année comme la grippe, iriez-vous vous faire vacciner ?**	vaccovid_an cert.

1<br>Pas du tout	2	3	4	5<br>Certainement
❑	❑	❑	❑	❑

Les questions suivantes concernent la vaccination en général. Elles sont importantes pour comprendre vos comportements en matière de prévention de maladies infectieuses comme le COVID-19.

F7. Vous faites-vous vacciner régulièrement contre la grippe ? vacgrippe ouinon

☐ Oui ☐ Non

F8. Etes-vous à jour de vos vaccinations ? vacautre onnsp.

☐ Oui ☐ Non ☐ Je ne sais pas

F9. Avez-vous peur des effets secondaires des vaccins ? vaccin_effsec abs.

☐ Oui, tout à fait ☐ Pas vraiment ☐ Pas du tout

F10. Trouvez-vous facile de se renseigner sur la vaccination ? vac_facile facile.

☐ Très facile ☐ Facile ☐ Difficile ☐ Très difficile

☐ Je ne me suis pas renseigné(e) sur la vaccination

F11. Préférez-vous ne pas vous faire vacciner car vous pensez ne pas avoir assez de connaissance sur la vaccination et ses risques ? vac_nonconnu accord4.

☐ Tout à fait d'accord ☐ D'accord ☐ Pas d'accord ☐ Pas du tout d'accord

		Entiè-rement d'accord	Plutôt d'accord	Plutôt pas d'accord	Pas d'accord	Sans opinion	
F12.	La vaccination peut provoquer de graves effets secondaires	❑	❑	❑	❑	❑	vac_effsec accord5.
F13.	La vaccination n'a pas beaucoup d'intérêt, car il y a très peu de risque d'être infecté.e	❑	❑	❑	❑	❑	vac_nointeret accord5.

**F14.**	**Il n’est pas nécessaire d’être vacciné.e car beaucoup de gens le sont autour de nous**	❑	❑	❑	❑	❑	vac_nonec accord5.
**F15.**	**Si un vaccin n'est pas obligatoire c'est qu'il n'est pas si important**	❑	❑	❑	❑	❑	vac_nonoblig accord5.
**F16.**	**Les industries pharmaceutiques incitent à se faire vacciner pour augmenter leurs bénéfices**	❑	❑	❑	❑	❑	vac_benef accord5.
**F17.**	**Se faire vacciner soi-même a un impact sur la santé des autres**	❑	❑	❑	❑	❑	vaccin_impact accord5.

F18. Selon vous, quelle est l'importance d'internet comme source d'informations sur la vaccination ?

1<br>Pas important du tout	2	3	4	5<br>Très important
❑	❑	❑	❑	❑

vacinternet import.

F19. Quelle ressource sur internet consultez-vous le plus souvent pour vous renseigner sur la vaccination ? *(Une seule case à cocher)*

☐ Journaux en ligne
☐ Sites gouvernementaux (Vaccination Info Service, …)
☐ Sites d’institutions de santé (Santé publique France, …)
☐ Réseaux Sociaux
☐ Forums
☐ Plateformes de vidéos (YouTube, …)
☐ Autres

vac_source source.

F20. Les informations concernant la vaccination que vous trouvez sur les réseaux sociaux et les forums vous semblent-elles compréhensibles ?

☐ Absolument ☐ Le plus souvent ☐ Pas vraiment ☐ Pas du tout
☐ Je n’ai jamais consulté d’information sur les vaccins sur les réseaux sociaux et les forums

vacinfo_web_comp absweb.

F21. En général, les informations concernant la vaccination données sur les sites gouvernementaux sont-elles compréhensibles ?

vacinfo_gouv_comp absgouv.

☐ Absolument ☐ Le plus souvent ☐ Pas vraiment ☐ Pas du tout<br>☐ Je n'ai jamais consulté d'information sur les vaccins sur les sites gouvernementaux<br><br>**F22. Pensez-vous être capable de reconnaître les *fake news* sur le thème de la vaccination ?**<br>☐ Absolument ☐ Le plus souvent ☐ Pas vraiment ☐ Pas du tout<br><br>**F23. Avez-vous confiance concernant les informations sur la vaccination des sites gouvernementaux ?**<br>☐ Absolument ☐ Le plus souvent ☐ Pas vraiment ☐ Pas du tout<br>☐ Je n'ai jamais consulté d'information sur les vaccins sur les sites gouvernementaux<br><br>**F24. Pensez-vous que les informations sur la vaccination que vous trouvez sur les réseaux sociaux sont valides ?**<br>☐ Absolument ☐ Le plus souvent ☐ Pas vraiment ☐ Pas du tout<br>☐ Je n'ai jamais consulté d'information sur les vaccins sur les réseaux sociaux et les forums<br><br>**F25. Lorsque vous prenez connaissance d'une information sur la vaccination, croisez-vous plusieurs sources afin de vérifier sa validité ?**<br>☐ Toujours ☐ Souvent ☐ Rarement ☐ Jamais<br><br>**F26. Pensez-vous que les informations trouvées en ligne peuvent influencer votre choix de vous faire vacciner ?**<br>☐ Tout à fait d'accord ☐ D'accord ☐ Sans opinion ☐ Pas d'accord ☐ Pas du tout d'accord	vacinfo_fakenews absfake.<br><br>vacinfo_gouv_conf absgouv.<br><br>vacinfo_gouv_valid absweb.<br><br>vacinfo_source tjr.<br><br>vacinfo_influe accord5.
**F27. Exprimez-vous : Que pensez-vous de la vaccination comme mesure pour prévenir les maladies comme le COVID-19 ?**<br>*Nous vous proposons de vous exprimer librement en quelques mots ou quelques phrases.*	exprime_vaccin $3000.

QUESTIONNAIRE INCLUSION - Seconde partie (période confinement)

PARTIE G. Votre profil médical

**G1. Avant le début du confinement, comment caractériseriez-vous votre santé ?**<br>❒ Très bonne<br>❒ Bonne<br>❒ Moyenne<br>❒ Mauvaise<br>❒ Très mauvaise<br><br>**G2. Avant le début du confinement, quelle note donneriez-vous à votre qualité de vie ?**<br>*(0 pour la pire qualité de vie possible et 10 pour la meilleure qualité de vie possible)*<br>0 1 2 3 4 5 6 7 8 9 10<br><br>**G3. Avant le début du confinement, diriez-vous que vous êtes souvent inquiet.ète ou stressé.e ?**<br>*Donnez une note entre 0 (no stress) et 10 (très stressé(e)).*<br>0 1 2 3 4 5 6 7 8 9 10<br><br>**G4. Est-ce que vous tendez à rebondir rapidement après des moments difficiles ?**<br>☐ Non, pas du tout<br>☐ Non, pas vraiment<br>☐ Neutre<br>☐ Plutôt oui<br>☐ Oui, tout à fait	sante_avant evalsante.<br><br>qdv_avant likert.<br><br>stress_normal likert.<br><br>rebondir onneutre.
**G5. Au cours de votre vie, un médecin a-t-il déjà diagnostiqué chez vous un ou**	

**plusieurs problèmes de santé suivants ?**	
Maladie cardio-vasculaire (infarctus du myocarde, AVC,…)<br>☐ Oui ☐ Non ☐ Ne sait pas<br>**# Si oui, #** êtes-vous actuellement traité ? ☐ Oui ☐ Non	atcd_cardio onnsp.<br>atcd_cardio_tt ouinon.
Hypertension artérielle<br>☐ Oui ☐ Non ☐ Ne sait pas<br>**# Si oui, #** êtes-vous actuellement traité ? ☐ Oui ☐ Non	atcd_hta onnsp.<br>atcd_hta_tt ouinon.
Diabète<br>☐ Oui ☐ Non ☐ Ne sait pas<br>**# Si oui, #** êtes-vous actuellement traité ? ☐ Oui ☐ Non	atcd_diab onnsp.<br>atcd_diab_tt ouinon.
Maladie digestive chronique (ex. Crohn, ulcère)<br>☐ Oui ☐ Non ☐ Ne sait pas<br>**# Si oui, #** êtes-vous actuellement traité ? ☐ Oui ☐ Non	atcd_digest onnsp.<br>atcd_digest_tt ouinon.
Cancer<br>☐ Oui ☐ Non ☐ Ne sait pas<br>**# Si oui, #** êtes-vous actuellement traité ? ☐ Oui ☐ Non	atcd_cancer onnsp.<br>atcd_cancer_tt ouinon.
Asthme ou autre problème respiratoire<br>☐ Oui ☐ Non ☐ Ne sait pas<br>**# Si oui, #** êtes-vous actuellement traité ? ☐ Oui ☐ Non	atcd_respi onnsp.<br>atcd_respi_tt ouinon.
Un problème de santé mentale (dépression, trouble bipolaire, anxiété généralisée…)<br>☐ Oui ☐ Non ☐ Ne sait pas<br>**# Si oui, #** êtes-vous actuellement traité ? ☐ Oui ☐ Non	atcd_psy onnsp.<br>atcd_psy_tt ouinon.
Autre(s) problème(s) de santé<br>☐ Oui ☐ Non ☐ Ne sait pas<br>**# Si oui, #** êtes-vous actuellement traité ? ☐ Oui ☐ Non	atcd_autre onnsp.<br>atcd_autre_tt ouinon.
**Les questions suivantes [illegible] tabac et d'alcool <u>au</u>**	

<u>cours de l'année écoulée</u> et pas seulement les dernières semaines.<br><br>**G6. Concernant votre consommation de tabac au cours de l'année écoulée, êtes-vous :**<br><br>☐ Fumeur.se régulier.e de tabac (au moins une cigarette par jour)<br>☐ Fumeur.se occasionnel.le de tabac (moins d'une cigarette par jour)<br>☐ Ex-fumeur.se régulier.e de tabac (au moins une cigarette par jour)<br>☐ Non-fumeur.se (vous n'avez jamais fumé une cigarette par jour)	avant_tabac fumeur.
**G7. Avez-vous utilisé une cigarette électronique au cours de l'année écoulée ?**<br><br>☐ Non<br>☐ Parfois<br>☐ Tous les jours	avant_ecig nonpfs.
**G8. Combien de fois vous est-il arrivé de consommer de l'alcool au cours de l'année écoulée ?**<br><br>☐ Jamais<br>☐ Moins d'une fois par mois<br>☐ Une fois par mois<br>☐ 2 à 4 fois par mois<br>☐ 2 à 3 fois par semaine<br>☐ 4 fois ou plus par semaine	avant_alcool freqmois.
**G9.** #Si alcool au moins une fois par mois, # **Combien de verres standard buvez-vous au cours d'une journée ordinaire où vous buvez de l'alcool ?**<br><br>*Un verre d'alcool correspond à un ballon de vin (10 cl), un demi de bière (25 cl) ou un verre d'alcool fort (4 cl).*<br><br>☐ Un ou deux<br>☐ Trois ou quatre<br>☐ Cinq ou six<br>☐ Sept à neuf<br>☐ Dix ou plus	avant_verre nbverre.
**G10.** #Si alcool au moins une fois par mois, # **Au cours d'une même occasion, combien de fois vous est-il arrivé de boire 6 verres standard ou plus (au cours de l'année écoulée) ?**<br><br>☐ Jamais<br>☐ Moins de 1 fois par mois<br>☐ 1 fois par mois<br>☐ Une fois par semaine<br>☐ Chaque jour ou presque	avant_6verres freqverrres.

PARTIE H. Vos activités en confinement

**H1. Portez-vous un masque quand vous êtes dehors (faire les courses par exemple) ?**<br>☐ Oui, tout le temps<br>☐ Oui, parfois<br>☐ Non, car je n'ai pas de masque mais sinon j'en porterai un<br>☐ Non, car je ne crois pas que ce soit important	masque_dehors masque.
**H2. <u>Au cours des 7 derniers jours</u>, avez-vous pratiqué les activités suivantes ?**	
Lecture : ☐ Non ☐ Oui mais pas tous les jours ☐ Oui tous les jours	act_lecture tsjours.
Jeux de société : ☐ Non ☐ Oui mais pas tous les jours ☐ Oui tous les jours	act_soc tsjours.
Jeux vidéo : ☐ Non ☐ Oui mais pas tous les jours ☐ Oui tous les jours	act_video tsjours.
Bricolage / Entretien de la maison : ☐ Non ☐ Oui mais pas tous les jours ☐ Oui tous les jours	act_brico tsjours.
Cuisine / Pâtisserie / Préparation de plats spéciaux : ☐ Non ☐ Oui mais pas tous les jours ☐ Oui tous les jours	act_cuis tsjours.
Ecriture / Dessin / Peinture : ☐ Non ☐ Oui mais pas tous les jours ☐ Oui tous les jours	act_ecrit tsjours.
Musique : ☐ Non ☐ Oui mais pas tous les jours ☐ Oui tous les jours	act_musiq tsjours.
Couture / Tricot / Broderie : ☐ Non ☐ Oui mais pas tous les jours ☐ Oui tous les jours	act_cout tsjours.
**H3. Actuellement, sur une échelle de 0 à 10 (0=pas du tout, 10=totalement), à quel point vous sentez-vous seul.e ?**<br>0 1 2 3 4 5 6 7 8 9 10	seul_eva likert.
**Q. Pendant cette période de confinement, à quelle fréquence avez-vous des interactions sociales avec votre famille ou des amis qui ne sont pas confinés avec vous ?**	

		Jamais	Moins d'une fois par semaine	Une fois par semaine	Plusieurs fois par semaine	Tous les jours	
H4.	**Directement en face à face**	❑	❑	❑	❑	❑	`interFreq_ftf freqsem5.`
H5.	**Au téléphone**	❑	❑	❑	❑	❑	`interFreq_tel freqsem5.`
H6.	**Par SMS**	❑	❑	❑	❑	❑	`interFreq_sms freqsem5.`
H7.	**Sur les réseaux sociaux**	❑	❑	❑	❑	❑	`interFreq_rs freqsem5.`

H8. Depuis le début du confinement, votre utilisation des réseaux sociaux a-t-elle changé ? ***#question conditionnelle#***

☐ Non
☐ Vous avez diminué votre utilisation
☐ Vous avez augmenté votre utilisation
☐ Je ne suis pas sur les réseaux sociaux

`reseaux_freq evolfreq.`

H9. Pensez-vous que cette modification d'utilisation des réseaux sociaux vous aide à mieux vivre le confinement ? ***#(seulement si modification = « diminué » ou « augmenté »)#***

☐ Oui, un peu
☐ Oui, beaucoup
☐ Non
☐ Je ne sais pas

`reseaux_aide ouibcp.`

PARTIE I. Vos perceptions sur la santé

Veuillez indiquer dans quelle mesure vous êtes d'accord ou non avec chacune des affirmations suivantes.

		Pas du tout d'accord	Pas d'accord	D'accord	Tout à fait d'accord	
**I1.**	Je compare les informations sur la santé qui viennent de différentes sources	❑	❑	❑	❑	infosante_compare accord.
**I2.**	Quand je découvre une nouvelle information sur la santé, je vérifie si elle est vraie ou non	❑	❑	❑	❑	infosante_verifie accord.
**I3.**	Je compare toujours les informations sur la santé à partir de différentes sources et je décide ce qui est le mieux pour moi	❑	❑	❑	❑	infosante_sources accord.
**I4.**	Je sais déterminer si une information sur la santé est adaptée à ma situation ou pas	❑	❑	❑	❑	infosante_determine accord.
**I5.**	J'interroge les professionnels de santé sur la qualité des informations que je trouve	❑	❑	❑	❑	infosante_pro accord.

PARTIE J. Vous gardez le moral ?

**J1. Comment jugez-vous actuellement votre moral sur une échelle de 0 à 10 ?**<br>*Donnez une note entre 0 (très mauvais) et 10 (très bon).*<br>0 1 2 3 4 5 6 7 8 9 10<br><br>**J2. À quel point êtes-vous inquiet(ète) ou stressé(e) en ce moment sur une échelle de 0 à 10 ?**<br>*Donnez une note entre 0 (no stress) et 10 (très stressé(e)).*<br>0 1 2 3 4 5 6 7 8 9 10	moral_act<br>likert.<br><br>stress_act<br>likert.
**J3. Avant le début du confinement, comment caractériseriez-vous votre sommeil ?**<br>❒ Bon<br>❒ Plutôt bon<br>❒ Ni bon ni mauvais<br>❒ Plutôt mauvais<br>❒ Mauvais<br><br>**J4. Au cours des 7 derniers jours, quelle a été votre qualité de sommeil ?**<br>☐ Beaucoup moins bien qu'avant<br>☐ Un peu moins bien qu'avant<br>☐ Ne s'est pas modifiée<br>☐ Un peu mieux qu'avant<br>☐ Beaucoup mieux qu'avant<br><br>**J5. Au cours des 7 derniers jours, quelle a été votre quantité de sommeil ?**<br>☐ Est plus courte de plus d'une heure<br>☐ Est plus courte de moins d'une heure<br>☐ Ne s'est pas modifiée<br>☐ Est plus longue de moins d'une heure<br>☐ Est plus longue de plus d'une heure<br><br>**J6. Au cours des 7 derniers jours, quelle a été votre capacité de rester éveillé.e tout au long de la journée ?**<br>☐ Beaucoup moins bien qu'avant<br>☐ Un peu moins bien qu'avant	som_avant<br>bienmal.<br><br>som_qualite<br>evolqual.<br><br>som_quantite<br>evolquant.

☐ Ne s’est pas modifiée<br>☐ Un peu mieux qu’avant<br>☐ Beaucoup mieux qu’avant	som_eveille evolqual.
**J7. Au cours des 7 derniers jours, votre heure de lever s’est-elle modifiée ?**<br>☐ Plus tôt de plus d’une heure<br>☐ Plus tôt de moins d’une heure<br>☐ Ne s’est pas modifiée<br>☐ Plus tard de moins d’une heure<br>☐ Plus tard de plus d’une heure	som_lever evolheure.
**J8. Au cours des 7 derniers jours, votre heure de coucher s’est-elle modifiée?**<br>☐ Plus tôt de plus d’une heure<br>☐ Plus tôt de moins d’une heure<br>☐ Ne s’est pas modifiée<br>☐ Plus tard de moins d’une heure<br>☐ Plus tard de plus d’une heure	som_coucher evolheure.
**J9. Au cours des 7 derniers jours, votre rythme de lever et de coucher s’est-il modifié?**<br>☐ Est beaucoup plus irrégulier<br>☐ Est plutôt plus irrégulier<br>☐ Ne s’est pas modifié<br>☐ Est plutôt plus régulier<br>☐ Est beaucoup plus régulier	som_rythem evolrythme.

Q. <u>Au cours des 7 derniers jours</u>, comment ont évolué vos consommations ?

		Je n'en consomme pas habituel-lement	Iden-tique	Diminution ou arrêt sans manque	Dimi-nution ou arrêt avec manque	Augmen-tation modérée	Augmen-tation difficile à contrôler
J10.	**Café, thé et/ou boissons énergisantes**	❑	❑	❑	❑	❑	❑
J11.	**Aliments gras, sucrés et/ou salés**	❑	❑	❑	❑	❑	❑
J12.	**Tabac**	❑	❑	❑	❑	❑	❑
J13.	**Cigarette électronique**	❑	❑	❑	❑	❑	❑
J14.	**Alcool**	❑	❑	❑	❑	❑	❑
J15.	**Cannabis**	❑	❑	❑	❑	❑	❑
J16.	**Autres drogues (ecstasy…)**	❑	❑	❑	❑	❑	❑
J17.	**Médicaments pour dormir**	❑	❑	❑	❑	❑	❑
J18.	**Anxiolytiques (alprazolam…)**	❑	❑	❑	❑	❑	❑
J19.	**Ecrans** (TV, smartphone, tablette, ordinateur…)	❑	❑	❑	❑	❑	❑

evolconso_cafe evolconso.
evolconso_sucre evolconso.
evolconso_tabac evolconso.
evolconso_ecig evolconso.
evolconso_alcool evolconso.
evolconso_cannabis evolconso.
evolconso_drogues evolconso.
evolconso_medic evolconso.
evolconso_anxio evolconso.
evolconso_ecran evolconso.

J20.#Si alcool au moins 6 verres <> Jamais, # **Si cela vous arrive de boire 6 verres d'alcool en une seule occasion et sur un temps court : cela vous arrive-t-il plus fréquemment depuis le confinement ?**

alcool_conso alcool.

☐ Non, la fréquence de ces épisodes n'a pas changé

☐ Oui, la fréquence a diminué/ j'ai arrêté

☐ Oui, la fréquence a un peu augmenté

☐ Oui, elle a un beaucoup augmenté

☐ *Je ne suis pas concerné(e)*

**J21.Si ces consommations précédentes ont évolué, pensez-vous que cela vous aide à mieux supporter le confinement ?** ***#(seulement si modification = « diminué » ou « augmenté ») J16 à J26#***<br>☐ Oui, un peu<br>☐ Oui, beaucoup<br>☐ Non	evolconso_aide ouibcp.
**J22. <u>Au cours des 7 derniers jours</u>, avez-vous eu plus fréquemment de graves disputes ou un climat de violence au sein de votre lieu de confinement ?**<br>☐ Non, la fréquence n'a pas changé<br>☐ Non, la fréquence a diminué<br>☐ Oui, la fréquence a un peu augmenté<br>☐ Oui, la fréquence a beaucoup augmenté<br>☐ *Je ne suis pas concerné(e)*<br><br>***#Si réponse « Mon conjoint » D14 proposer 4 questions suivantes***<br>**Q. Par rapport à la période avant le confinement, concernant les relations avec votre conjoint ou partenaire <u>au cours des 7 derniers jours</u> :**<br><br>**J23. Avec votre conjoint, avez-vous eu plus fréquemment des disputes ?**<br>☐ Non, la fréquence n'a pas changé<br>☐ Non, la fréquence a diminué<br>☐ Oui, la fréquence a un peu augmenté<br>☐ Oui, la fréquence a beaucoup augmenté<br>☐ *Je ne suis pas concerné(e)*<br><br>**J24. Avez-vous été plus fréquemment <u>victime</u> d'insultes ou de toute autre violence verbale ou psychologique ?**<br>☐ Non, la fréquence n'a pas changé<br>☐ Non, la fréquence a diminué<br>☐ Oui, la fréquence a un peu augmenté<br>☐ Oui, la fréquence a beaucoup augmenté<br>☐ *Je ne suis pas concerné(e)*<br><br>**J25.Avez-vous été plus fréquemment auteur d'insultes ou de toute autre violence verbale ou psychologique ?**<br>❐ Non, la fréquence n'a pas changé<br>❐ Non, la fréquence a diminué<br>❐ Oui, la fréquence a un peu augmenté	evoldispute evoldispute.<br><br>conjoint_dispute evoldispute.<br><br>conjoint_insulte evoldispute.<br><br>auteur_insulte evoldispute.

❒ Oui, la fréquence a beaucoup augmenté<br>❒ Je ne suis pas concerné.e<br><br>**J26. Avez-vous été plus fréquemment <u>victime</u> de menace ou d'agression physique ?**<br>☐ Non, la fréquence n'a pas changé<br>☐ Non, la fréquence a diminué<br>☐ Oui, la fréquence a un peu augmenté<br>☐ Oui, la fréquence a beaucoup augmenté<br>☐ *Je ne suis pas concerné(e)*<br><br>**J27.Avez-vous été plus fréquemment auteur de menace ou d'agression physique ?**<br>❒ Non, la fréquence n'a pas changé<br>❒ Non, la fréquence a diminué<br>❒ Oui, la fréquence a un peu augmenté<br>❒ Oui, la fréquence a beaucoup augmenté<br>❒ Je ne suis pas concerné.e	conjoint_menace evoldispute.<br><br>auteur_menace evoldispute.
**J28. Au cours des 7 derniers jours, vous est-il arrivé de penser à vous suicider (d'avoir des idées suicidaires) ?**<br>❒ Non, jamais<br>❒ Oui, quelquefois<br>❒ Oui, à de multiples reprises	suicide onqqf.
**J29. Quelle note donneriez-vous à votre qualité de vie <u>au cours des 7 derniers jours</u> ?**<br>*(0 pour la pire qualité de vie possible et 10 pour la meilleure qualité de vie possible)*<br>0 1 2 3 4 5 6 7 8 9 10<br><br>**J30. Toujours sur une échelle allant de 0 à 10, comment pensez-vous vous situer <u>après le confinement</u> ?**<br>*(0 pour la pire qualité de vie possible et 10 pour la meilleure qualité de vie possible)*<br>0 1 2 3 4 5 6 7 8 9 10<br><br>**J31.Diriez-vous que, malgré les difficultés, le confinement a comporté des éléments positifs pour vous-même au cours des 7 derniers jours ?**<br>❒ Oui<br>❒ Non	qdv_act likert.<br><br>qdv_apres likert.<br><br>positif ouinon.

**J32. Comment considérez-vous l'expérience du confinement (0=très négative, 10=très positive) ?**<br><br>0 1 2 3 4 5 6 7 8 9 10<br><br>**Exprimez-vous : Comment vivez-vous cette période de confinement (aspects négatifs et/ou positifs). Quel est votre ressenti ?**<br>*Nous vous proposons de vous exprimer librement en quelques mots ou quelques phrases.*	experience likert.<br><br>exprime_ressenti $3000.

QUESTIONNAIRE SUIVI HEBDOMADAIRE (période confinement)

**A1. <u>Au cours des 7 derniers jours</u>, pensez-vous avoir contracté le COVID-19 ?**<br>❒ Oui, j'ai été testé.e positif.ve<br>❒ Oui, cela a été évoqué par un médecin mais je n'ai pas été testé.e<br>❒ C'est possible, je présente des symptômes (fièvre, toux, courbatures, fatigue intense, diarrhées, perte de l'odorat, gêne respiratoire)<br>❒ C'est peu probable, je ne me sens pas malade<br>❒ Non, car j'ai déjà été malade du COVID-19<br>❒ Je ne sais pas	covid_atteint atteint7j.
**A2. <u>Au cours des 7 derniers jours</u>, des personnes vivant avec vous ont-elles eu le COVID-19 ou des signes de maladie laissant à penser que c'était le COVID-19 ?**<br>❒ Oui<br>❒ Non	covid_fam ouinon.
**A3. <u>Au cours des 7 derniers jours</u>, si vous avez étudié ou travaillé de chez vous, comment évaluez-vous votre capacité à effectuer les tâches qui ont été attendues de vous ?**<br>❒ J'étais loin de fournir le travail attendu et c'est un problème pour moi<br>❒ Je n'étais pas aussi performant/concentré que d'habitude mais j'ai réussi à travailler/étudier<br>❒ Je suis satisfait de ce que je suis arrivé à faire<br>❒ J'ai travaillé plutôt mieux que d'habitude<br>❒ Je ne suis pas concerné.e par cette question	eval_travail evaltr.
**A4. <u>Au cours des 7 derniers jours,</u> avez-vous pratiqué une activité physique <u>en intérieur</u> (gymnastique, vélo d'appartement) ?**<br>☐ Non<br>☐ Oui mais pas tous les jours<br>☐ Oui tous les jours	sport_int tsjours.
**<u>Au cours des 7 derniers jours</u>, avez-vous pratiqué une activité physique <u>en extérieur</u> (marche dehors,…) ?**<br>☐ Non<br>☐ Oui mais pas tous les jours<br>☐ Oui tous les jours	sport_ext tsjours.
**A5. <u>Au cours des 7 derniers jours</u>, quelle a été votre qualité de sommeil ?**	som_qualite evolqual.

- ❐ Beaucoup moins bonne qu'avant
- ❐ Un peu moins bonne qu'avant
- ❐ Ne s'est pas modifiée
- ❐ Un peu meilleur qu'avant
- ❐ Bien meilleur qu'avant

<u>Au cours des 7 derniers jours</u>, comment ont évolué vos consommations ?

		Je n'en consom-me pas habituel-lement	Iden-tique	Dimi-nution ou arrêt sans manque	Dimi-nution ou arrêt avec manque	Augmen-tation modérée	Augmen-tation difficile à contrôler	
**A6.**	**Café, thé et/ou boissons énergisantes**	❑	❑	❑	❑	❑	❑	evolconso_cafe evolconso.
**A7.**	**Aliments gras, sucrés et/ou salés**	❑	❑	❑	❑	❑	❑	evolconso_sucre evolconso.
**A8.**	**Tabac**	❑	❑	❑	❑	❑	❑	evolconso_tabac evolconso.
**A9.**	**Cigarette électronique**	❑	❑	❑	❑	❑	❑	evolconso_ecig evolconso.
**A10.**	**Alcool**	❑	❑	❑	❑	❑	❑	evolconso_alcool evolconso.
**A11.**	**Cannabis**	❑	❑	❑	❑	❑	❑	evolconso_cannabis evolconso.
**A12.**	**Autres drogues (ecstasy…)**	❑	❑	❑	❑	❑	❑	evolconso_drogues evolconso.
**A13.**	**Médicaments pour dormir**	❑	❑	❑	❑	❑	❑	evolconso_medic evolconso.
**A14.**	**Anxiolytiques (alprazolam…)**	❑	❑	❑	❑	❑	❑	evolconso_anxio evolconso.
**A15.**	**Ecrans** (TV, smartphone, tablette, ordinateur…)	❑	❑	❑	❑	❑	❑	evolconso_ecran evolconso.

A16. Comment jugez-vous actuellement votre moral sur une échelle de 0 à 10 ?

Donnez une note entre 0 (très mauvais) et 10 (très bon).

`moral_act likert.`

0 1 2 3 4 5 6 7 8 9 10<br><br>**A17.** **À quel point êtes-vous inquiet(ète) ou stressé(e) en ce moment sur une échelle de 0 à 10?**<br>*Donnez une note entre 0 (no stress) et 10 (très stressé(e)).*<br>0 1 2 3 4 5 6 7 8 9 10	stress_act likert.
**A18.** **<u>Au cours des 7 derniers jours</u>, avez-vous respecté les consignes de confinement telles que préconisées par les pouvoirs publics ?**<br>❒ Oui, strictement<br>❒ Oui, mais j'ai fait quelques entorses<br>❒ Non, j'essaye d'en tenir compte mais je ne les respecte pas vraiment<br>❒ Non, pas du tout	respect_cons consig.
**A19.** **<u>Au cours des 7 derniers jours</u>, avez-vous porté un masque quand vous étiez dehors (pour faire les courses par exemple) ?**<br>❒ Oui, tout le temps<br>❒ Oui, parfois<br>❒ Non, car je n'ai pas de masque mais sinon j'en porterais un<br>❒ Non, car je ne crois pas que ce soit important	masque_dehors masque.

<u>Durant les 7 derniers jours</u>, à quelle fréquence avez-vous été dérangé(e) par les problèmes suivants ?

		Presque jamais	Plusieurs jours	Plus de la moitié du temps	Presque tous les jours	
**A20.**	**Peu d'intérêt ou de plaisir à faire les choses**	❑	❑	❑	❑	pb_derang1 freq4.
**A21.**	**Se sentir triste, déprimé(e) ou désespéré(e)**	❑	❑	❑	❑	pb_derang2 freq4.
**A22.**	**Difficultés à s'endormir ou à rester endormi(e), ou trop dormir**	❑	❑	❑	❑	pb_derang3 freq4.
**A23.**	**Se sentir fatigué(e) ou avoir peu d'énergie**	❑	❑	❑	❑	pb_derang4 freq4.

A24.	Peu d'appétit ou trop manger	❑	❑	❑	❑	pb_derang5 freq4.
A25.	Mauvaise perception de vous-même — ou vous pensez que vous êtes un perdant ou que vous n'avez pas satisfait vos propres attentes ou celles de votre famille	❑	❑	❑	❑	pb_derang6 freq4.
A26.	Difficultés à se concentrer sur des choses telles que lire le journal ou regarder la télévision	❑	❑	❑	❑	pb_derang7 freq4.
A27.	Vous bougez ou parlez si lentement que les autres personnes ont pu le remarquer. Ou au contraire – vous êtes si agité(e) que vous bougez beaucoup plus que d'habitude	❑	❑	❑	❑	pb_derang8 freq4.
A28.	Vous avez pensé que vous seriez mieux mort(e) ou Vous avez pensé à vous blesser d'une façon ou d'une autre	❑	❑	❑	❑	pb_derang9 freq4.

<u>Durant les 7 derniers jours</u>, à quelle fréquence avez-vous été gêné(e) par les problèmes suivants ? Cochez la case appropriée.

		Presque jamais	Plusieurs jours	Plus de la moitié du temps	Presque tous les jours	
A29.	Un sentiment de nervosité, d'anxiété ou de tension	❑	❑	❑	❑	pb_gene1 freq4.
A30.	Une incapacité à arrêter de s'inquiéter ou à contrôler ses	❑	❑	❑	❑	pb_gene2 freq4.

	inquiétudes					
**A31.**	**Une inquiétude excessive à propos de différentes choses**	❑	❑	❑	❑	pb_gene3 freq4.
**A32.**	**Des difficultés à me détendre**	❑	❑	❑	❑	pb_gene4 freq4.
**A33.**	**Une agitation telle qu'il m'est difficile de tenir en place**	❑	❑	❑	❑	pb_gene5 freq4.
**A34.**	**Une tendance à être facilement contrarié(e) ou irritable**	❑	❑	❑	❑	pb_gene6 freq4.
**A35.**	**Un sentiment de peur comme si quelque chose de terrible risquait de se produire**	❑	❑	❑	❑	pb_gene7 freq4.

A36. **<u>Au cours des 7 derniers jours</u>, vous est-il arrivé de penser à vous suicider (d'avoir des idées suicidaires) ?**

- ❒ Non, jamais
- ❒ Oui, quelquefois
- ❒ Oui, à de multiples reprises

suicide onqqf.

A37. **Quelle note donneriez-vous à votre qualité de vie <u>au cours des 7 derniers jours</u> ?**
(0 pour la pire qualité de vie possible et 10 pour la meilleure qualité de vie possible)

0 1 2 3 4 5 6 7 8 9 10

qdv_act likert.

A38. **Diriez-vous que, malgré les difficultés, le confinement a comporté des éléments positifs pour vous-même <u>au cours des 7 derniers jours</u> ?**

- ❒ Oui
- ❒ Non

positif ouinon.

Exprimez-vous : Comment avez-vous vécu cette nouvelle semaine de

**confinement (aspects négatifs et/ou positifs). Quel est votre ressenti ?**<br>*Nous vous proposons de vous exprimer librement en quelques mots ou quelques phrases.*	exprime_semaine<br>$3000.

QUESTIONNAIRE INCLUSION - Première partie (période post confinement)

Critères d'éligibilité

**Q1. Date de naissance :** |__|__|/ |__|__|__|__| (≥ 18 ans)<br>**Pays de naissance** : ☐ France ☐ Autre<br>*# Si France, #* Précisez la commune<br>|__|__|__|__|__| (code postal)<br><br>**Q2. Etes vous étudiant ?**<br>☐ Oui ☐ Non<br><br>*Patient éligible si :*<br>• *Age ≥ 18 ans*<br>•	naiss_date MMYY10.<br>naiss_pays pays.<br>naiss_commune 5.<br><br>etudiant ouinon.

PARTIE A. Vos données sociodémographiques

(page 1)

**A1. Sexe :** ☐ Masculin ☐ Féminin ☐ Autre	sexe sexe.
**A2. Taille : |__|__|__|cm** **Poids : |__|__|__|kg**	taille 3.<br>poids 3.
**A3. Votre situation de famille**<br>☐ Célibataire<br>☐ En couple (depuis au moins 3 mois) sans être marié.e ni pacsé.e<br>☐ Marié.e, Pacsé.e<br>☐ Autre (divorcé.e, veuf.ve)	situation_fam sitfam.
**A4. Avez-vous des enfants ?**<br>☐ Oui ☐ Non<br># Si oui, # combien : |__|__|	etude_enfant ouinon.<br>enfant_nb 2.
**A5. Quel est votre domaine d'études ?**<br>☐ Sciences Humaines et Sociales, Lettres, Langues, Art<br>☐ Sciences, Technique, Ingénierie, Informatique<br>☐ Droit, Economie, Gestion, Finance, Sciences Politiques,	etude_domaine etdom.

Comptabilité<br>☐ Médecine<br>☐ Autres études en Santé<br>☐ Autre<br><br>**A6. Quelle est votre filière d'études ?**<br>***#menu déroulant#***<br>41. Administration économique et sociale<br>42. Administration publique<br>43. Agronomie, agroalimentaire<br>44. Architecture, design<br>45. Arts (plastiques, du spectacle)<br>46. Commerce, vente<br>47. Droit<br>48. Economie et gestion<br>49. Electronique, énergie électrique, automatique<br>50. Génie civil<br>51. Géographie et aménagement<br>52. Histoire<br>53. Histoire de l'art et archéologie<br>54. Hôtellerie, restauration, tourisme<br>55. Information-communication<br>56. Informatique<br>57. Lettres, langues et civilisations étrangères et régionales<br>58. Mathématiques<br>59. Mathématiques et informatique appliquées aux sciences humaines et sociales<br>60. Mécanique<br>61. Médecine<br>62. Philosophie<br>63. Physique, chimie<br>64. Psychologie<br>65. Science politique<br>66. Sciences cognitives<br>67. Sciences de l'environnement<br>68. Sciences de la vie et de la Terre<br>69. Sciences de l'éducation<br>70. Sciences de l'homme, anthropologie, ethnologie<br>71. Sciences des matériaux<br>72. Sciences du langage<br>73. Sciences et techniques des activités physiques et sportives<br>74. Sciences et technologies<br>75. Sciences pour la santé autres que médecine<br>76. Sciences pour l'ingénieur<br>77. Sciences sanitaires et sociales<br>78. Sciences sociales<br>79. Télécommunications<br>80. Autre	`etude_filiere filet.`
Demander une seule fois par personne (étudiant	

uniquement) :<br><br>**Dans quel type d'établissement de l'enseignement supérieur êtes-vous inscrit ?**<br>❒ Université<br>❒ Autre établissement<br>*#Si université#* **Région d'étude :**<br>*#menu déroulant#*<br>Auvergne-Rhône-Alpes<br>Bourgogne-Franche-Comté<br>Bretagne<br>Centre-Val de Loire<br>Collectivités d'outre-mer<br>Corse<br>Grand Est<br>Guadeloupe<br>Guyane<br>Hauts-de-France<br>Île-de-France<br>La Réunion<br>Mayotte<br>Normandie<br>Nouvelle-Aquitaine<br>Occitanie<br>Pays de la Loire<br>Provence-Alpes-Côte d'Azur<br>*#affichage des universités en fonction de la région choisie#*<br>*#menu déroulant#* + « Autre »<br>*#si autre#* **Préciser : ……………………………………..**<br><br>*#Si autre établissement#* **Préciser : ……………………………………..**	univ_type untype.<br><br>univ_region univreg.<br><br>universite univ.<br><br>universite_txt $255.<br><br>univ_autre $255.
**A7. En quelle année d'études êtes-vous ?**<br>☐ 1ère ☐ 2ème ☐ 3ème ☐ 4ème ☐ 5ème ☐ > 5ème<br><br>**A8. Participez-vous à l'étude i-Share ?**<br>❒ Oui<br>❒ Non<br><br>**A9. Concernant la situation économique de la famille qui vous a élevé(e) durant votre enfance et adolescence diriez-vous qu'elle**	etude_an annee.<br><br>Ishare ouinon.

**était :**<br><br>☐ Très confortable<br>☐ Confortable<br>☐ Correcte<br>☐ Difficile<br>☐ Très difficile<br><br>**A10. Quelle est votre principale source de revenus pour le financement de votre année universitaire ? *(Une seule réponse possible)***<br><br>☐ Famille<br>☐ Bourse(s)<br>☐ Activités rémunérées<br>☐ Autre (économies, épargnes, prêt étudiant)<br><br>**A11. A combien estimez-vous vos ressources financières mensuelles tout compris (participation des parents, bourses, aides étudiants étrangers, travail, épargne…) ?**<br><br>☐ Moins de 500 euros<br>☐ Entre 500 et 700 euros<br>☐ Entre 700 et 1000 euros<br>☐ Plus de 1000 euros	enfance_siteco siteco.<br><br>etude_revenu revenu.<br><br>etude_reveuros reveuros.
**A12. Quel est votre diplôme le plus élevé ?**<br><br>☐ Aucun<br>☐ BEPC (Brevet des collèges), BEP, CAP, BP (Brevet professionnel)<br>☐ Baccalauréat (général, technologique ou professionnel)<br>☐ Bac+2 (DEUG, BTS, DUT ou équivalent)<br>☐ Bac+3 à Bac+4 (Licence, Master 1, DEI ou équivalent)<br>☐ Bac+5 ou plus (Master 2, école d'ingénieur, doctorat ou équivalent)<br><br>**A16. Quand vous étiez étudiant, avez-vous participé à l'étude i-Share ?**<br><br>☐ Oui ☐ Non<br><br>**A13. Quelle est votre profession ?**<br><br>☐ Agriculteur.trice (ex. éleveur.se, viticulteur.trice)<br>☐ Artisan, commerçant.e, chef.fe d'entreprise (ex. électricien.ne à son compte, gérant.e)<br>☐ Profession intermédiaire (ex. professeur.e des écoles,	niveau_etude nivet.<br><br>nonet_ishare ouinon.<br><br>profession prof.

technicien.ne, infirmièr.e)<br>☐ Cadre, ingénieur.e, profession libérale (ex. médecin, journaliste)<br>☐ Employé.e administratif d'entreprise ou de la fonction publique (ex. secrétaire, hôtesse de l'air/stewart, policier.e, aide-soignant.e)<br>☐ Employé.e de commerce (ex. caissièr.e, vendeur.se en magasin, pompiste)<br>☐ Personnel des services directs aux particuliers (ex. serveur.se, assistant.e maternelle)<br>☐ Ouvrier.e (ex. opérateur.e, chauffeur.e, peintre en bâtiment, magasinier.e)<br>☐ Vous n'avez pas de profession	
# Si profession cochée (autre que « pas de profession ») #<br>Votre profession est-elle en rapport avec le domaine de la santé ?<br>☐ Oui ☐ Non	prof_sante ouinon.
# Si oui, # Etes-vous soignant au contact de malades ?<br>☐ Oui ☐ Non	prof_soigne ouinon.
**A14. Quelle était votre situation professionnelle <u>avant l'épidémie ?</u>**<br>☐ En CDI ou fonctionnaire<br>☐ En CDD ou autre contrat court (saisonnier, vacataire, intérim, pigiste, CESU)<br>☐ Indépendant (y compris auto-entrepreneur, en freelance, libéral)<br>☐ Stagiaire, volontaire ou apprenti.e<br>☐ Vous avez un petit boulot non déclaré (ex. babysitting)<br>☐ Vous avez un autre type de contrat de travail<br>☐ Sans travail et à la recherche d'un emploi<br>☐ Au foyer<br>☐ Vous ne travaillez pas actuellement et vous ne cherchez pas d'emploi<br>☐ Retraité	situation_pro sitpro.
**A15. Quel est habituellement le montant des revenus mensuels de votre ménage (c'est-à-dire vous-même et votre conjoint.e si vous habitez avec quelqu'un) en incluant toutes les sources de revenus : salaires + allocations + pensions etc. ?**<br>☐ 500 euros ou moins	

☐ De 501 à 1700 euros<br>☐ De 1701 à 2500 euros<br>☐ De 2501 à 4000 euros<br>☐ De 4001 à 7000 euros<br>☐ Plus de 7000 euros<br>☐ Ne souhaite pas répondre	pro_revenu prorevenu.

PARTIE B. Vos perceptions sur l'épidémie en cours

(page 2/3)

**B1. Pensez-vous ou savez-vous si vous êtes actuellement atteint(e) par le COVID-19 ?**<br>☐ Oui, j'ai été testé(e) positif<br>☐ Oui, cela a été évoqué par un médecin mais je n'ai pas été testé ou j'ai été testé négatif<br>☐ C'est possible, je présente des symptômes (fièvre, toux, courbatures, fatigue intense, diarrhées, douleur thoracique, perte de l'odorat, gêne respiratoire)<br>☐ C'est peu probable, je ne me sens pas malade<br>☐ Je suis certain(e) de ne pas être atteint(e)<br>☐ Je ne sais pas	covid_atteint atteint.
#si oui#<br>**B2. Pensez-vous avoir contracté le COVID-19 au cours de votre exercice professionnel ?**<br>☐ Oui, j'en suis certain.e<br>☐ Oui, c'est possible<br>☐ Non, je ne pense pas<br>☐ Je ne sais pas	covid_atteint_pro attpro.
**B3. Avez-vous été hospitalisé.e pour la prise en charge du COVID-19 ?**<br>☐ Oui ☐ Non	covid_atteint_hosp ouinon.
**B4. Avez-vous déjà eu le COVID-19 et êtes-vous actuellement rétabli.e ?**<br>☐ Oui avec un diagnostic confirmé (test positif)<br>☐ Oui mais sans qu'un test ait été réalisé<br>☐ Non	covid_sympt ontest.
**B5. Y a-t-il, dans votre entourage ou votre famille, des personnes qui ont eu le COVID-19 ou des signes de maladie laissant à penser que c'était le COVID-19 ?**<br>☐ Oui ☐ Non	covid_fam ouinon.

#si oui#<br><br>**B6. Un de vos proches a-t-il été hospitalisé pour la prise en charge du COVID-19 ?**<br><br>☐ Oui ☐ Non<br><br>**B7. Un de vos proches est-il décédé des suites du COVID-19 ?**<br><br>☐ Oui ☐ Non	covid_fam_hosp ouinon.<br><br>covid_fam_deces ouinon.

B8. Dans le contexte de cette épidémie, quel est votre degré d'inquiétude à propos de :

	1 Pas du tout inquiet(ète)	2	3	4	5 Très inquiet(ète)	Non concerné(e)	
Votre santé (infection par le COVID-19)	❑	❑	❑	❑	❑	❑	inq_sante inquiet.
La santé de vos parents	❑	❑	❑	❑	❑	❑	inq_parent inquiet.
La santé de vos proches, de vos amis	❑	❑	❑	❑	❑	❑	inq_amis inquiet.
Votre situation financière	❑	❑	❑	❑	❑	❑	inq_finance inquiet.
De manquer de quelque chose (par ex., médicaments, pain, …)	❑	❑	❑	❑	❑	❑	inq_manque inquiet.
De manquer de soutien moral et affectif	❑	❑	❑	❑	❑	❑	inq_soutien inquiet.

B9. Vos revenus ont-ils changé avec l'épidémie ?

❑ Oui, ils ont diminué (perte de travail, de petit boulot, d'aides, de bourse, etc.)

❑ Non, ils sont restés inchangés

revenu_change change.

**B10.** *# Si profession cochée (autre que « pas de profession ») #* **Indiquez votre degré d'inquiétude par rapport à votre emploi (fin de CDD, licenciement,…) :**<br>*Donnez une note entre 0 (pas du tout inquiet(ète)) et 10 (très inquiet(ète)).*<br><br>0 1 2 3 4 5 6 7 8 9 10	inq_travail likert.
**B11. Indiquez votre degré d'inquiétude par rapport à vos études (report des examens, validation de l'année, report de la rentrée, report des soutenances…) :**<br>*Donnez une note entre 0 (pas du tout inquiet(ète)) et 10 (très inquiet(ète)).*<br><br>0 1 2 3 4 5 6 7 8 9 10	inq_etude likert.
**B12. A quel degré situez-vous la dangerosité du COVID-19 pour vous-même ou vos proches ?**<br>*Donnez une note entre 0 (pas dangereux) et 10 (très dangereux).*<br><br>0 1 2 3 4 5 6 7 8 9 10	inq_danger likert.
**B13. Que pensez-vous de l'évolution de l'épidémie ? (Plusieurs réponses possibles)**<br>☐ L'épidémie va encore durer quelques semaines puis tout va rentrer dans l'ordre et nous reprendrons nos vies comme avant<br>☐ L'épidémie va évoluer par cycles pendant plusieurs mois et il pourra y avoir des phases de reconfinement<br>☐ Le coronavirus va rester parmi nous pendant plusieurs années et il faudra continuer à faire attention (gestes barrières, distanciation sociale)<br>☐ Sans opinion	epidemie_evol1 coch.<br>epidemie_evol6 coch.<br>epidemie_evol7 coch.<br>epidemie_evol5 coch.
**Q. Selon vous, les affirmations suivantes sont-elles vraies ou fausses ?**	
**B14.** Le virus du COVID-19 a été conçu par l'Institut Pasteur en 2004<br>☐ Vrai ☐ Faux ☐ Je ne sais pas	fakenews9 vraifaux.
**B15.** Le virus est propagé par la 5G<br>☐ Vrai ☐ Faux ☐ Je ne sais pas	fakenews10 vraifaux.
**B16.** Ce sont presque toujours les personnes âgées de plus de 70 ans qui	fakenews11

décèdent du COVID-19<br><br>☐ Vrai ☐ Faux ☐ Je ne sais pas<br><br>**B17.** Les enfants et adultes jeunes (moins de 25 ans) font rarement des formes sévères du COVID-19<br><br>☐ Vrai ☐ Faux ☐ Je ne sais pas<br><br>**B18.** Toutes les personnes contaminées par le Covid-19 développent des symptômes<br><br>☐ Vrai ☐ Faux ☐ Je ne sais pas<br><br>**B19.** Boire des boissons très chaudes ou prendre des bains chauds permet d'éviter la contamination par le virus<br><br>☐ Vrai ☐ Faux ☐ Je ne sais pas<br><br>**B20.** Le virus ne peut être transmis que par des personnes qui ont des symptômes comme de la fièvre et de la toux<br><br>☐ Vrai ☐ Faux ☐ Je ne sais pas<br><br>**B21.** Le virus peut survivre un certain temps sur des surfaces inertes comme les poignées de porte ou les barres dans les transports en commun<br><br>☐ Vrai ☐ Faux ☐ Je ne sais pas	vraifaux.<br><br>fakenews12<br>vraifaux.<br><br>fakenews6<br>vraifaux.<br><br>fakenews8<br>vraifaux.<br><br>fakenews5<br>vraifaux.<br><br>fakenews3<br>vraifaux.

PARTIE C. Les consignes : les suivez-vous ? Qu'en pensez-vous ?

(page 4)

C17. Si vous n'êtes pas obligé d'aller sur votre lieu de travail, respectez-vous strictement les consignes de confinement telles que préconisées par l'état ?

1<br>Pas du tout	2	3	4	5<br>Tout à fait	Non concerné(e)
❑	❑	❑	❑	❑	❑

respect_cons1
ech5.

C18. Quand vous faites des courses ou sur votre lieu de travail, gardez-vous une distance d'au moins un mètre entre vous et d'autres personnes ?

1<br>Pas du tout	2	3	4	5<br>Tout à fait	Non concerné(e)
❑	❑	❑	❑	❑	❑

respect_cons2
ech5.

C19. Vous lavez-vous plus fréquemment les mains que d'habitude ?

1<br>Pas du tout	2	3	4	5<br>Tout à fait	Non concerné(e)
❑	❑	❑	❑	❑	❑

respect_cons3
ech5.

C20. Diriez-vous que la réaction du gouvernement face à l'épidémie actuelle est :

- ☐ Trop exagérée
- ☐ Plutôt exagérée
- ☐ Appropriée
- ☐ Plutôt insuffisante
- ☐ Bien trop insuffisante

reaction_gouv
reac.

C21. Diriez-vous que la réaction de la population face à l'épidémie actuelle est :

- ☐ Trop exagérée
- ☐ Plutôt exagérée
- ☐ Appropriée
- ☐ Plutôt insuffisante
- ☐ Bien trop insuffisante

reaction_pop
reac.

**C22. Trouvez-vous, à titre personnel, que les mesures sanitaires (distanciation physique, port de masque,…) sont faciles à respecter ?**<br>☐ Oui, tout à fait<br>☐ Oui, plutôt<br>☐ Non, plutôt pas<br>☐ Non, pas du tout	mesure_facil ech4.
**C23. Faites-vous confiance aux pouvoirs publics pour contrôler l'épidémie de COVID-19 ?**<br>*Donnez une note entre 0 (pas du tout confiance) et 10 (tout à fait confiance).*<br>0 1 2 3 4 5 6 7 8 9 10	conf_controle likert.
**C24. Faites-vous confiance aux pouvoirs publics pour vous informer sur le COVID-19 ?**<br>*Donnez une note entre 0 (pas du tout confiance) et 10 (tout à fait confiance).*<br>0 1 2 3 4 5 6 7 8 9 10	conf_info likert.
**Exprimez-vous : Qu'est-ce que vous pensez des mesures prises par les autorités publiques concernant l'épidémie ?**<br>*Nous vous proposons de vous exprimer librement en quelques mots ou quelques phrases.*	exprime_autorite $3000.

PARTIE D. Votre situation actuelle

(Page 5/6)

**D1. Disposez-vous actuellement d'une connexion Internet ?**<br>☐ Oui ☐ Non<br><br># Si oui, # est-elle suffisamment performante :<br>**D2. Dans le cadre de votre travail ou de vos études ?** ☐ Oui ☐ Non ☐ Non concerné(e)<br>**D3. Afin de maintenir un contact visuel (type Skype) avec vos proches ?** ☐ Oui ☐ Non ☐ Non concerné(e)<br>**D4. Pour vous divertir (téléchargement séries ou musique, streaming) ?** ☐ Oui ☐ Non ☐ Non concerné(e)	conf_internet ouinon.<br><br>conf_int_teletr onnc.<br><br>conf_int_contact onnc.<br><br>conf_int_loisir onnc.
**D5. Quelle est votre situation professionnelle actuelle :**<br>☐ Vous travaillez comme auparavant sur votre lieu de travail à temps complet<br>☐ Vous télétravaillez à temps complet<br>☐ Vous avez une activité mixte entre télétravail et présence sur votre lieu de travail<br>☐ Vous avez diminué votre temps de travail ou ne travaillez que sur certains lieux<br>☐ Vous êtes au chômage technique ou partiel<br>☐ Vous êtes en congé forcé<br>☐ Vous êtes en arrêt de travail pour garde d'enfants<br>☐ Vous êtes en arrêt de travail pour maladie<br>☐ Vous avez perdu votre emploi du fait de la crise sanitaire<br>☐ Autre<br><br>**D6. Si vous devez travailler ou étudier de chez vous, comment évaluez-vous votre capacité à effectuer les tâches qui sont attendues de vous dans votre environnement actuel ?**<br>☐ Je suis loin de fournir le travail attendu et c'est un problème pour moi<br>☐ Je ne suis pas aussi performant(e)/concentré(e) que d'habitude mais j'arrive à travailler<br>☐ Je suis satisfait(e) de ce que j'arrive à faire<br>☐ Je travaille plutôt mieux que d'habitude<br>☐ *Je ne suis pas concerné(e) par cette question*	situation_act sitact.<br><br>eval_travail evaltr.

**D7. <u>Au cours des 7 derniers jours</u>, avez-vous pratiqué une activité physique <u>en intérieur</u> (gymnastique, vélo d'appartement) ?**<br>☐ Non<br>☐ Oui mais pas tous les jours<br>☐ Oui tous les jours	sport_int tsjours.
**<u>Au cours des 7 derniers jours</u>, avez-vous pratiqué une activité physique <u>en extérieur</u> (marche, footing…) ?**<br>☐ Non<br>☐ Oui mais pas tous les jours<br>☐ Oui tous les jours	sport_ext tsjours.

PARTIE E. Impact de l'épidémie

(page 7/8)

Q. Au cours des 2 dernières semaines, à quelle fréquence avez-vous été dérangé(e) par les problèmes suivants ?

		Presque jamais	Plusieurs jours	Plus de la moitié du temps	Presque tous les jours	
E1.	Peu d'intérêt ou de plaisir à faire les choses	❑	❑	❑	❑	pb_derang1 freq4.
E2.	Se sentir triste, déprimé(e) ou désespéré(e)	❑	❑	❑	❑	pb_derang2 freq4.
E3.	Difficultés à s'endormir ou à rester endormi(e), ou trop dormir	❑	❑	❑	❑	pb_derang3 freq4.
E4.	Se sentir fatigué(e) ou avoir peu d'énergie	❑	❑	❑	❑	pb_derang4 freq4.
E5.	Peu d'appétit ou trop manger	❑	❑	❑	❑	pb_derang5 freq4.
E6.	Mauvaise perception de vous-même — ou vous pensez que vous êtes un perdant ou que vous n'avez pas satisfait vos propres attentes ou celles de	❑	❑	❑	❑	pb_derang6 freq4.

	votre famille					
**E7.**	Difficultés à se concentrer sur des choses telles que lire le journal ou regarder la télévision	❑	❑	❑	❑	pb_derang7 freq4.
**E8.**	Vous bougez ou parlez si lentement que les autres personnes ont pu le remarquer. Ou au contraire – vous êtes si agité(e) que vous bougez beaucoup plus que d'habitude	❑	❑	❑	❑	pb_derang8 freq4.
**E9.**	Vous avez pensé que vous seriez mieux mort(e) ou vous avez pensé à vous blesser d'une façon ou d'une autre	❑	❑	❑	❑	pb_derang9 freq4.

Q. Au cours des 2 dernières semaines, à quelle fréquence avez-vous été gêné(e) par les problèmes suivants ?

		Presque jamais	Plusieurs jours	Plus de la moitié du temps	Presque tous les jours	
**E10.**	Un sentiment de nervosité, d'anxiété ou de tension	❑	❑	❑	❑	pb_gene1 freq4.
**E11.**	Une incapacité à arrêter de s'inquiéter ou à contrôler ses inquiétudes	❑	❑	❑	❑	pb_gene2 freq4.
**E12.**	Une inquiétude excessive à propos de différentes choses	❑	❑	❑	❑	pb_gene3 freq4.
**E13.**	Des difficultés à me détendre	❑	❑	❑	❑	pb_gene4 freq4.
**E14.**	Une agitation telle qu'il m'est difficile de tenir en place	❑	❑	❑	❑	pb_gene5 freq4.
**E15.**	Une tendance à être facilement contrarié(e) ou irritable	❑	❑	❑	❑	pb_gene6 freq4.

E16.	Un sentiment de peur comme si quelque chose de terrible risquait de se produire	❑	❑	❑	❑	pb_gene7 freq4.

PARTIE F. Votre avis sur traitements et vaccins

(page 9)

**F1. Si vous étiez atteint.e du COVID-19, seriez-vous prêt.e à prendre un traitement dont l'efficacité n'est pas certaine ?**<br>☐ Oui, même s'il y a un risque à prendre ce traitement<br>☐ Oui, mais à condition que les risques liés au traitement soient faibles<br>☐ Oui, mais à condition qu'il n'y ait absolument aucun risque lié au traitement, même faible<br>☐ Non, jamais<br>☐ Je ne sais pas	trait_prendre<br>prendre.
**F2. Pensez-vous que les traitements de médecine alternative ou homéopathique sont efficaces contre le COVID-19 ?**<br>☐ Oui ☐ Non ☐ Je ne sais pas	trait_homeo<br>ouinon.
**F3. Avez-vous confiance dans le fait qu'un traitement efficace contre le COVID-19 sera trouvé ?**<br>☐ Oui, dans quelques mois<br>☐ Oui, dans quelques années<br>☐ Non<br>☐ Je ne sais pas	trait_covid<br>trait.
**F4. Seriez-vous prêt.e à vous faire vacciner contre le COVID-19 même si le vaccin n'a pas encore fait complètement la preuve de son efficacité ?**<br>☐ Oui, même s'il y a un risque lié au vaccin<br>☐ Oui, mais à condition que le risque lié au vaccin soit faible<br>☐ Oui, mais à condition qu'il n'y ait absolument aucun risque lié au vaccin, même faible<br>☐ Oui, dans le cadre d'un essai clinique (si un vaccin n'est pas encore validé)<br>☐ Non, jamais<br>☐ Je ne sais pas	vaccovid<br>vaccovid.
**F5. Si un vaccin contre le COVID-19 était produit dans 12 mois, alors que**	vaccovid_an

l'épidémie actuelle serait passée mais qu'il y aurait un risque qu'elle revienne chaque année comme la grippe, iriez-vous vous faire vacciner ?<br><br>1 Pas du tout ❑ · 2 ❑ · 3 ❑ · 4 ❑ · 5 Certainement ❑	cert.
**F6.** **Vous faites-vous vacciner régulièrement contre la grippe ?**<br>☐ Oui ☐ Non<br><br>**F7.** **Etes-vous à jour de vos vaccinations ?**<br>☐ Oui ☐ Non ☐ Je ne sais pas	vacgrippe ouinon<br><br>vacautre onnsp.
**Exprimez-vous : Que pensez-vous de la vaccination comme mesure pour prévenir les maladies comme le COVID-19 ?**<br>*Nous vous proposons de vous exprimer librement en quelques mots ou quelques phrases.*	exprime_vaccin $3000.

PARTIE G. Votre profil médical

(page 10/11)

**G1.** **Avant le début de l'épidémie, comment caractériseriez-vous votre santé ?**<br>❒ Très bonne<br>❒ Bonne<br>❒ Moyenne<br>❒ Mauvaise<br>❒ Très mauvaise	sante_avant evalsante.
**G2.** **Avant le début de l'épidémie, quelle note donneriez-vous à votre qualité de vie ?**<br>*(0 pour la pire qualité de vie possible et 10 pour la meilleure qualité de vie possible)*	qdv_avant likert.

0 1 2 3 4 5 6 7 8 9 10<br><br>**G3. Avant le début de l'épidémie, diriez-vous que vous êtiez souvent inquiet.ète ou stressé.e ?**<br>*Donnez une note entre 0 (no stress) et 10 (très stressé(e)).*<br>0 1 2 3 4 5 6 7 8 9 10<br><br>**G4. Tendez-vous à rebondir rapidement après des moments difficiles ?**<br>☐ Oui, tout à fait<br>☐ Plutôt oui<br>☐ Neutre<br>☐ Non, pas vraiment<br>☐ Non, pas du tout	stress_normal likert.<br><br>rebondir onneutre.
**G5. Au cours de votre vie, un médecin a-t-il déjà diagnostiqué chez vous un ou plusieurs problèmes de santé suivants ?**<br>Maladie cardio-vasculaire (infarctus du myocarde, AVC,…)<br>☐ Oui ☐ Non ☐ Ne sait pas<br>*# Si oui, #* êtes-vous actuellement traité ? ☐ Oui ☐ Non<br><br>Hypertension artérielle<br>☐ Oui ☐ Non ☐ Ne sait pas<br>*# Si oui, #* êtes-vous actuellement traité ? ☐ Oui ☐ Non<br><br>Diabète<br>☐ Oui ☐ Non ☐ Ne sait pas<br>*# Si oui, #* êtes-vous actuellement traité ? ☐ Oui ☐ Non<br><br>Maladie digestive chronique (ex. Crohn, ulcère)<br>☐ Oui ☐ Non ☐ Ne sait pas<br>*# Si oui, #* êtes-vous actuellement traité ? ☐ Oui ☐ Non<br><br>Cancer<br>☐ Oui ☐ Non ☐ Ne sait pas	atcd_cardio onnsp.<br>atcd_cardio_tt ouinon.<br><br>atcd_hta onnsp.<br>atcd_hta_tt ouinon.<br><br>atcd_diab onnsp.<br>atcd_diab_tt ouinon.<br><br>atcd_digest onnsp.<br>atcd_digest_tt ouinon.<br><br>atcd_cancer onnsp.<br>atcd_cancer_tt ouinon.

# Si oui, # êtes-vous actuellement traité ? ☐ Oui ☐ Non<br><br>Asthme ou autre problème respiratoire<br>☐ Oui ☐ Non ☐ Ne sait pas<br>*# Si oui, #* êtes-vous actuellement traité ? ☐ Oui ☐ Non	atcd_respi onnsp.<br>atcd_respi_tt ouinon.
Un problème de santé mentale (dépression, trouble bipolaire, anxiété généralisée…)<br>☐ Oui ☐ Non ☐ Ne sait pas<br>*# Si oui, #* êtes-vous actuellement traité ? ☐ Oui ☐ Non	atcd_psy onnsp.<br>atcd_psy_tt ouinon.
Autre(s) maladie affectant le système immunitaire (comme HIV, lymphopathie maligne, …)<br>☐ Oui ☐ Non ☐ Ne sait pas<br>*# Si oui, #* êtes-vous actuellement traité ? ☐ Oui ☐ Non	atcd_immun onnsp.<br>atcd_immun_tt ouinon.
Autre(s) problème(s) de santé<br>☐ Oui ☐ Non ☐ Ne sait pas<br>*# Si oui, #* êtes-vous actuellement traité ? ☐ Oui ☐ Non	atcd_autre onnsp.<br>atcd_autre_tt ouinon.
**Les questions suivantes concernent votre consommation de tabac et d'alcool <u>au cours de l'année écoulée</u> et pas seulement les dernières semaines.**<br>**G6. Concernant votre consommation de tabac au cours de l'année écoulée, êtes-vous :**<br>☐ Fumeur.se régulier.e de tabac (au moins une cigarette par jour)<br>☐ Fumeur.se occasionnel.le de tabac (moins d'une cigarette par jour)<br>☐ Ex-fumeur.se régulier.e de tabac (au moins une cigarette par jour)<br>☐ Non-fumeur.se (vous n'avez jamais fumé une cigarette par jour)	avant_tabac fumeur.
**G7. Au cours de l'année écoulée, avez-vous consommé du cannabis ?**<br>☐ Non<br>☐ Oui, ponctuellement<br>☐ Oui, régulièrement mais moins de 4 fois par semaine<br>☐ Oui, au moins 4 fois par semaine<br>☐ Oui, tous les jours	avant_cannabis cannab.
**G8. Combien de fois vous est-il arrivé de consommer de l'alcool au cours de**	avant_alcool

**l'année écoulée ?**<br><br>☐ Jamais<br>☐ Moins d'une fois par mois<br>☐ Une fois par mois<br>☐ 2 à 4 fois par mois<br>☐ 2 à 3 fois par semaine<br>☐ 4 fois ou plus par semaine	freqmois.
**G9. #Si alcool au moins une fois par mois, # Combien de verres standard buvez-vous au cours d'une journée ordinaire où vous buvez de l'alcool ?**<br><br>*Un verre d'alcool correspond à un ballon de vin (10 cl), un demi de bière (25 cl) ou un verre d'alcool fort (4 cl).*<br><br>☐ Un ou deux<br>☐ Trois ou quatre<br>☐ Cinq ou six<br>☐ Sept à neuf<br>☐ Dix ou plus	avant_verre nbverre.
**G10. #Si alcool au moins une fois par mois, # Au cours d'une même occasion, combien de fois vous est-il arrivé de boire 6 verres standard ou plus (au cours de l'année écoulée) ?**<br><br>☐ Jamais<br>☐ Moins de 1 fois par mois<br>☐ 1 fois par mois<br>☐ Une fois par semaine<br>☐ Chaque jour ou presque	avant_6verres freqverrres.

PARTIE H. Vos activités

(page 12)

**H1. Au cours des 7 derniers jours, avez-vous porté un masque quand vous êtes allé.e dehors (faire les courses par exemple) ?**<br>☐ Oui, tout le temps<br>☐ Oui, parfois<br>☐ Non, car je n'ai pas de masque mais sinon j'en porterai un<br>☐ Non, car je ne crois pas que ce soit important<br>☐ Non concerné.e	`masque_dehors masque.`
**H2. Actuellement, sur une échelle de 0 à 10 (0=pas du tout, 10=totalement), à quel point vous sentez-vous seul.e ?**<br>0 1 2 3 4 5 6 7 8 9 10	`seul_eva likert.`

Q. A quelle fréquence avez-vous des interactions sociales avec votre famille ou des amis ?

		Jamais	Moins d'une fois par semaine	Une fois par semaine	Plusieurs fois par semaine	Tous les jours	
H3.	**Directement en face à face**	❑	❑	❑	❑	❑	`interFreq_ftf freqsem5.`
H4.	**Au téléphone**	❑	❑	❑	❑	❑	`interFreq_tel freqsem5.`
H5.	**Par SMS**	❑	❑	❑	❑	❑	`interFreq_sms freqsem5.`
H6.	**Sur les réseaux sociaux**	❑	❑	❑	❑	❑	`interFreq_rs freqsem5.`

PARTIE I. Vos perceptions sur la santé

(page 13)

Veuillez indiquer dans quelle mesure vous êtes d'accord ou non avec chacune des affirmations suivantes.

		Pas du tout d'accord	Pas d'accord	D'accord	Tout à fait d'accord	
I6.	Je compare les informations sur la santé qui viennent de différentes sources	❑	❑	❑	❑	infosante_compare accord.
I7.	Quand je découvre une nouvelle information sur la santé, je vérifie si elle est vraie ou non	❑	❑	❑	❑	infosante_verifie accord.
I8.	Je compare toujours les informations sur la santé à partir de différentes sources et je décide ce qui est le mieux pour moi	❑	❑	❑	❑	infosante_sources accord.
I9.	Je sais déterminer si une information sur la santé est adaptée à ma situation ou pas	❑	❑	❑	❑	infosante_determine accord.
I10.	J'interroge les professionnels de santé sur la qualité des informations que je trouve	❑	❑	❑	❑	infosante_pro accord.

PARTIE J. Vous gardez le moral ?

(page 14/15)

J1. Comment jugez-vous actuellement votre moral sur une échelle de 0 à 10 ?
Donnez une note entre 0 (très mauvais) et 10 (très bon).

0 1 2 3 4 5 6 7 8 9 10

moral_act likert.

J2. À quel point êtes-vous inquiet(ète) ou stressé(e) en ce moment sur une échelle de 0 à 10 ?
Donnez une note entre 0 (no stress) et 10 (très stressé(e)).

0 1 2 3 4 5 6 7 8 9 10

stress_act likert.

J3. Au cours des 7 derniers jours, avez-vous mis en place des routines pour avoir un rythme de vie régulier ? (ex. dîner toujours à la même heure, lire tous les soirs avant de dormir, appeler tous les jours vos proches)

routine rout.

- ❒ Oui et j'arrive à m'y tenir
- ❒ Oui, mais je ne les respecte pas tout le temps
- ❒ Non, mon rythme de vie en ce moment est plutôt déréglé
- ❒ Non, je n'en ai pas besoin

J4. Au cours des 7 derniers jours, comment avez-vous dormi ?

dormi_qlt qual.

- ❒ Bien
- ❒ Plutôt bien
- ❒ Ni bien ni mal
- ❒ Plutôt mal
- ❒ Mal

J5. Au cours des 7 derniers jours, avez-vous eu des difficultés d'endormissement et/ou de maintien de votre sommeil (réveils nocturnes) ?

- ❒ Jamais ou moins d'1 fois par mois
- ❒ Moins d'1 fois par semaine
- ❒ 1 à 2 jours par semaine
- ❒ 3 à 5 jours par semaine
- ❒ Tous les jours ou presque

dormi_diff freqdorm.

J6. Au cours des 7 derniers jours, vous êtes-vous senti extrêmement somnolent durant la journée ?

- ❒ Jamais ou moins d'1 fois par mois
- ❒ Moins d'1 fois par semaine

dormi_somn

❒ 1 à 2 jours par semaine<br>❒ 3 à 5 jours par semaine<br>❒ Tous les jours ou presque	freqdorm.
**J7. Au cours des 7 derniers jours, quelle a été votre heure de lever en moyenne ?**<br>|__| __| : |__| __|	dormi_lever hhmm.
**J8. Au cours des 7 derniers jours, quelle a été votre heure de coucher en moyenne ?**<br>|__| __| : |__| __|	dormi_coucher hhmm.
**J9. Au cours des 7 derniers jours, comment était votre rythme de lever et de coucher ?**<br>❒ Très irrégulier<br>❒ Plutôt irrégulier<br>❒ Ni régulier ni irrégulier<br>❒ Plutôt régulier<br>❒ Très régulier	dormi_rythme rythme.

Q. <u>Au cours des 7 derniers jours</u>, comment ont évolué vos consommations ?

		Je n'en consomme pas habituel-lement	Iden-tique	Diminution ou arrêt sans manque	Dimi-nution ou arrêt avec manque	Augmen-tation modérée	Augmen-tation difficile à contrôler	
**J10.**	**Café, thé et/ou boissons énergisantes**	❑	❑	❑	❑	❑	❑	evolconso_cafe evolconso.
**J11.**	**Aliments gras, sucrés et/ou salés**	❑	❑	❑	❑	❑	❑	evolconso_sucre evolconso.
**J12.**	**Tabac**	❑	❑	❑	❑	❑	❑	evolconso_tabac evolconso.
**J13.**	**Cigarette électronique**	❑	❑	❑	❑	❑	❑	evolconso_ecig evolconso.
**J14.**	**Alcool**	❑	❑	❑	❑	❑	❑	evolconso_alcool evolconso.
**J15.**	**Cannabis**	❑	❑	❑	❑	❑	❑	evolconso_cannabis evolconso.
**J16.**	**Autres drogues**	❑	❑	❑	❑	❑	❑	evolconso_drogu

	**(ecstasy…)**							es evolconso.
J17.	**Médicaments pour dormir**	❑	❑	❑	❑	❑	❑	evolconso_medic evolconso.
J18.	**Anxiolytiques (alprazolam…)**	❑	❑	❑	❑	❑	❑	evolconso_anxio evolconso.
J19.	**Ecrans** (TV, smartphone, tablette, ordinateur…)	❑	❑	❑	❑	❑	❑	evolconso_ecran evolconso.

J20.#Si alcool au moins 6 verres <> Jamais, # Si cela vous arrive de boire 6 verres d'alcool en une seule occasion et sur un temps court : cela vous arrive-t-il plus fréquemment depuis le confinement ?

alcool_conso alcool.

- ☐ Non, la fréquence de ces épisodes n’a pas changé
- ☐ Oui, la fréquence a diminué/ j’ai arrêté
- ☐ Oui, la fréquence a un peu augmenté
- ☐ Oui, elle a un beaucoup augmenté
- ☐ *Je ne suis pas concerné(e)*

J21. Au cours des 7 derniers jours, vous est-il arrivé de penser à vous suicider (d’avoir des idées suicidaires) ?

suicide onqqf.

- ❒ Non, jamais
- ❒ Oui, quelquefois
- ❒ Oui, à de multiples reprises

# Si oui, #

J22. Avez-vous pensé à la manière dont vous vous y prendriez ?

suic_maniere suicman.

- ☐ Non
- ☐ Oui, mais pas dans le détail
- ☐ Oui, j’ai réfléchi à un scénario précis

J23. Vous est-il arrivé de penser à vous suicider (d’avoir des idées suicidaires) au cours des 12 derniers mois qui ont précédé le début de l’épidémie ?

suic_avant onqqf.

- ☐ Non, jamais
- ☐ Oui, quelquefois
- ☐ Oui, à de multiples reprises

Quelle note donneriez-vous à votre qualité de vie au cours des 7 derniers jours ?

(0 pour la pire qualité de vie possible et 10 pour la meilleure qualité de vie possible)<br><br>0 1 2 3 4 5 6 7 8 9 10	qdv_act likert.

Questions complémentaires à destination des étudiants en médecine et autres études en santé

```
Si etude_filiere = 21 OU etude_filiere = 35 OU
etude_domaine = 4 OU etude_domaine = 5

Si prof_soigne = 1 → questions ES9 jusqu'à ES24
```

**ES1. A quel métier vous destinez-vous ?**<br>☐ Médecin<br>☐ Pharmacie.ne<br>☐ Chirurgien.ne dentiste<br>☐ Sage-femme<br>☐ Kinésithérapeute<br>☐ Ostéopathe<br>☐ Chiropracteur.rice<br>☐ Orthophoniste<br>☐ Psychomotricien.ne<br>☐ Ergothérapeute<br>☐ Psychologue<br>☐ Infirmier.ère<br>☐ Manipulateur.rice en électroradiologie<br>☐ Technicien.ne de laboratoire<br>☐ Pédicure-podologue<br>☐ Diététicien.ne<br>☐ Orthoptiste<br>☐ Opticien.ne<br>☐ Audioprothésiste<br>☐ Prothésiste dentaire<br>☐ Ingénieur.e biomédical<br>☐ Ingénieur.e biomécanique<br>☐ Ergonome<br>☐ Etiopathe<br>☐ Préparateur.rice en pharmacie<br>☐ Educateur.rice spécialisé<br>☐ Socio-esthéticien.ne<br>☐ Art-thérapeute<br>☐ Aide-soignant.e	`metier metier.`

☐ Auxiliaire de puériculture<br>☐ Secrétaire médicale<br>☐ Ambulancier.ère<br>☐ Autre	
**ES2. Avant l'arrivée des cas de COVID-19 et le confinement, étiez-vous en stage hospitalier ou ambulatoire ?**<br>☐ Oui ☐ Non<br><br>**ES3. Dans le cadre de la prise en charge des patients COVID-19, avez-vous été réaffecté, réquisitionné ou avez-vous répondu à une demande de mobilisation des instances sanitaires ou établissements de formation/universités ?**<br>☐ Oui ☐ Non<br><br>*# Si Non, poser uniquement ES4, ES9 puis ES22 à ES24#*<br>*# Si Oui, poser les questions ES5 à ES24#*<br><br>**ES4. Auriez-vous préféré être réaffecté/réquisitionné/mobilisé sur une mission en lien avec le COVID-19 à la place de la poursuite de votre stage ou de la poursuite de vos cours ?**<br>☐ Oui ☐ Non<br><br>**ES5. Précisez les conditions de votre réaffectation.**<br>☐ Inscription sur la liste de réserve sanitaire<br>☐ Autre type de volontariat<br>☐ Réquisition<br>☐ Réaffectation par l'université/ école sur un autre lieu de stage validant<br>☐ Autre<br><br>**ES6. Quel(s) type (s) de mission(s) avez-vous effectué dans le cadre de cette réaffectation/réquisition/mobilisation ?** *Plusieurs réponses possibles*<br>☐ Soutien au personnel d'une structure médico-sociale autre qu'un hôpital/centre Covid (Ehpad, …)<br>☐ Soutien au personnel soignant à l'hôpital ou en clinique<br>☐ Service téléphonique du SAMU<br>☐ Service téléphonique de soutien (à la population générale, à des professionnels de santé, à des étudiants, etc.)<br>☐ Activité(s) de recherche en lien le COVID-19<br><br>**ES7. Ces missions ont-elles été effectuées en lieu et place de votre stage initial d'affectation ou les faîtes-vous en plus ?**<br>☐ Elles sont effectuées à la place du stage initial	`stage_hosp ouinon.`<br><br>`requis ouinon.`<br><br>`requis_pref ouinon.`<br><br>`requis_insc coch.`<br>`requis_volont coch.`<br>`requis_requis coch.`<br>`requis_reaff coch.`<br>`requis_autre coch.`<br><br>`mission_medisoc coch.`<br>`mission_hop coch.`<br>`mission_samu coch.`<br>`mission_pop coch.`<br>`mission_recherche coch.`<br><br>`mission_stage`

☐ Je les effectue en plus du stage et sur mon temps libre
☐ Je les effectue en plus du stage mais celui-ci a été aménagé en conséquence, sans empiéter sur mon temps libre

missstage.

ES8. Ces missions ont-elles modifié votre quotité horaire moyenne de travail hebdomadaire ?

☐ Oui, elle a augmenté
☐ Oui, elle a diminué
☐ Non, elle est identique

mission_horaire evolhor.

# A partir de ES9 jusqu'à ES24, questions à proposer également au personnel soignant #

ES9. Dans le cadre de votre exercice ou formation professionnelle (stages, missions…), dans quelle mesure avez-vous été en contact avec des patients ou des professionnels qui avaient ou étaient suspects d'avoir le COVID-19 ?

☐ Oui, j'ai eu des contacts étroits (<1m) **sans** équipements de protection
☐ Oui, j'ai eu des contacts étroits (<1m) **avec** équipements de protection
☐ Oui, j'ai eu des contacts à distance (>1m)
☐ Non, je n'ai eu aucun contact

contact contact.

Dans le cadre de votre exercice ou formation professionnelle (stages, missions…), quel était votre degré d'inquiétude à propos de :

	**Pas du tout inquiet.ète**	**Un peu inquiet.ète**	**Moyen-nement inquiet.ète**	**Très inquiet.ète**	**Enormé-ment inquiet.ète**	***Je ne suis pas concer-né.e***	
**ES10. Votre risque de contracter le COVID-19**	❑	❑	❑	❑	❑	❑	psinqu_covid19 inq6.
**ES11. Le risque de contaminer d'autres patients**	❑	❑	❑	❑	❑	❑	psinqu_contpat inq6.
**ES12. Le risque de contaminer vos proches**	❑	❑	❑	❑	❑	❑	psinqu_contproche inq6.

**ES13. Votre capacité à respecter les règles d'hygiène au cours de vos missions**	❑	❑	❑	❑	❑	❑	psinqu_hyg inq6.
**ES14. Votre capacité à avoir les compétences suffisantes**	❑	❑	❑	❑	❑	❑	psinqu_compet inq6.
**ES15. Votre capacité à gérer la charge de travail**	❑	❑	❑	❑	❑	❑	psinqu_charge inq6.
**ES16. Votre capacité à gérer la fatigue**	❑	❑	❑	❑	❑	❑	psinqu_fatigue inq6.
**ES17. Votre capacité à gérer les décès**	❑	❑	❑	❑	❑	❑	psinqu_deces inq6.

ES18. Dans le cadre de votre exercice ou formation professionnelle (stages, missions…), est-ce que l'un (ou plusieurs) de vos collègues a contracté le COVID-19? covid_coll ouinon.

☐ Oui ☐ Non

Dans le cadre de votre exercice ou de formation professionnelle (stages, missions…), estimez-vous avoir disposé :

ES19. Des équipements de protection nécessaires (masques, blouses, gants, solution hydro-alcoolique…) dispo_equip ouinepi.

☐ Oui, dès le début de l'épidémie
☐ Oui, mais pas dans les premiers jours de l'épidémie
☐ Non, à aucun moment les équipements n'ont été suffisants
☐ Je ne suis pas concerné.e

ES20. D'une formation suffisante dispo_form onnc.

☐ Oui ☐ Non ☐ Je ne suis pas concerné.e

**ES21.** **D'un soutien psychologique mobilisable pour vous, si vous le jugez nécessaire**<br>☐ Oui ☐ Non ☐ Je ne suis pas concerné.e	dispo_soutien onnc.
ES22. **Au cours de votre vie avant le confinement, aviez-vous déjà vécu, ou été le témoin d'un évènement très grave, au cours duquel des personnes ou vous-même ont été menacées de mort ou ont été grièvement blessées ou ont été atteintes dans leur intégrité physique ?** ***Exemple de contextes traumatiques : accident grave, agression, viol, attentat, incendie, découverte de cadavre, mort subite dans l'entourage, guerre, catastrophe naturelle …***<br>❒ Oui<br>❒ Non<br>❑ Ne souhaite pas répondre	evgrave onsht.
# Si oui, poser ES23 #<br>***ES23.*** **Depuis le début du confinement, avez-vous souvent pensé de façon pénible à cet évènement de votre passé, ou en avez-vous souvent rêvé, ou avez-vous eu fréquemment l'impression de le revivre ?**<br>❒ Oui<br>❒ Non<br>❑ Ne souhaite pas répondre	evgrave_pense onsht.

ES24. **Nous allons maintenant vous poser des questions sur des difficultés que les gens éprouvent parfois à la suite d'un évènement stressant. « L'événement » en ce qui vous concerne peut être aussi bien l'épidémie en cours et le confinement qu'un événement précis qui vous serait arrivé lors de votre activité professionnelle (être en contact avec un patient covid ou assister à une réanimation par exemple). Veuillez lire attentivement chaque item et indiquer à quel point vous avez été affecté.e ou bouleversé.e au cours de ces 7 derniers jours.**

	Pas du tout	Un peu	Moyen-nement	Beau-coup	Enor-mément	
Tout rappel de l'événement ravivait mes sentiments face à l'événement.	❑	❑	❑	❑	❑	diff_rappel diff5.
Je me réveillais la nuit.	❑	❑	❑	❑	❑	diff_reveil diff5.
Différentes choses m'y faisaient penser.	❑	❑	❑	❑	❑	diff_pense diff5.
Je me sentais irritable et en colère.	❑	❑	❑	❑	❑	diff_colere diff5.
Quand j'y repensais ou	❑	❑	❑	❑	❑	

qu'on me le rappelait, j'évitais de me laisser bouleverser.							diff_bouleverse diff5.
Sans le vouloir, j'y repensais.	❑	❑	❑	❑	❑		diff_repense diff5.
J'ai eu l'impression que l'événement n'était jamais arrivé ou n'était pas réel.	❑	❑	❑	❑	❑		diff_irreel diff5.
Je me suis tenu loin de ce qui m'y faisait penser.	❑	❑	❑	❑	❑		diff_loin diff5.
Des images de l'événement surgissaient dans ma tête.	❑	❑	❑	❑	❑		diff_image diff5.
J'étais nerveux (nerveuse) et je sursautais facilement.	❑	❑	❑	❑	❑		diff_nerveux diff5.
J'essayais de ne pas y penser.	❑	❑	❑	❑	❑		diff_nepaspenser diff5.
J'étais conscient.e d'avoir encore beaucoup d'émotions à propos de l'événement.	❑	❑	❑	❑	❑		diff_emotion diff5.
Mes sentiments à propos de l'événement étaient comme figés.	❑	❑	❑	❑	❑		diff_fige diff5.
Je me sentais et je réagissais comme si j'étais encore dans l'événement.	❑	❑	❑	❑	❑		diff_encore diff5.
J'avais du mal à m'endormir.	❑	❑	❑	❑	❑		diff_dormir diff5.
J'ai ressenti des vagues de sentiments intenses à propos de l'événement.	❑	❑	❑	❑	❑		diff_vague diff5.
J'ai essayé de l'effacer de ma mémoire.	❑	❑	❑	❑	❑		

J'avais du mal à me concentrer.	❑	❑	❑	❑	❑	diff_efface diff5. diff_concentre diff5.
Ce qui me rappelait l'événement me causait des réactions physiques telles que des sueurs, des difficultés à respirer, des nausées ou des palpitations.	❑	❑	❑	❑	❑	diff_physique diff5.
J'ai rêvé à l'événement.	❑	❑	❑	❑	❑	
J'étais aux aguets, sur mes gardes.	❑	❑	❑	❑	❑	diff_reve diff5.
J'ai essayé de ne pas en parler.	❑	❑	❑	❑	❑	diff_gerde diff5. diff_parle diff5.

ES25. Y a t'il pour votre pratique ou votre formation un avant et un après covid ? Qu'est ce que ça a changé ?

exprime_prof $3000.

Questionnaire DECONFINEMENT 1

Date de remplissage du questionnaire : |__|__|/ |__|__|/ 2020
Jour Mois

(Date système remplie de manière automatique)

Partie A. Vos perceptions sur l'épidémie en cours

**M1. Pensez-vous avoir contracté le COVID-19 ?**<br>❐ Oui, j'ai été testé(e) positif<br>❐ Oui, cela a été évoqué par un médecin mais je n'ai pas été testé o<br>❐ u j'ai été testé négatif<br>❐ C'est possible, je présente des symptômes (fièvre, toux, courbatures, fatigue intense, diarrhées, douleur thoracique, perte de l'odorat, gêne respiratoire)<br>❐ C'est peu probable, je ne me sens pas malade<br>❐ Je suis certain(e) de ne pas être atteint(e)<br>❐ Je ne sais pas<br><br>**# Si Oui, j'ai été testé(e) positif, ajouter M2 et M3 #**<br><br>**M2. Pensez-vous avoir contracté le COVID-19 au cours de votre exercice professionnel ?**<br>❐ Oui, j'en suis certain.e<br>❐ Oui, c'est possible<br>❐ Non, je ne pense pas<br>❐ Je ne sais pas<br><br>**M3. Avez-vous été hospitalisé.e pour avoir contracté le COVID-19 ?**<br>❐ Oui<br>❐ Non	covid_atteint atteint.<br><br>covid_atteint_pro attpro.<br><br>covid_atteint_hosp ouinon.
**M4. Y a-t-il, dans votre entourage ou votre famille, des personnes qui ont eu le COVID-19 ou des signes de maladie laissant à penser que c'était le COVID-19 ?**<br>❐ Oui<br>❐ Non<br><br>**# Si Oui, → M5 et M6 #**<br><br>**M5. Un de vos proches a-t-il été hospitalisé pour avoir contracté le COVID-19 ?**<br>❐ Oui	covid_fam ouinon.<br><br>covid_fam_hosp

❒ Non — ouinon.

M6. Un de vos proches est-il décédé des suites du COVID-19 ? — covid_fam_deces ouinon.

❒ Oui
❒ Non

M7. Dans le contexte de cette épidémie, quel est votre degré d'inquiétude à propos de :

	1 Pas du tout inquiet(ète)	2	3	4	5 Très inquiet(ète)	Non concerné(e)	
Votre santé (infection par le COVID-19)	❑	❑	❑	❑	❑	❑	inq_sante inquiet.
La santé de vos parents	❑	❑	❑	❑	❑	❑	inq_parent inquiet.
La santé d'un de vos proches, de vos amis	❑	❑	❑	❑	❑	❑	inq_amis inquiet.
Votre situation financière	❑	❑	❑	❑	❑	❑	inq_finance inquiet.

M8. Que pensez-vous de l'évolution de l'épidémie ? (Plusieurs réponses possibles)

❒ L'épidémie va encore durer quelques semaines puis tout va rentrer dans l'ordre et nous reprendrons nos vies comme avant — epidemie_evol1 coch.
❒ L'épidémie va évoluer par cycles pendant plusieurs mois et il pourra y avoir des phases de reconfinement — epidemie_evol6 coch.
❒ Le coronavirus va rester parmi nous pendant plusieurs années et il faudra continuer à faire attention (gestes barrières, distanciation sociale) — epidemie_evol7 coch.
❒ Sans opinion — epidemie_evol5 coch.

Partie B. Votre quotidien face à l'épidémie

**M9. Quelle est votre situation professionnelle actuelle?**<br>☐ Vous travaillez sur votre lieu de travail à temps complet<br>☐ Vous travaillez sur votre lieu de travail à temps partiel<br>☐ Vous télétravaillez à temps complet<br>☐ Vous avez une activité mixte entre télétravail et présence sur votre lieu de travail<br>☐ Vous avez trouvé un emploi<br>☐ Vous êtes au chômage technique ou partiel<br>☐ Vous êtes en congés forcés<br>☐ Vous êtes en arrêt de travail pour garde d'enfants<br>☐ Vous êtes en arrêt de travail pour maladie<br>☐ Vous avez perdu votre emploi du fait de la crise sanitaire<br>☐ Autre	evol_prof evolpro.
**M10.** # Si profession cochée (autre que « pas de profession ») # **Indiquez votre degré d'inquiétude par rapport à votre emploi (fin de CDD, licenciement,…) :**<br>*Donnez une note entre 0 (pas du tout inquiet.ète) et 10 (très inquiet.ète).*<br>*0 1 2 3 4 5 6 7 8 9 10*	inq_travail likert.
**M11. Indiquez votre degré d'inquiétude par rapport à vos études (report des examens, validation de l'année, report de la rentrée, report des soutenances…) :**<br>*Donnez une note entre 0 (pas du tout inquiet(ète)) et 10 (très inquiet(ète)).*<br>*0 1 2 3 4 5 6 7 8 9 10*	inq_etude likert.
**M12. Si vous devez étudier ou travailler de chez vous, comment évaluez-vous votre capacité à effectuer les tâches qui sont attendues de vous dans votre environnement actuel ?**<br>❒ Je suis loin de fournir le travail attendu et c'est un problème pour moi<br>❒ Je ne suis pas aussi performant.e/concentré.e que d'habitude mais j'arrive à travailler<br>❒ Je suis satisfait.e de ce que j'arrive à faire<br>❒ Je travaille plutôt mieux que d'habitude<br>❒ Je ne suis pas concerné.e par cette question	eval_travail evaltr.
**M13. <u>Au cours des 7 derniers jours</u>, avez-vous pratiqué une activité physique <u>en intérieur</u> (ex. gymnastique, vélo d'appartement, …) ?**<br>❒ Non<br>❒ Oui, mais pas tous les jours<br>❒ Oui, tous les jours	sport_int tsjours.
**M14. <u>Au cours des 7 derniers jours</u>, avez-vous pratiqué une activité physique <u>en extérieur</u> (ex. marche, footing, …) ?**<br>❒ Non<br>❒ Oui, mais pas tous les jours	sport_ext tsjours.

❒ Oui, tous les jours

M15. **Actuellement, sur une échelle de 0 à 10 (0=pas du tout, 10=totalement), à quel point vous sentez-vous seul.e ?**

0 1 2 3 4 5 6 7 8 9 10

seul_eva likert.

Actuellement, à quelle fréquence avez-vous des interactions sociales avec votre famille ou des amis ?

		Jamais	Moins d'une fois par semaine	Une fois par semaine	Plusieurs fois par semaine	Tous les jours	
**M16.**	**Directement en présentiel**	❑	❑	❑	❑	❑	interFreq_ftf freqsem5.
**M17.**	**Au téléphone**	❑	❑	❑	❑	❑	interFreq_tel freqsem5.
**M18.**	**Par SMS**	❑	❑	❑	❑	❑	interFreq_sms freqsem5.
**M19.**	**Sur les réseaux sociaux**	❑	❑	❑	❑	❑	interFreq_rs freqsem5.

Au cours des 7 derniers jours, comment ont évolué vos consommations ?

		Je n'en consomme pas habituellement	Identique	Diminution ou arrêt sans manque	Diminution ou arrêt avec manque	Augmentation modérée	Augmentation difficile à contrôler	
**M20.**	**Café, thé et/ou boissons énergisantes**	❑	❑	❑	❑	❑	❑	evolconso_cafe evolconso.
**M21.**	**Aliments gras, sucrés et/ou salés**	❑	❑	❑	❑	❑	❑	evolconso_sucre evolconso.
**M22.**	**Tabac**	❑	❑	❑	❑	❑	❑	evolconso_tabac evolconso.
**M23.**	**Cigarette électronique**	❑	❑	❑	❑	❑	❑	evolconso_ecig evolconso.
**M24.**	**Alcool**	❑	❑	❑	❑	❑	❑	evolconso_alcool evolconso.
**M25.**	**Cannabis**	❑	❑	❑	❑	❑	❑	evolconso_cannabis evolconso.

**M26.**	**Autres drogues (ecstasy…)**	❑	❑	❑	❑	❑	❑	evolconso_drogues evolconso.
**M27.**	**Médicaments pour dormir**	❑	❑	❑	❑	❑	❑	evolconso_medic evolconso.
**M28.**	**Anxiolytiques (alprazolam…)**	❑	❑	❑	❑	❑	❑	evolconso_anxio evolconso.
**M29.**	**Ecrans (TV, smartphone, tablette, ordinateur…)**	❑	❑	❑	❑	❑	❑	evolconso_ecran evolconso.

M30. Respectez-vous les consignes sanitaires (distanciation physique, port de masque, …) telles que préconisées par les pouvoirs publics ?

- ❒ Oui, strictement
- ❒ Oui, mais j'ai fait quelques entorses
- ❒ Non, j'essaye d'en tenir compte mais je ne les respecte pas vraiment
- ❒ Non, pas du tout

respect_consSan consig.

Partie C. Votre moral

M31. Comment jugez-vous actuellement votre moral sur une échelle de 0 à 10 ?

Donnez une note entre 0 (très mauvais) et 10 (très bon).

0 1 2 3 4 5 6 7 8 9 10

moral_act likert.

M32. À quel point êtes-vous inquiet(ète) ou stressé(e) en ce moment sur une échelle de 0 à 10 ?

Donnez une note entre 0 (no stress) et 10 (très stressé(e)).

0 1 2 3 4 5 6 7 8 9 10

stress_act likert.

M33. <u>Au cours des 7 derniers jours</u>, comment avez-vous dormi ?

- ❒ Bien
- ❒ Plutôt bien
- ❒ Ni bien ni mal
- ❒ Plutôt mal
- ❒ Mal

dormi_qlt qual.

M34. **<u>Au cours des 7 derniers jours,</u> avez-vous eu des difficultés d'endormissement et/ou de maintien de votre sommeil (réveils nocturnes) ?**

- ❒ Jamais
- ❒ Moins d'1 fois par semaine
- ❒ 1 à 2 jours par semaine
- ❒ 3 à 5 jours par semaine
- ❒ Tous les jours ou presque

`dormi_diff freqdorm.`

M35. **<u>Au cours des 7 derniers jours</u>, vous êtes-vous senti extrêmement somnolent durant la journée ?**

- ❒ Jamais
- ❒ Moins d'1 fois par semaine
- ❒ 1 à 2 jours par semaine
- ❒ 3 à 5 jours par semaine
- ❒ Tous les jours ou presque

`dormi_somn freqdorm.`

M36. **<u>Au cours des 7 derniers jours</u>, quelle a été votre heure de lever en moyenne ?** *(numérique)*

|__| __| : |__| __|

`dormi_lever hhmm.`

`dormi_coucher hhmm.`

M37. **<u>Au cours des 7 derniers jours</u>, quelle a été votre heure de coucher en moyenne ?** *(numérique)*

|__| __| : |__| __|

M38. **<u>Au cours des 7 derniers jours</u>, comment était votre rythme de lever et de coucher ?**

- ❒ Très irrégulier
- ❒ Plutôt irrégulier
- ❒ Ni régulier ni irrégulier
- ❒ Plutôt régulier
- ❒ Très régulier

`dormi_rythme rythme.`

M39. **Si cela vous arrive de boire 6 verres d'alcool en une seule occasion et sur un temps court : cela vous est-il arrivé plus fréquemment <u>au cours des 7 derniers jours</u>?**

- ☐ Non, la fréquence de ces épisodes n'a pas changé
- ☐ Oui, la fréquence a un peu augmenté
- ☐ Oui, elle a un beaucoup augmenté
- ☐ *Je ne suis pas concerné*

`alcool_conso alcool.`

Au cours des 2 dernière[illegible]

par les problèmes suivants ?

		Presque jamais	Plusieurs jours durant ces 2 dernières semaines	Plus de la moitié du temps	Presque tous les jours	
**M40.**	Peu d'intérêt ou de plaisir à faire les choses	❑	❑	❑	❑	pb_derang1 freq4.
**M41.**	Se sentir triste, déprimé.e ou désespéré.e	❑	❑	❑	❑	pb_derang2 freq4.
**M42.**	Difficultés à s'endormir ou à rester endormi.e, ou trop dormir	❑	❑	❑	❑	pb_derang3 freq4.
**M43.**	Se sentir fatigué.e ou avoir peu d'énergie	❑	❑	❑	❑	pb_derang4 freq4.
**M44.**	Peu d'appétit ou trop manger	❑	❑	❑	❑	pb_derang5 freq4.
**M45.**	Mauvaise perception de vous-même - ou vous pensez que vous êtes un.e perdant.e ou que vous n'avez pas satisfait vos propres attentes ou celles de votre famille	❑	❑	❑	❑	pb_derang6 freq4.
**M46.**	Difficultés à se concentrer sur des choses telles que lire le journal ou regarder la télévision	❑	❑	❑	❑	pb_derang7 freq4.
**M47.**	Vous bougez ou parlez si lentement que les autres personnes ont pu le remarquer. Ou au contraire - vous êtes si agité.e que vous bougez beaucoup plus que d'habitude	❑	❑	❑	❑	pb_derang8 freq4.
**M48.**	Vous avez pensé que vous seriez mieux mort.e ou	❑	❑	❑	❑	

	vous avez pensé à vous blesser d'une façon ou d'une autre					pb_derang9 freq4.

<u>Au cours des 2 dernières semaines</u>, à quelle fréquence avez-vous été gêné.e par les problèmes suivants ?

		**Presque jamais**	**Plusieurs jours au cours de ces 2 dernières semaines**	**Plus de la moitié du temps**	**Presque tous les jours**	
**M49.**	Un sentiment de nervosité, d'anxiété ou de tension	❑	❑	❑	❑	pb_gene1 freq4.
**M50.**	Une incapacité à arrêter de s'inquiéter ou à contrôler ses inquiétudes	❑	❑	❑	❑	pb_gene2 freq4.
**M51.**	Une inquiétude excessive à propos de différentes choses	❑	❑	❑	❑	pb_gene3 freq4.
**M52.**	Des difficultés à me détendre	❑	❑	❑	❑	pb_gene4 freq4.
**M53.**	Une agitation telle qu'il m'est difficile de tenir en place	❑	❑	❑	❑	pb_gene5 freq4.
**M54.**	Une tendance à être facilement contrarié.e ou irritable	❑	❑	❑	❑	pb_gene6 freq4.
**M55.**	Un sentiment de peur comme si quelque chose de terrible risquait de se produire	❑	❑	❑	❑	pb_gene7 freq4.

M56. **<u>Au cours des 2 dernières semaines</u>, vous est-il arrivé de penser à vous suicider (d'avoir des idées suicidaires) ?**

❒ Non, jamais<br>❒ Oui, quelquefois<br>❒ Oui, à de multiples reprises<br><br>***# Si oui, quelquefois ou Oui, à de multiples reprises, ajouter M57 ET M58 #***<br><br>**M57. Avez-vous pensé à la manière dont vous vous y prendriez ?**<br>❒ Non<br>❒ Oui, mais pas dans le détail<br>❒ Oui, j'ai réfléchi à un scénario précis<br><br>**M58. Vous est-il arrivé de penser à vous suicider (d'avoir des idées suicidaires) au cours des 12 derniers mois qui ont précédé le début de l'épidémie ?**<br>❒ Non, jamais<br>❒ Oui, quelquefois<br>❒ Oui, à de multiples reprises<br>**M59. Quelle note donneriez-vous à votre qualité de vie ?**<br>*(0 pour la pire qualité de vie possible et 10 pour la meilleure qualité de vie possible)*<br><br>0 1 2 3 4 5 6 7 8 9 10	suicide onqqf.<br><br>suic_maniere suicman.<br><br>suic_avant onqqf.<br><br>qdv_act likert.

Partie D. Votre avis sur les traitements et les vaccins

Nous allons maintenant vous poser des questions sur la façon dont vous vous renseignez sur la vaccination.

**M60. Seriez-vous prêt.e à vous faire vacciner contre le COVID-19 même si le vaccin n'a pas encore fait complètement la preuve de son efficacité ?**<br>☐ Oui, même s'il y a un risque lié au vaccin<br>☐ Oui, mais à condition que le risque lié au vaccin soit faible<br>☐ Oui, mais à condition qu'il n'y ait absolument aucun risque lié au vaccin, même faible<br>☐ Oui, dans le cadre d'un essai clinique (si un vaccin n'est pas encore validé)<br>☐ Non, jamais<br>☐ Je ne sais pas	vaccovid vaccovid.

Concernant la vaccination en général, à quel point êtes-vous d'accord avec les propositions suivantes ?

		Entiè-rement d'accord	Plutôt d'accord	Plutôt pas d'accord	Pas d'accord	Sans opinion	
M61.	La vaccination peut provoquer de graves effets secondaires	❑	❑	❑	❑	❑	vac_effsec accord5.
M62.	La vaccination n'a pas beaucoup d'intérêt, car il y a très peu de risque d'être infecté.e	❑	❑	❑	❑	❑	vac_nointeret accord5.
M63.	Il n'est pas nécessaire d'être vacciné.e car beaucoup de gens le sont autour de nous	❑	❑	❑	❑	❑	vac_nonec accord5.
M64.	Si un vaccin n'est pas obligatoire c'est qu'il n'est pas si important	❑	❑	❑	❑	❑	vac_nonoblig accord5.
M65.	Les industries pharmaceutiques incitent à se faire vacciner pour augmenter leurs bénéfices	❑	❑	❑	❑	❑	vac_benef accord5.
M66.	Se faire vacciner soi-même a un impact sur la santé des autres	❑	❑	❑	❑	❑	vaccin_impact accord5.

Concernant la recherche d'informations sur la vaccination, à quel point

êtes-vous d'accord avec les propositions suivantes ?

		Entièrement d'accord	Plutôt d'accord	Plutôt pas d'accord	Pas d'accord	Sans opinion	
M67.	Je pense qu'il est facile de se renseigner sur la vaccination sur Internet	❑	❑	❑	❑	❑	decvac_internet accord5.
M68.	Je trouve que les informations concernant la vaccination sur <u>les réseaux sociaux et les forums</u> sont compréhensibles	❑	❑	❑	❑	❑	decvac_rs accord5.
M69.	Je trouve que les informations concernant la vaccination sur les <u>sites gouvernementaux</u> sont compréhensibles	❑	❑	❑	❑	❑	decvac_gouv accord5.
M70.	Je suis capable de reconnaître les *fake news* sur le thème de la vaccination	❑	❑	❑	❑	❑	decvac_fakenews accord5.
M71.	J'ai confiance dans les informations fournies par les sites gouvernementaux	❑	❑	❑	❑	❑	decvac_gouv_conf accord5.
M72.	Je trouve que les informations sur la vaccination sur les réseaux sociaux sont valides	❑	❑	❑	❑	❑	decvac_rs_valide accord5.
M73.	Lorsque je prends connaissance	❑	❑	❑	❑	❑	

	d'une information sur la vaccination en ligne, je croise plusieurs sources afin de vérifier sa validité						decvac_source accord5.
M74.	Je pense que les informations trouvées en ligne peuvent influencer mon choix de me faire vacciner	❑	❑	❑	❑	❑	decvac_influe accord5.
M75.	Si je ne pense pas avoir assez de connaissances sur la vaccination et sur ses risques, je préfère ne pas me faire vacciner	❑	❑	❑	❑	❑	decvac_nonconnu accord5.
M76.	Si je ne pense pas avoir assez de connaissances sur la vaccination et sur ses risques, j'interroge les professionnels de santé que je connais	❑	❑	❑	❑	❑	decvac_prof accord5.

Questionnaire DECONFINEMENT 2

Date de remplissage du questionnaire : |__|__|/ |__|__|/ 2020
Jour Mois

(Date système remplie de manière automatique)

Partie A. Vos perceptions sur l'épidémie en cours

**M1. Pensez-vous avoir contracté la COVID-19 ?**<br>❒ Oui, j'ai été testé(e) positif<br>❒ Oui, cela a été évoqué par un médecin mais je n'ai pas été testé ou j'ai été testé négatif<br>❒ C'est possible, je présente des symptômes (fièvre, toux, courbatures, fatigue intense, diarrhées, douleur thoracique, perte de l'odorat, gêne respiratoire)<br>❒ C'est peu probable, je ne me sens pas malade<br>❒ Je suis certain(e) de ne pas être atteint(e)<br>❒ Je ne sais pas<br><br>**# Si Oui, j'ai été testé(e) positif, ajouter M2 et M3 #**<br><br>**M2. Pensez-vous avoir contracté la COVID-19 au cours de votre exercice professionnel ?**<br>❒ Oui, j'en suis certain.e<br>❒ Oui, c'est possible<br>❒ Non, je ne pense pas<br>❒ Je ne sais pas<br><br>**M3. Avez-vous été hospitalisé.e pour avoir contracté la COVID-19 ?**<br>❒ Oui<br>❒ Non	`covid_atteint atteint.`<br><br>`covid_atteint_pro attpro.`<br><br>`covid_atteint_hosp ouinon.`
**M4. Y a-t-il, dans votre entourage ou votre famille, des personnes qui ont eu la COVID-19 ou des signes de maladie laissant à penser que c'était la COVID-19 ?**<br>❒ Oui<br>❒ Non<br><br>**# Si Oui, → M5 et M6 #**<br><br>**M5. Un de vos proches a-t-il été hospitalisé pour avoir contracté la COVID-19 ?**<br>❒ Oui<br>❒ Non	`covid_fam ouinon.`<br><br>`covid_fam_hosp ouinon.`

**M6. Un de vos proches est-il décédé des suites de la COVID-19 ?**<br>❒ Oui<br>❒ Non	covid_fam_deces ouinon.
**M7. Dans le contexte de cette épidémie, quel est votre degré d'inquiétude à propos de :**	

	1<br>Pas du tout inquiet(ète)	2	3	4	5<br>Très inquiet(ète)	Non concerné(e)	
Votre santé (infection par la COVID-19)	❑	❑	❑	❑	❑	❑	inq_sante inquiet.
La santé de vos parents	❑	❑	❑	❑	❑	❑	inq_parent inquiet.
La santé d'un de vos proches, de vos amis	❑	❑	❑	❑	❑	❑	inq_amis inquiet.
Votre situation financière	❑	❑	❑	❑	❑	❑	inq_finance inquiet.

**M8. Que pensez-vous de l'évolution de l'épidémie ? (Plusieurs réponses possibles)**	
❒ L'épidémie va encore durer quelques semaines puis tout va rentrer dans l'ordre et nous reprendrons nos vies comme avant	epidemie_evol1 coch.
❒ L'épidémie va évoluer par cycles pendant plusieurs mois et il pourra y avoir des phases de reconfinement	epidemie_evol6 coch.
❒ Le coronavirus va rester parmi nous pendant plusieurs années et il faudra continuer à faire attention (gestes barrières, distanciation sociale)	epidemie_evol7 coch.
❒ Sans opinion	epidemie_evol5 coch.

Partie B. Votre quotidien face à l'épidémie

**M9. Quelle est votre situation professionnelle actuelle?**<br>☐ Vous travaillez sur votre lieu de travail à temps complet<br>☐ Vous travaillez sur votre lieu de travail à temps partiel<br>☐ Vous télétravaillez à temps complet<br>☐ Vous avez une activité mixte entre télétravail et présence sur votre lieu de travail<br>☐ Vous êtes au chômage technique ou partiel<br>☐ Vous êtes en arrêt de travail pour garde d'enfants<br>☐ Vous êtes en arrêt de travail pour maladie<br>☐ Vous avez perdu votre emploi du fait de la crise sanitaire<br>☐ Vous êtes à la retraite<br>☐ Autre	`evol_prof evolpro.`
**M10.** # Si profession cochée (autre que « pas de profession ») # **Indiquez votre degré d'inquiétude par rapport à votre emploi (fin de CDD, licenciement,…) :**<br>*Donnez une note entre 0 (pas du tout inquiet.ète) et 10 (très inquiet.ète).*<br>*0 1 2 3 4 5 6 7 8 9 10*	`inq_travail likert.`
**M11. Indiquez votre degré d'inquiétude par rapport à vos études (rentrée universitaire, validation de l'année, …) :**<br>*Donnez une note entre 0 (pas du tout inquiet(ète)) et 10 (très inquiet(ète)).*<br>*0 1 2 3 4 5 6 7 8 9 10*	`inq_etude likert.`
**M12. Au cours des 7 derniers jours, avez-vous pratiqué une activité physique ?**<br>❒ Non<br>❒ Oui, mais pas tous les jours<br>❒ Oui, tous les jours	`sport tsjours.`
**M13. Au cours des 7 derniers jours, sur une échelle de 0 à 10 (0=pas du tout, 10=totalement), à quel point vous sentez-vous seul.e ?**<br>0 1 2 3 4 5 6 7 8 9 10	`seul_eva likert.`

Partie C. Votre moral

**M14. À quel point êtes-vous inquiet(ète) ou stressé(e) en ce moment sur une échelle de 0 à 10 ?**<br>*Donnez une note entre 0 (no stress) et 10 (très stressé(e)).*<br>0 1 2 3 4 5 6 7 8 9 10	stress_act likert.
**M15. <u>Au cours des 7 derniers jours</u>, comment avez-vous dormi ?**<br>❒ Bien<br>❒ Plutôt bien<br>❒ Ni bien ni mal<br>❒ Plutôt mal<br>❒ Mal	dormi_qlt qual.
**M16. <u>Au cours des 7 derniers jours,</u> avez-vous eu des difficultés d'endormissement et/ou de maintien de votre sommeil (réveils nocturnes) ?**<br>❒ Jamais ou moins d'1 fois par mois<br>❒ Moins d'1 fois par semaine<br>❒ 1 à 2 jours par semaine<br>❒ 3 à 5 jours par semaine<br>❒ Tous les jours ou presque	dormi_diff freqdorm.
**M17. Si cela vous arrive de boire 6 verres d'alcool en une seule occasion et sur un temps court : cela vous est-il arrivé plus fréquemment <u>au cours des 7 derniers jours</u>?**<br>☐ Non, la fréquence de ces épisodes n'a pas changé<br>☐ Oui, la fréquence a un peu augmenté<br>☐ Oui, elle a un beaucoup augmenté<br>☐ *Je ne suis pas concerné*	alcool_conso alcool.

Nous vous proposons de répondre maintenant à plusieurs questions sur votre santé psychique. Si vous avez besoin de soutien psychologique, ou si certaines questions vous ont déstabilisé.e ou perturbé.e, n'hésitez pas à faire appel à l'un des nombreux dispositifs d'aide à distance dont vous trouverez les coordonnées dans l'onglet « Contact ».

<u>Au cours des 2 dernières semaines</u>, à quelle fréquence avez-vous été dérangé.e par les problèmes suivants ?

		Presque jamais	Plusieurs jours durant ces 2 dernières semaines	Plus de la moitié du temps	Presque tous les jours
**M18.**	Peu d'intérêt ou de plaisir à faire les choses	❑	❑	❑	❑
**M19.**	Se sentir triste, déprimé.e ou désespéré.e	❑	❑	❑	❑
**M20.**	Difficultés à s'endormir ou à rester endormi.e, ou trop dormir	❑	❑	❑	❑
**M21.**	Se sentir fatigué.e ou avoir peu d'énergie	❑	❑	❑	❑
**M22.**	Peu d'appétit ou trop manger	❑	❑	❑	❑
**M23.**	Mauvaise perception de vous-même - ou vous pensez que vous êtes un.e perdant.e ou que vous n'avez pas satisfait vos propres attentes ou celles de votre famille	❑	❑	❑	❑
**M24.**	Difficultés à se concentrer sur des choses telles que lire le journal ou regarder la télévision	❑	❑	❑	❑

```
pb_derang1 freq4.
pb_derang2 freq4.
pb_derang3 freq4.
pb_derang4 freq4.
pb_derang5 freq4.
pb_derang6 freq4.
pb_derang7 freq4.
```

M25.	Vous bougez ou parlez si lentement que les autres personnes ont pu le remarquer. Ou au contraire - vous êtes si agité.e que vous bougez beaucoup plus que d'habitude	❑	❑	❑	❑	pb_derang8 freq4.
M26.	Vous avez pensé que vous seriez mieux mort.e ou vous avez pensé à vous blesser d'une façon ou d'une autre	❑	❑	❑	❑	pb_derang9 freq4.

<u>Au cours des 2 dernières semaines</u>, à quelle fréquence avez-vous été gêné.e par les problèmes suivants ?

		**Presque jamais**	**Plusieurs jours au cours de ces 2 dernières semaines**	**Plus de la moitié du temps**	**Presque tous les jours**	
M27.	Un sentiment de nervosité, d'anxiété ou de tension	❑	❑	❑	❑	pb_gene1 freq4.
M28.	Une incapacité à arrêter de s'inquiéter ou à contrôler ses inquiétudes	❑	❑	❑	❑	pb_gene2 freq4.
M29.	Une inquiétude excessive à propos de différentes choses	❑	❑	❑	❑	pb_gene3 freq4.
M30.	Des difficultés à me détendre	❑	❑	❑	❑	pb_gene4 freq4.
M31.	Une agitation telle qu'il m'est difficile de tenir en place	❑	❑	❑	❑	pb_gene5 freq4.
M32.	Une tendance à être facilement contrarié.e	❑	❑	❑	❑	

	ou irritable					pb_gene6 freq4.
M33.	Un sentiment de peur comme si quelque chose de terrible risquait de se produire	❑	❑	❑	❑	pb_gene7 freq4.

M34. **<u>Au cours des 2 dernières semaines</u>, vous est-il arrivé de penser à vous suicider (d'avoir des idées suicidaires) ?**

- ❐ Non, jamais
- ❐ Oui, quelquefois
- ❐ Oui, à de multiples reprises

suicide onqqf.

# Si oui, quelquefois ou Oui, à de multiples reprises, → M35 ET M36 #

M35. **Avez-vous pensé à la manière dont vous vous y prendriez ?**

- ❐ Non
- ❐ Oui, mais pas dans le détail
- ❐ Oui, j'ai réfléchi à un scénario précis

suic_maniere suicman.

M36. **Vous est-il arrivé de penser à vous suicider (d'avoir des idées suicidaires) au cours des 12 derniers mois ?**

- ❐ Non, jamais
- ❐ Oui, quelquefois
- ❐ Oui, à de multiples reprises

suic_annee onqqf.

M37. **Actuellement, quelle note donneriez-vous à votre qualité de vie ?**
(0 pour la pire qualité de vie possible et 10 pour la meilleure qualité de vie possible)

0 1 2 3 4 5 6 7 8 9 10

qdv_act likert.

Partie D. Vos perceptions sur la santé

**Veuillez indiquer dans quelle mesure vous êtes d'accord ou non avec chacune des affirmations suivantes.**						
		Pas du tout d'accord	Pas d'accord	D'accord	Tout à fait d'accord	
**M. 21.**	Je compare les informations sur la santé qui viennent de différentes sources	❑	❑	❑	❑	infosante_compare accord.
**M. 22.**	Quand je découvre une nouvelle information sur la santé, je vérifie si elle est vraie ou non	❑	❑	❑	❑	infosante_verifie accord.
**M. 23.**	Je compare toujours les informations sur la santé à partir de différentes sources et je décide ce qui est le mieux pour moi	❑	❑	❑	❑	infosante_sources accord.
**M. 24.**	Je sais déterminer si une information sur la santé est adaptée à ma situation ou pas	❑	❑	❑	❑	infosante_determine accord.
**M. 25.**	J'interroge les professionnels de santé sur la qualité des informations que je trouve	❑	❑	❑	❑	infosante_pro accord.

QUESTIONNAIRE INCLUSION - (période reconfinement nov)

Critères d'éligibilité

**Q1. Date de naissance :** |__|__|/ |__|__|__|__| (≥ 18 ans)<br>**Pays de naissance** : ☐ France ☐ Autre<br>*# Si France, #* Précisez la commune<br>|__|__|__|__|__| (code postal)<br><br>**Q2. Etes vous étudiant ?**<br>☐ Oui ☐ Non<br><br>*Patient éligible si :*<br>• *Age ≥ 18 ans*<br>•	naiss_date MMYY10.<br>naiss_pays pays.<br>naiss_commune 5.<br><br>etudiant ouinon.

PARTIE A. Vos données sociodémographiques

(page 1)

**A1. Sexe :** ☐ Masculin ☐ Féminin ☐ Autre<br><br>**A2. Taille : |__|__|__|cm** **Poids : |__|__|__|kg**<br><br>**A3. Votre situation de famille**<br>☐ Célibataire<br>☐ En couple (depuis au moins 3 mois) sans être marié.e ni pacsé.e<br>☐ Marié.e, Pacsé.e<br>☐ Autre (divorcé.e, veuf.ve)<br><br>**A4. Avez-vous des enfants ?**<br>☐ Oui ☐ Non<br># Si oui, # combien : |__|__|	sexe sexe.<br><br>taille 3.<br>poids 3.<br><br>situation_fam sitfam.<br><br>etude_enfant ouinon.<br><br>enfant_nb 2.
**A5. Quel est votre domaine d'études ?**<br>☐ Sciences Humaines et Sociales, Lettres, Langues, Art<br>☐ Sciences, Technique, Ingénierie, Informatique<br>☐ Droit, Economie, Gestion, Finance, Sciences Politiques, Comptabilité	etude_domaine etdom.

☐ Médecine<br>☐ Autres études en Santé<br>☐ Autre<br><br>**A6. Quelle est votre filière d'études ?**<br>*#menu déroulant#*<br>81. Administration économique et sociale<br>82. Administration publique<br>83. Agronomie, agroalimentaire<br>84. Architecture, design<br>85. Arts (plastiques, du spectacle)<br>86. Commerce, vente<br>87. Droit<br>88. Economie et gestion<br>89. Electronique, énergie électrique, automatique<br>90. Génie civil<br>91. Géographie et aménagement<br>92. Histoire<br>93. Histoire de l'art et archéologie<br>94. Hôtellerie, restauration, tourisme<br>95. Information-communication<br>96. Informatique<br>97. Lettres, langues et civilisations étrangères et régionales<br>98. Mathématiques<br>99. Mathématiques et informatique appliquées aux sciences humaines et sociales<br>100. Mécanique<br>101. Médecine<br>102. Philosophie<br>103. Physique, chimie<br>104. Psychologie<br>105. Science politique<br>106. Sciences cognitives<br>107. Sciences de l'environnement<br>108. Sciences de la vie et de la Terre<br>109. Sciences de l'éducation<br>110. Sciences de l'homme, anthropologie, ethnologie<br>111. Sciences des matériaux<br>112. Sciences du langage<br>113. Sciences et techniques des activités physiques et sportives<br>114. Sciences et technologies<br>115. Sciences pour la santé autres que médecine<br>116. Sciences pour l'ingénieur<br>117. Sciences sanitaires et sociales<br>118. Sciences sociales<br>119. Télécommunications<br>120. Autre	`etude_filiere filet.`
Demander une seule fois par personne (étudiant uniquement) :	

**Dans quel type d'établissement de l'enseignement supérieur êtes-vous inscrit ?**<br>❒ Université<br>❒ Autre établissement<br>*#Si université#* **Région d'étude :**<br>*#menu déroulant#*<br>Auvergne-Rhône-Alpes<br>Bourgogne-Franche-Comté<br>Bretagne<br>Centre-Val de Loire<br>Collectivités d'outre-mer<br>Corse<br>Grand Est<br>Guadeloupe<br>Guyane<br>Hauts-de-France<br>Île-de-France<br>La Réunion<br>Mayotte<br>Normandie<br>Nouvelle-Aquitaine<br>Occitanie<br>Pays de la Loire<br>Provence-Alpes-Côte d'Azur<br>*#affichage des universités en fonction de la région choisie#*<br>*#menu déroulant#* + « Autre »<br>*#si autre#* **Préciser : ……………………………………..**<br>*#Si autre établissement#* **Préciser : ……………………………………..**	univ_type untype.<br>univ_region univreg.<br>universite univ.<br>universite_txt $255.<br>univ_autre $255.
**A7. En quelle année d'études êtes-vous ?**<br>☐ 1ère ☐ 2ème ☐ 3ème ☐ 4ème ☐ 5ème ☐ > 5ème<br>**A8. Participez-vous à l'étude i-Share ?**<br>❒ Oui<br>❒ Non<br>**A9. Concernant la situation économique de la famille qui vous a élevé(e) durant votre enfance et adolescence diriez-vous qu'elle était :**	etude_an annee.<br>Ishare ouinon.

☐ Très confortable<br>☐ Confortable<br>☐ Correcte<br>☐ Difficile<br>☐ Très difficile	enfance_siteco siteco.
**A10. Quelle est votre principale source de revenus pour le financement de votre année universitaire ? *(Une seule réponse possible)***<br>☐ Famille<br>☐ Bourse(s)<br>☐ Activités rémunérées<br>☐ Autre (économies, épargnes, prêt étudiant)	etude_revenu revenu.
**A11. A combien estimez-vous vos ressources financières mensuelles tout compris (participation des parents, bourses, aides étudiants étrangers, travail, épargne…) ?**<br>☐ Moins de 500 euros<br>☐ Entre 500 et 700 euros<br>☐ Entre 700 et 1000 euros<br>☐ Plus de 1000 euros	etude_reveuros reveuros.
**A12. Quel est votre diplôme le plus élevé ?**<br>☐ Aucun<br>☐ BEPC (Brevet des collèges), BEP, CAP, BP (Brevet professionnel)<br>☐ Baccalauréat (général, technologique ou professionnel)<br>☐ Bac+2 (DEUG, BTS, DUT ou équivalent)<br>☐ Bac+3 à Bac+4 (Licence, Master 1, DEI ou équivalent)<br>☐ Bac+5 ou plus (Master 2, école d'ingénieur, doctorat ou équivalent)	niveau_etude nivet.
**A17. Quand vous étiez étudiant, avez-vous participé à l'étude i-Share ?**<br>☐ Oui ☐ Non	nonet_ishare ouinon.
**A13. Quelle est votre profession ?**<br>☐ Agriculteur.trice (ex. éleveur.se, viticulteur.trice)<br>☐ Artisan, commerçant.e, chef.fe d'entreprise (ex. électricien.ne à son compte, gérant.e)<br>☐ Profession intermédiaire (ex. professeur.e des écoles, technicien.ne, infirmièr.e)<br>☐ Cadre, ingénieur.e, profession libérale (ex. médecin,	profession prof.

journaliste)<br>☐ Employé.e administratif d'entreprise ou de la fonction publique (ex. secrétaire, hôtesse de l'air/stewart, policier.e, aide-soignant.e)<br>☐ Employé.e de commerce (ex. caissièr.e, vendeur.se en magasin, pompiste)<br>☐ Personnel des services directs aux particuliers (ex. serveur.se, assistant.e maternelle)<br>☐ Ouvrier.e (ex. opérateur.e, chauffeur.e, peintre en bâtiment, magasinier.e)<br>☐ Vous n'avez pas de profession	
# Si profession cochée (autre que « pas de profession ») #<br>Votre profession est-elle en rapport avec le domaine de la santé ?<br>☐ Oui ☐ Non	prof_sante ouinon.
# Si oui, # Etes-vous soignant au contact de malades ?<br>☐ Oui ☐ Non	prof_soigne ouinon.
**A14. Quelle était votre situation professionnelle <u>avant l'épidémie ?</u>**<br>☐ En CDI ou fonctionnaire<br>☐ En CDD ou autre contrat court (saisonnier, vacataire, intérim, pigiste, CESU)<br>☐ Indépendant (y compris auto-entrepreneur, en freelance, libéral)<br>☐ Stagiaire, volontaire ou apprenti.e<br>☐ Vous avez un petit boulot non déclaré (ex. babysitting)<br>☐ Vous avez un autre type de contrat de travail<br>☐ Sans travail et à la recherche d'un emploi<br>☐ Au foyer<br>☐ Vous ne travaillez pas actuellement et vous ne cherchez pas d'emploi<br>☐ Retraité	situation_pro sitpro.
**A15. Quel est habituellement le montant des revenus mensuels de votre ménage (c'est-à-dire vous-même et votre conjoint.e si vous habitez avec quelqu'un) en incluant toutes les sources de revenus : salaires + allocations + pensions etc. ?**<br>☐ 500 euros ou moins<br>☐ De 501 à 1700 euros<br>☐ De 1701 à 2500 euros	pro_revenu prorevenu.

☐ De 2501 à 4000 euros<br>☐ De 4001 à 7000 euros<br>☐ Plus de 7000 euros<br>☐ Ne souhaite pas répondre	

PARTIE B. Vos perceptions sur l'épidémie en cours

(page 2/3)

**B1. Pensez-vous ou savez-vous si vous êtes actuellement atteint(e) par le COVID-19 ?**<br>☐ Oui, j'ai été testé(e) positif<br>☐ Oui, cela a été évoqué par un médecin mais je n'ai pas été testé ou j'ai été testé négatif<br>☐ C'est possible, je présente des symptômes (fièvre, toux, courbatures, fatigue intense, diarrhées, douleur thoracique, perte de l'odorat, gêne respiratoire)<br>☐ C'est peu probable, je ne me sens pas malade<br>☐ Je suis certain(e) de ne pas être atteint(e)<br>☐ Je ne sais pas	covid_atteint<br>atteint.
#si oui#<br>**B2. Pensez-vous avoir contracté le COVID-19 au cours de votre exercice professionnel ?**<br>☐ Oui, j'en suis certain.e<br>☐ Oui, c'est possible<br>☐ Non, je ne pense pas<br>☐ Je ne sais pas	covid_atteint_pro<br>attpro.
**B3. Avez-vous été hospitalisé.e pour la prise en charge du COVID-19 ?**<br>☐ Oui ☐ Non	covid_atteint_hosp<br>ouinon.
**B4. Avez-vous déjà eu le COVID-19 et êtes-vous actuellement rétabli.e ?**<br>☐ Oui avec un diagnostic confirmé (test positif)<br>☐ Oui mais sans qu'un test ait été réalisé<br>☐ Non	covid_sympt<br>ontest.

**B5. Y a-t-il, dans votre entourage ou votre famille, des personnes qui ont eu le COVID-19 ou des signes de maladie laissant à penser que c'était le COVID-19 ?**<br><br>☐ Oui ☐ Non<br><br>#si oui#<br><br>**B6. Un de vos proches a-t-il été hospitalisé pour la prise en charge du COVID-19 ?**<br><br>☐ Oui ☐ Non<br><br>**B7. Un de vos proches est-il décédé des suites du COVID-19 ?**<br><br>☐ Oui ☐ Non	`covid_fam ouinon.`<br><br>`covid_fam_hosp ouinon.`<br><br>`covid_fam_deces ouinon.`

B8. Dans le contexte de cette épidémie, quel est votre degré d'inquiétude à propos de :

	1 Pas du tout inquiet(ète)	2	3	4	5 Très inquiet(ète)	Non concerné(e)	
Votre santé (infection par le COVID-19)	❑	❑	❑	❑	❑	❑	`inq_sante inquiet.`
La santé de vos parents	❑	❑	❑	❑	❑	❑	`inq_parent inquiet.`
La santé de vos proches, de vos amis	❑	❑	❑	❑	❑	❑	`inq_amis inquiet.`
Votre situation financière	❑	❑	❑	❑	❑	❑	`inq_finance inquiet.`
De manquer de quelque chose (par ex., médicaments, pain, …)	❑	❑	❑	❑	❑	❑	`inq_manque inquiet.`
De manquer de soutien moral et affectif	❑	❑	❑	❑	❑	❑	`inq_soutien inquiet.`

B9. Vos revenus ont-ils changé avec l'épidémie ?

❑ Oui, ils ont diminué (perte de travail, de petit boulot, d'aides, de bourse, etc.)<br>❑ Non, ils sont restés inchangés	revenu_change change.
**B10.** *# Si profession cochée (autre que « pas de profession ») #* **Indiquez votre degré d'inquiétude par rapport à votre emploi (fin de CDD, licenciement,…) :**<br>*Donnez une note entre 0 (pas du tout inquiet(ète)) et 10 (très inquiet(ète)).*<br>0 1 2 3 4 5 6 7 8 9 10	inq_travail likert.
**B11. Indiquez votre degré d'inquiétude par rapport à vos études (report des examens, validation de l'année, report de la rentrée, report des soutenances…) :**<br>*Donnez une note entre 0 (pas du tout inquiet(ète)) et 10 (très inquiet(ète)).*<br>0 1 2 3 4 5 6 7 8 9 10	inq_etude likert.
**B12. Indiquez votre degré de satisfaction par rapport à l'aménagement de vos cours lors du confinement (en ligne, rendus écrits, etc.) :**<br>*Donnez une note entre 0 (pas du tout satisfait(e)) et 10 (très satisfait(e)).*<br>0 1 2 3 4 5 6 7 8 9 10	satisf_etude likert.
**B13. A quel degré situez-vous la dangerosité du COVID-19 pour vous-même ou vos proches ?**<br>*Donnez une note entre 0 (pas dangereux) et 10 (très dangereux).*<br>0 1 2 3 4 5 6 7 8 9 10	inq_danger likert.
**B14. Que pensez-vous de l'évolution de l'épidémie ? (Plusieurs réponses possibles)**<br>☐ L'épidémie va encore durer quelques semaines puis tout va rentrer dans l'ordre et nous reprendrons nos vies comme avant<br>☐ L'épidémie va évoluer par cycles pendant plusieurs mois et il pourra y avoir des phases de reconfinement<br>☐ Le coronavirus va rester parmi nous pendant plusieurs années et il faudra continuer à faire attention (gestes barrières, distanciation sociale)<br>☐ Dans l'année qui vient, le(s) vaccin(s) en cours de production va/vont nous permettre de nous débarrasser le virus	epidemie_evol1 coch.<br>epidemie_evol6 coch.<br>epidemie_evol7 coch.<br>epidemie_evol8 coch.<br>epidemie_evol5 coch.

☐ Sans opinion	
**Q. Selon vous, les affirmations suivantes sont-elles vraies ou fausses ?**	
**B15.** Le virus du COVID-19 a été conçu par l'Institut Pasteur en 2004<br>☐ Vrai ☐ Faux ☐ Je ne sais pas	fakenews9 vraifaux.
**B16.** Le virus est propagé par la 5G<br>☐ Vrai ☐ Faux ☐ Je ne sais pas	fakenews10 vraifaux.
**B17.** Ce sont presque toujours les personnes âgées de plus de 70 ans qui décèdent du COVID-19<br>☐ Vrai ☐ Faux ☐ Je ne sais pas	fakenews11 vraifaux.
**B18.** Les enfants et adultes jeunes (moins de 25 ans) font rarement des formes sévères du COVID-19<br>☐ Vrai ☐ Faux ☐ Je ne sais pas	fakenews12 vraifaux.
**B19.** Toutes les personnes contaminées par le Covid-19 développent des symptômes<br>☐ Vrai ☐ Faux ☐ Je ne sais pas	fakenews6 vraifaux.
**B20.** Boire des boissons très chaudes ou prendre des bains chauds permet d'éviter la contamination par le virus<br>☐ Vrai ☐ Faux ☐ Je ne sais pas	fakenews8 vraifaux.
**B21.** Le virus ne peut être transmis que par des personnes qui ont des symptômes comme de la fièvre et de la toux<br>☐ Vrai ☐ Faux ☐ Je ne sais pas	fakenews5 vraifaux.
**B22.** Le virus peut survivre un certain temps sur des surfaces inertes comme les poignées de porte ou les barres dans les transports en commun<br>☐ Vrai ☐ Faux ☐ Je ne sais pas	fakenews3 vraifaux.
**B23.** L'OMS ne préconise pas le port du masque pour le grand public	fakenews13 vraifaux.

☐ Vrai ☐ Faux ☐ Je ne sais pas<br><br>**B24.** Le premier confinement n'a servi à rien<br><br>☐ Vrai ☐ Faux ☐ Je ne sais pas<br><br>**B25.** La Suède n'a pas confiné et pour cela compte beaucoup moins de morts comparativement à la plupart des pays européens<br><br>☐ Vrai ☐ Faux ☐ Je ne sais pas	fakenews14 vraifaux.<br><br>fakenews15 vraifaux.

PARTIE C. Les consignes : les suivez-vous ? Qu'en pensez-vous ?

(page 4)

<table>
<tr><td>

C1. Selon vous, quel est le degré d'utilité de ce nouveau confinement ?

Donnez une note entre 0 (pas du tout utile) et 10 (très utile)

0 1 2 3 4 5 6 7 8 9 10

C2. Avez-vous téléchargé l'application TousAntiCovid ?

☐ Oui ☐ Non

- **#si oui#**

L'utilisez vous quand vous pensez être dans une situation à risque en contact avec d'autres personnes ?

☐ Oui
☐ Non
☐ Pas toujours

#si non#

Pour quelle raison principale vous ne l'avez pas téléchargée ?

☐ Ce n'est pas utile car peu de gens l'utilisent
☐ Je me méfie de la sécurité de mes données
☐ Pas assez de place et/ou de batterie sur mon téléphone
☐ Le fait d'utiliser tout le temps le Bluetooth est gênant
☐ Je n'ai pas compris comment ça fonctionne
☐ Avec le confinement, je ne sors pas beaucoup et je n'ai pas l'occasion de rencontrer des inconnus
☐ Les gestes barrières (masque, distance, lavage des mains) suffisent
☐ Je ne sais pas
☐ Autre

C3.

</td><td>

reconf_util likert.

appli ouinon.

appli_util ouintjr.

appli_non appraison.

</td></tr>
<tr><td>

C4. Diriez-vous que la réaction du gouvernement face à l'épidémie actuelle est :

</td><td></td></tr>
</table>

☐ Trop exagérée<br>☐ Plutôt exagérée<br>☐ Appropriée<br>☐ Plutôt insuffisante<br>☐ Bien trop insuffisante	reaction_gouv reac.
**C5. Diriez-vous que la réaction de la population face à l'épidémie actuelle est :**<br>☐ Trop exagérée<br>☐ Plutôt exagérée<br>☐ Appropriée<br>☐ Plutôt insuffisante<br>☐ Bien trop insuffisante	reaction_pop reac.
**C6. Trouvez-vous, à titre personnel, que les mesures sanitaires (distanciation physique, port de masque,…) sont faciles à respecter ?**<br>☐ Oui, tout à fait<br>☐ Oui, plutôt<br>☐ Non, plutôt pas<br>☐ Non, pas du tout	mesure_facil ech4.
**C7. Faites-vous confiance aux pouvoirs publics pour contrôler l'épidémie de COVID-19 ?**<br>*Donnez une note entre 0 (pas du tout confiance) et 10 (tout à fait confiance).*<br>0 1 2 3 4 5 6 7 8 9 10	conf_controle likert.
**C8. Faites-vous confiance aux pouvoirs publics pour vous informer sur le COVID-19 ?**<br>*Donnez une note entre 0 (pas du tout confiance) et 10 (tout à fait confiance).*<br>0 1 2 3 4 5 6 7 8 9 10	conf_info likert.
**Exprimez-vous : Qu'est-ce que vous pensez des mesures prises par les autorités publiques concernant l'épidémie ?**<br>*Nous vous proposons de vous exprimer librement en quelques mots ou quelques phrases.*	exprime_autorite $3000.

PARTIE D. Votre confinement

(Page 5/6)

**D1. Quel est votre lieu de confinement ?**<br>☐ Mon domicile habituel :<br>☐ un appartement<br>☐ une maison<br>☐ une chambre en cité universitaire ou foyer<br>☐ Autre<br>☐ Un autre endroit :<br>☐ un appartement<br>☐ une maison<br>☐ une chambre en cité universitaire ou foyer<br>☐ Autre<br><br>**D2. Quelle est la surface de votre lieu de confinement ?**<br>I_I_I_I m^2	conf_lieu conflieu.<br>conf_lieu_type lieutype.<br>conf_lieu_type lieutype.<br>conf_lieu_surf 4.
**Votre lieu de confinement dispose t'il :**<br>**D3.** D'un **balcon** ☐ Oui ☐ Non<br>**D4.** D'une **terrasse** ☐ Oui ☐ Non<br>**D5.** D'un **jardin** ☐ Oui ☐ Non	conf_balcon ouinon.<br>conf_terrase ouinon.<br>conf_jardin ouinon.
**D6. Nombre de personnes avec qui vous êtes confiné.e :**<br>☐ Je suis seul(e)<br>☐ 1 ☐ 2 ☐ 3 ☐ 4 ☐ 5 ☐ plus de 5<br><br>**D7.** # Si nombre de personnes ≥ 1, # **Ces personnes sont :** *(plusieurs réponses possibles)*<br>☐ Des membres de ma famille :<br>☐ Mon conjoint<br>☐ Des enfants (les miens ou ceux de mon cercle familial)<br>*Pour ajouter un enfant cliquer sur (+). Pour les enfants e moins de 1 an, renseigner 0.*<br>Age de l'enfant : ___<br>Age de l'enfant : ___<br>(+)<br>☐ Vos parents	conf_nbpers nbpers2.<br>conf_famille coch.<br>conf_conjoint coch.<br>conf_enfant coch.<br>enfant_age 2.<br>conf_pere coch.

☐ Mes frère(s)/ soeur(s)<br>☐ Autre(s) membre(s) de la famille élargie<br>☐ Des amis ou colocataires<br>D8.	conf_frere coch.<br>conf_autrefam coch.<br>conf_amis coch.
**D9. Quelles sont les conditions d'exercice de votre profession actuellement ? :**<br>☐ Vous travaillez comme auparavant sur votre lieu de travail à temps complet<br>☐ Vous télétravaillez à temps complet<br>☐ Vous avez une activité mixte entre télétravail et présence sur votre lieu de travail<br>☐ Vous avez diminué votre temps de travail ou ne travaillez que sur certains lieux<br>☐ Vous êtes au chômage technique ou partiel<br>☐ Vous êtes en congé forcé<br>☐ Vous êtes en arrêt de travail pour garde d'enfants<br>☐ Vous êtes en arrêt de travail pour maladie<br>☐ Vous avez perdu votre emploi du fait de la crise sanitaire<br>☐ Autre<br>**D10. Si vous devez travailler ou étudier de chez vous, comment évaluez-vous votre capacité à effectuer les tâches qui sont attendues de vous dans votre environnement actuel ?**<br>☐ Je suis loin de fournir le travail attendu et c'est un problème pour moi<br>☐ Je ne suis pas aussi performant(e)/concentré(e) que d'habitude mais j'arrive à travailler<br>☐ Je suis satisfait(e) de ce que j'arrive à faire<br>☐ Je travaille plutôt mieux que d'habitude<br>☐ *Je ne suis pas concerné(e) par cette question*	situation_act sitact.<br>eval_travail evaltr.

PARTIE E. Impact de l'épidémie

(page 7/8)

Q. Au cours des 2 dernières semaines, à quelle fréquence avez-vous été dérangé(e) par les problèmes suivants ?

		Presque jamais	Plusieurs jours	Plus de la moitié du temps	Presque tous les jours	
**E1.**	Peu d'intérêt ou de plaisir à faire les choses	❑	❑	❑	❑	pb_derang1 freq4.
**E2.**	Se sentir triste, déprimé(e) ou désespéré(e)	❑	❑	❑	❑	pb_derang2 freq4.
**E3.**	Difficultés à s'endormir ou à rester endormi(e), ou trop dormir	❑	❑	❑	❑	pb_derang3 freq4.
**E4.**	Se sentir fatigué(e) ou avoir peu d'énergie	❑	❑	❑	❑	pb_derang4 freq4.
**E5.**	Peu d'appétit ou trop manger	❑	❑	❑	❑	pb_derang5 freq4.
**E6.**	Mauvaise perception de vous-même — ou vous pensez que vous êtes un perdant ou que vous n'avez pas satisfait vos propres attentes ou celles de votre famille	❑	❑	❑	❑	pb_derang6 freq4.
**E7.**	Difficultés à se concentrer sur des choses telles que lire le journal ou regarder la télévision	❑	❑	❑	❑	pb_derang7 freq4.
**E8.**	Vous bougez ou parlez si lentement que les autres personnes ont pu le remarquer. Ou au contraire – vous êtes si agité(e) que vous bougez beaucoup plus que d'habitude	❑	❑	❑	❑	pb_derang8 freq4.
**E9.**	Vous avez pensé que vous seriez mieux mort(e) ou vous avez pensé à vous blesser d'une façon ou d'une autre	❑	❑	❑	❑	pb_derang9 freq4.

Q. Au cours des 2 dernières semaines, à quelle fréquence avez-vous été gêné(e) par les problèmes suivants ?

		Presque jamais	Plusieurs jours	Plus de la moitié du temps	Presque tous les jours	
E10.	Un sentiment de nervosité, d'anxiété ou de tension	❑	❑	❑	❑	
E11.	Une incapacité à arrêter de s'inquiéter ou à contrôler ses inquiétudes	❑	❑	❑	❑	pb_gene1 freq4.
E12.	Une inquiétude excessive à propos de différentes choses	❑	❑	❑	❑	pb_gene2 freq4.
E13.	Des difficultés à me détendre	❑	❑	❑	❑	pb_gene3 freq4.
E14.	Une agitation telle qu'il m'est difficile de tenir en place	❑	❑	❑	❑	pb_gene4 freq4.
E15.	Une tendance à être facilement contrarié(e) ou irritable	❑	❑	❑	❑	pb_gene5 freq4.
E16.	Un sentiment de peur comme si quelque chose de terrible risquait de se produire	❑	❑	❑	❑	pb_gene6 freq4.
						pb_gene7 freq4.

PARTIE F. Votre avis sur traitements et vaccins

(page 9)

**F1. Si vous étiez atteint.e du COVID-19, seriez-vous prêt.e à prendre un traitement dont l'efficacité n'est pas certaine ?**<br>☐ Oui, même s'il y a un risque à prendre ce traitement<br>☐ Oui, mais à condition que les risques liés au traitement soient faibles<br>☐ Oui, mais à condition qu'il n'y ait absolument aucun risque lié au traitement, même faible<br>☐ Non, jamais<br>☐ Je ne sais pas	trait_prendre prendre.

**F2. Avez-vous confiance dans le fait qu'un traitement efficace contre le COVID-19 sera trouvé ?**<br>☐ Oui, dans quelques mois<br>☐ Oui, dans quelques années<br>☐ Non<br>☐ Je ne sais pas<br><br>**F3. Seriez-vous prêt.e à vous faire vacciner contre le COVID-19 même si le vaccin n'a pas encore fait complètement la preuve de son efficacité ?**<br>☐ Oui, même s'il y a un risque lié au vaccin<br>☐ Oui, mais à condition que le risque lié au vaccin soit faible<br>☐ Oui, mais à condition qu'il n'y ait absolument aucun risque lié au vaccin, même faible<br>☐ Oui, dans le cadre d'un essai clinique (si un vaccin n'est pas encore validé)<br>☐ Non, jamais<br>☐ Je ne sais pas<br><br>**F4. Si un vaccin contre le COVID-19 était produit dans 12 mois, alors que l'épidémie actuelle serait passée mais qu'il y aurait un risque qu'elle revienne chaque année comme la grippe, iriez-vous vous faire vacciner ?**<br>1 Pas du tout ❑ · 2 ❑ · 3 ❑ · 4 ❑ · 5 Certainement ❑	trait_covid trait.<br><br>vaccovid vaccovid.<br><br>vaccovid_an cert.
**F5. Vous faites-vous vacciner régulièrement contre la grippe ?**<br>☐ Oui ☐ Non<br><br>**F6. Etes-vous à jour de vos vaccinations ?**<br>☐ Oui ☐ Non ☐ Je ne sais pas	vacgrippe ouinon<br><br>vacautre onnsp.
**Concernant la vaccination en général, à quel point êtes-vous d'accord avec les propositions suivantes ?**	

	Entiè-rement d'accord	Plutôt d'accord	Plutôt pas d'accord	Pas d'accord	Sans opinion	
**F7.** La vaccination peut provoquer de graves effets secondaires	❑	❑	❑	❑	❑	vac_effsec accord5.
**F8.** La vaccination n'a pas beaucoup d'intérêt, car il y a très peu de risque d'être infecté.e	❑	❑	❑	❑	❑	vac_nointeret accord5.
**F9.** Il n'est pas nécessaire d'être vacciné.e car beaucoup de gens le sont autour de nous	❑	❑	❑	❑	❑	vac_nonec accord5.
**F10.** Si un vaccin n'est pas obligatoire c'est qu'il n'est pas si important	❑	❑	❑	❑	❑	vac_nonoblig accord5.
**F11.** Les industries pharmaceutiques incitent à se faire vacciner pour augmenter leurs bénéfices	❑	❑	❑	❑	❑	vac_benef accord5.
**F12.** Se faire vacciner soi-même a un impact sur la santé des autres	❑	❑	❑	❑	❑	vaccin_impact accord5.

Concernant la recherche d'informations sur la vaccination, à quel point êtes-vous d'accord avec les propositions suivantes ?

	Entiè-rement d'accord	Plutôt d'accord	Plutôt pas d'accord	Pas d'accord	Sans opinion	
**F13.** Je pense qu'il est facile de se renseigner sur la	❑	❑	❑	❑	❑	decvac_internet accord5.

vaccination sur Internet						
**F14.** Je trouve que les informations concernant la vaccination sur les réseaux sociaux et les forums sont compréhensibles	❑	❑	❑	❑	❑	decvac_rs accord5.
**F15.** Je trouve que les informations concernant la vaccination sur les sites gouvernementaux sont compréhensibles	❑	❑	❑	❑	❑	decvac_gouv accord5.
**F16.** Je suis capable de reconnaître les fake news sur le thème de la vaccination	❑	❑	❑	❑	❑	decvac_fakenews accord5.
**F17.** J'ai confiance dans les informations fournies par les sites gouvernementaux	❑	❑	❑	❑	❑	decvac_gouv_conf accord5.
**F18.** Je trouve que les informations sur la vaccination sur les réseaux sociaux sont valides	❑	❑	❑	❑	❑	decvac_rs_valide accord5.
**F19.** Lorsque je prends connaissance d'une information sur la vaccination en ligne, je croise plusieurs sources afin de vérifier sa validité	❑	❑	❑	❑	❑	decvac_source accord5.
**F20.** Je pense que les informations trouvées en ligne peuvent influencer mon choix de me faire vacciner	❑	❑	❑	❑	❑	decvac_influe accord5.

**F21.** **Si je ne pense pas avoir assez de connaissances sur la vaccination et sur ses risques, je préfère ne pas me faire vacciner**	❑	❑	❑	❑	❑	decvac_nonconnu accord5.
**F22.** **Si je ne pense pas avoir assez de connaissances sur la vaccination et sur ses risques, j'interroge les professionnels de santé que je connais**	❑	❑	❑	❑	❑	decvac_prof accord5.

**Exprimez-vous : Que pensez-vous de la vaccination comme mesure pour prévenir les maladies comme le COVID-19 ?**<br>*Nous vous proposons de vous exprimer librement en quelques mots ou quelques phrases.*	exprime_vaccin $3000.

PARTIE G. Votre profil médical

(page 10/11)

**G1. Avant le début de l'épidémie, comment caractériseriez-vous votre santé ?**<br>❒ Très bonne<br>❒ Bonne<br>❒ Moyenne<br>❒ Mauvaise<br>❒ Très mauvaise	sante_avant evalsante.
**G2. Avant le début de l'épidémie, quelle note donneriez-vous à votre qualité de vie ?**<br>*(0 pour la pire qualité de vie possible et 10 pour la meilleure qualité de vie possible)*	qdv_avant likert.

0 1 2 3 4 5 6 7 8 9 10<br><br>**G3. Avant le début de l'épidémie, diriez-vous que vous êtiez souvent inquiet.ète ou stressé.e ?**<br>*Donnez une note entre 0 (no stress) et 10 (très stressé(e)).*<br><br>0 1 2 3 4 5 6 7 8 9 10<br><br>**G4. Tendez-vous à rebondir rapidement après des moments difficiles ?**<br>☐ Oui, tout à fait<br>☐ Plutôt oui<br>☐ Neutre<br>☐ Non, pas vraiment<br>☐ Non, pas du tout	stress_normal likert.<br><br>rebondir onneutre.
**G5. Au cours de votre vie, un médecin a-t-il déjà diagnostiqué chez vous un ou plusieurs problèmes de santé suivants ?**<br>Maladie cardio-vasculaire (infarctus du myocarde, AVC,…)<br>☐ Oui ☐ Non ☐ Ne sait pas<br>*# Si oui, #* êtes-vous actuellement traité ? ☐ Oui ☐ Non<br><br>Hypertension artérielle<br>☐ Oui ☐ Non ☐ Ne sait pas<br>*# Si oui, #* êtes-vous actuellement traité ? ☐ Oui ☐ Non<br><br>Diabète<br>☐ Oui ☐ Non ☐ Ne sait pas<br>*# Si oui, #* êtes-vous actuellement traité ? ☐ Oui ☐ Non<br><br>Maladie digestive chronique (ex. Crohn, ulcère)<br>☐ Oui ☐ Non ☐ Ne sait pas<br>*# Si oui, #* êtes-vous actuellement traité ? ☐ Oui ☐ Non<br><br>Cancer<br>☐ Oui ☐ Non ☐ Ne sait pas	atcd_cardio onnsp.<br>atcd_cardio_tt ouinon.<br><br>atcd_hta onnsp.<br>atcd_hta_tt ouinon.<br><br>atcd_diab onnsp.<br>atcd_diab_tt ouinon.<br><br>atcd_digest onnsp.<br>atcd_digest_tt ouinon.<br><br>atcd_cancer onnsp.<br>atcd_cancer_tt ouinon.

# Si oui, # êtes-vous actuellement traité ? ☐ Oui ☐ Non	
Asthme ou autre problème respiratoire<br>☐ Oui ☐ Non ☐ Ne sait pas<br>***# Si oui, #*** êtes-vous actuellement traité ? ☐ Oui ☐ Non	atcd_respi onnsp.<br>atcd_respi_tt ouinon.
Un problème de santé mentale (dépression, trouble bipolaire, anxiété généralisée…)<br>☐ Oui ☐ Non ☐ Ne sait pas<br>***# Si oui, #*** êtes-vous actuellement traité ? ☐ Oui ☐ Non	atcd_psy onnsp.<br>atcd_psy_tt ouinon.
Autre(s) maladie affectant le système immunitaire (comme HIV, lymphopathie maligne, …)<br>☐ Oui ☐ Non ☐ Ne sait pas<br>***# Si oui, #*** êtes-vous actuellement traité ? ☐ Oui ☐ Non	atcd_immun onnsp.<br>atcd_immun_tt ouinon.
Autre(s) problème(s) de santé<br>☐ Oui ☐ Non ☐ Ne sait pas<br>***# Si oui, #*** êtes-vous actuellement traité ? ☐ Oui ☐ Non	atcd_autre onnsp.<br>atcd_autre_tt ouinon.

**Les questions suivantes concernent votre consommation de tabac et d'alcool <u>au cours de l'année écoulée</u> et pas seulement les dernières semaines.**	
**G6. Concernant votre consommation de tabac au cours de l'année écoulée, êtes-vous :**<br>☐ Fumeur.se régulier.e de tabac (au moins une cigarette par jour)<br>☐ Fumeur.se occasionnel.le de tabac (moins d'une cigarette par jour)<br>☐ Ex-fumeur.se régulier.e de tabac (au moins une cigarette par jour)<br>☐ Non-fumeur.se (vous n'avez jamais fumé une cigarette par jour)	avant_tabac fumeur.
**G7. Au cours de l'année écoulée, avez-vous consommé du cannabis ?**<br>☐ Non<br>☐ Oui, ponctuellement<br>☐ Oui, régulièrement mais moins de 4 fois par semaine<br>☐ Oui, au moins 4 fois par semaine<br>☐ Oui, tous les jours	avant_cannabis cannab.
**G8. Combien de fois vous est-il arrivé de consommer de l'alcool au cours de**	avant_alcool

**l'année écoulée ?**<br><br>☐ Jamais<br>☐ Moins d'une fois par mois<br>☐ Une fois par mois<br>☐ 2 à 4 fois par mois<br>☐ 2 à 3 fois par semaine<br>☐ 4 fois ou plus par semaine<br><br>**G9. #Si alcool au moins une fois par mois, # Combien de verres standard buvez-vous au cours d'une journée ordinaire où vous buvez de l'alcool ?**<br><br>*Un verre d'alcool correspond à un ballon de vin (10 cl), un demi de bière (25 cl) ou un verre d'alcool fort (4 cl).*<br><br>☐ Un ou deux<br>☐ Trois ou quatre<br>☐ Cinq ou six<br>☐ Sept à neuf<br>☐ Dix ou plus<br><br>**G10. #Si alcool au moins une fois par mois, # Au cours d'une même occasion, combien de fois vous est-il arrivé de boire 6 verres standard ou plus (au cours de l'année écoulée) ?**<br><br>☐ Jamais<br>☐ Moins de 1 fois par mois<br>☐ 1 fois par mois<br>☐ Une fois par semaine<br>☐ Chaque jour ou presque	`freqmois.`<br><br>`avant_verre nbverre.`<br><br>`avant_6verres freqverrres.`

PARTIE H. Vos activités

(page 12)

**H1. Au cours des 7 derniers jours, avez-vous porté un masque quand vous êtes allé.e dehors (faire les courses par exemple) ?**<br>☐ Oui, tout le temps<br>☐ Oui, parfois<br>☐ Non, car je n'ai pas de masque mais sinon j'en porterai un<br>☐ Non, car je ne crois pas que ce soit important<br>☐ Non concerné.e	`masque_dehors masque.`
**H2. Actuellement, sur une échelle de 0 à 10 (0=pas du tout, 10=totalement), à quel point vous sentez-vous seul.e ?**<br>0 1 2 3 4 5 6 7 8 9 10	`seul_eva likert.`

Q. A quelle fréquence avez-vous des interactions sociales avec votre famille ou des amis ?

		Jamais	Moins d'une fois par semaine	Une fois par semaine	Plusieurs fois par semaine	Tous les jours	
H3.	**Directement en face à face**	❑	❑	❑	❑	❑	`interFreq_ftf freqsem5.`
H4.	**Au téléphone**	❑	❑	❑	❑	❑	`interFreq_tel freqsem5.`
H5.	**Par SMS**	❑	❑	❑	❑	❑	`interFreq_sms freqsem5.`
H6.	**Sur les réseaux sociaux**	❑	❑	❑	❑	❑	`interFreq_rs freqsem5.`

PARTIE I. Vos perceptions sur la santé

(page 13)

Veuillez indiquer dans quelle mesure vous êtes d'accord ou non avec chacune des affirmations suivantes.

		Pas du tout d'accord	Pas d'accord	D'accord	Tout à fait d'accord	
**I1.**	Je compare les informations sur la santé qui viennent de différentes sources	❑	❑	❑	❑	infosante_compare accord.
**I2.**	Quand je découvre une nouvelle information sur la santé, je vérifie si elle est vraie ou non	❑	❑	❑	❑	infosante_verifie accord.
**I3.**	Je compare toujours les informations sur la santé à partir de différentes sources et je décide ce qui est le mieux pour moi	❑	❑	❑	❑	infosante_sources accord.
**I4.**	Je sais déterminer si une information sur la santé est adaptée à ma situation ou pas	❑	❑	❑	❑	infosante_determine accord.
**I5.**	J'interroge les professionnels de santé sur la qualité des informations que je trouve	❑	❑	❑	❑	infosante_pro accord.

PARTIE J. Vous gardez le moral ?

(page 14/15)

**J1. Comment jugez-vous actuellement votre moral sur une échelle de 0 à 10 ?**<br>*Donnez une note entre 0 (très mauvais) et 10 (très bon).*<br>0 1 2 3 4 5 6 7 8 9 10<br><br>**J2. À quel point êtes-vous inquiet(ète) ou stressé(e) en ce moment sur une échelle de 0 à 10 ?**<br>*Donnez une note entre 0 (no stress) et 10 (très stressé(e)).*<br>0 1 2 3 4 5 6 7 8 9 10	moral_act likert.<br><br>stress_act likert.
**J3. Au cours des 7 derniers jours, avez-vous mis en place des routines pour avoir un rythme de vie régulier ? (ex. dîner toujours à la même heure, lire tous les soirs avant de dormir, appeler tous les jours vos proches)**<br>❒ Oui et j'arrive à m'y tenir<br>❒ Oui, mais je ne les respecte pas tout le temps<br>❒ Non, mon rythme de vie en ce moment est plutôt déréglé<br>❒ Non, je n'en ai pas besoin<br><br>**J4. Au cours des 7 derniers jours, comment avez-vous dormi ?**<br>❒ Bien<br>❒ Plutôt bien<br>❒ Ni bien ni mal<br>❒ Plutôt mal<br>❒ Mal<br><br>**J5. Au cours des 7 derniers jours, avez-vous eu des difficultés d'endormissement et/ou de maintien de votre sommeil (réveils nocturnes) ?**<br>❒ Jamais ou moins d'1 fois par mois<br>❒ Moins d'1 fois par semaine<br>❒ 1 à 2 jours par semaine<br>❒ 3 à 5 jours par semaine<br>❒ Tous les jours ou presque<br><br>**J6. Au cours des 7 derniers jours, vous êtes-vous senti extrêmement somnolent durant la journée ?**<br>❒ Jamais ou moins d'1 fois par mois<br>❒ Moins d'1 fois par semaine	routine rout.<br><br>dormi_qlt qual.<br><br>dormi_diff freqdorm.<br><br>dormi_somn

❒ 1 à 2 jours par semaine<br>❒ 3 à 5 jours par semaine<br>❒ Tous les jours ou presque	freqdorm.
**J7. Au cours des 7 derniers jours, quelle a été votre heure de lever en moyenne ?**<br>|__| __| : |__| __|	dormi_lever hhmm.
**J8. Au cours des 7 derniers jours, quelle a été votre heure de coucher en moyenne ?**<br>|__| __| : |__| __|	dormi_coucher hhmm.
**J9. Au cours des 7 derniers jours, comment était votre rythme de lever et de coucher ?**<br>❒ Très irrégulier<br>❒ Plutôt irrégulier<br>❒ Ni régulier ni irrégulier<br>❒ Plutôt régulier<br>❒ Très régulier	dormi_rythme rythme.

Q. <u>Au cours des 7 derniers jours</u>, comment ont évolué vos consommations ?

		Je n'en consomme pas habituel-lement	Iden-tique	Diminution ou arrêt sans manque	Dimi-nution ou arrêt avec manque	Augmen-tation modérée	Augmen-tation difficile à contrôler	
**J10.**	**Café, thé et/ou boissons énergisantes**	❑	❑	❑	❑	❑	❑	evolconso_cafe evolconso.
**J11.**	**Aliments gras, sucrés et/ou salés**	❑	❑	❑	❑	❑	❑	evolconso_sucre evolconso.
**J12.**	**Tabac**	❑	❑	❑	❑	❑	❑	evolconso_tabac evolconso.
**J13.**	**Cigarette électronique**	❑	❑	❑	❑	❑	❑	evolconso_ecig evolconso.
**J14.**	**Alcool**	❑	❑	❑	❑	❑	❑	evolconso_alcool evolconso.
**J15.**	**Cannabis**	❑	❑	❑	❑	❑	❑	evolconso_cannabis evolconso.
**J16.**	**Autres drogues**	❑	❑	❑	❑	❑	❑	evolconso_drogu

								Variable
	**(ecstasy…)**							es evolconso.
**J17.**	**Médicaments pour dormir**	❑	❑	❑	❑	❑	❑	evolconso_medic evolconso.
**J18.**	**Anxiolytiques (alprazolam…)**	❑	❑	❑	❑	❑	❑	evolconso_anxio evolconso.
**J19.**	**Ecrans** (TV, smartphone, tablette, ordinateur…)	❑	❑	❑	❑	❑	❑	evolconso_ecran evolconso.

J20.#Si alcool au moins 6 verres <> Jamais, # Si cela vous arrive de boire 6 verres d'alcool en une seule occasion et sur un temps court : cela vous arrive-t-il plus fréquemment depuis le confinement ?

alcool_conso alcool.

☐ Non, la fréquence de ces épisodes n'a pas changé

☐ Oui, la fréquence a diminué/ j'ai arrêté

☐ Oui, la fréquence a un peu augmenté

☐ Oui, elle a un beaucoup augmenté

☐ *Je ne suis pas concerné(e)*

J21. Au cours des 7 derniers jours, vous est-il arrivé de penser à vous suicider (d'avoir des idées suicidaires) ?

suicide onqqf.

❒ Non, jamais
❒ Oui, quelquefois
❒ Oui, à de multiples reprises

# Si oui, #

J22. Avez-vous pensé à la manière dont vous vous y prendriez ?

suic_maniere suicman.

☐ Non

☐ Oui, mais pas dans le détail

☐ Oui, j'ai réfléchi à un scénario précis

J23. Vous est-il arrivé de penser à vous suicider (d'avoir des idées suicidaires) au cours des 12 derniers mois qui ont précédé le début de l'épidémie ?

suic_avant onqqf.

☐ Non, jamais

☐ Oui, quelquefois

☐ Oui, à de multiples reprises

Quelle note donneriez-vous à votre qualité de vie <u>au cours des 7 derniers jours</u> ?

(0 pour la pire qualité de vie possible et 10 pour la meilleure qualité de vie possible)<br><br>0 1 2 3 4 5 6 7 8 9 10	`qdv_act likert.`

Questionnaire SUIVI MENSUEL

Date de remplissage du questionnaire : |__|__|/ |__|__|/ 2020
Jour Mois

(Date système remplie de manière automatique)

Partie A. Vos perceptions sur l'épidémie en cours

**M1. Selon vous, quel est le degré d'utilité de ce nouveau confinement ?**<br>*Donnez une note entre 0 (pas du tout utile) et 10 (très utile)*<br>0 1 2 3 4 5 6 7 8 9 10	reconf_util likert.
**M2. Avez-vous téléchargé l'application TousAntiCovid ?**<br>☐ Oui ☐ Non	appli ouinon.
- **#si oui#**<br>**L'utilisez vous quand vous pensez être dans une situation à risque en contact avec d'autres personnes ?**<br>☐ Oui<br>☐ Non<br>☐ Pas toujours	appli_util ouintjr.
**#si non#**<br>**Pour quelle raison principale vous ne l'avez pas téléchargée ?**<br>☐ Ce n'est pas utile car peu de gens l'utilisent<br>☐ Je me méfie de la sécurité de mes données<br>☐ Pas assez de place et/ou de batterie sur mon téléphone<br>☐ Le fait d'utiliser tout le temps le Bluetooth est gênant<br>☐ Je n'ai pas compris comment ça fonctionne<br>☐ Avec le confinement, je ne sors pas beaucoup et je n'ai pas l'occasion de rencontrer des inconnus<br>☐ Les gestes barrières (masque, distance, lavage des mains) suffisent<br>☐ Je ne sais pas<br>☐ Autre	appli_non appraison.

M3. Pensez-vous avoir contracté la COVID-19 ?

- ❒ Oui, j'ai été testé(e) positif
- ❒ Oui, cela a été évoqué par un médecin mais je n'ai pas été testé ou j'ai été testé négatif
- ❒ C'est possible, je présente des symptômes (fièvre, toux, courbatures, fatigue intense, diarrhées, douleur thoracique, perte de l'odorat, gêne respiratoire)
- ❒ C'est peu probable, je ne me sens pas malade
- ❒ Je suis certain(e) de ne pas être atteint(e)
- ❒ Je ne sais pas

`covid_atteint atteint.`

# Si Oui, j'ai été testé(e) positif, ajouter M3-M5 #

M4. Quand ?

|__| / |____| (mm/aaaa)

`covid_atteint_mois 2.`
`covid_atteint_annee 4.`

M5. Pensez-vous avoir contracté la COVID-19 au cours de votre exercice professionnel ?

- ❒ Oui, j'en suis certain.e
- ❒ Oui, c'est possible
- ❒ Non, je ne pense pas
- ❒ Je ne sais pas

`covid_atteint_pro attpro.`

M6. Avez-vous été hospitalisé.e pour avoir contracté la COVID-19 ?

- ❒ Oui
- ❒ Non

`covid_atteint_hosp ouinon.`

M7. Y a-t-il, dans votre entourage ou votre famille, des personnes qui ont eu la COVID-19 ou des signes de maladie laissant à penser que c'était la COVID-19 ?

- ❒ Oui
- ❒ Non

`covid_fam ouinon.`

# Si Oui, → M5 et M6 #

M8. Un de vos proches a-t-il été hospitalisé pour avoir contracté la COVID-19 ?

- ❒ Oui
- ❒ Non

`covid_fam_hosp ouinon.`

M9. Un de vos proches est-il décédé des suites de la COVID-19 ?

- ❒ Oui
- ❒ Non

`covid_fam_deces ouinon.`

M10. Dans le contexte de cette épidémie, quel est votre degré d'inquiétude à propos de :

	1<br>Pas du tout inquiet(ète)	2	3	4	5<br>Très inquiet(ète)	Non concerné(e)
Votre santé	❑	❑	❑	❑	❑	❑

(infection par la COVID-19)							inq_sante inquiet.
La santé de vos parents	❑	❑	❑	❑	❑	❑	inq_parent inquiet.
La santé d'un de vos proches, de vos amis	❑	❑	❑	❑	❑	❑	inq_amis inquiet.
Votre situation financière	❑	❑	❑	❑	❑	❑	inq_finance inquiet.

M11. Que pensez-vous de l'évolution de l'épidémie ? (Plusieurs réponses possibles)

- ❒ L'épidémie va encore durer quelques semaines puis tout va rentrer dans l'ordre et nous reprendrons nos vies comme avant — epidemie_evol1 coch.
- ❒ L'épidémie va évoluer par cycles pendant plusieurs mois et il pourra y avoir des phases de reconfinement — epidemie_evol6 coch.
- ❒ Le coronavirus va rester parmi nous pendant plusieurs années et il faudra continuer à faire attention (gestes barrières, distanciation sociale) — epidemie_evol7 coch.
- ❒ Dans l'année qui vient, le(s) vaccin(s) en cours de production va/vont nous permettre de nous débarrasser le virus — epidemie_evol8 coch.
- ❒ Sans opinion — epidemie_evol5 coch.

Partie B. Votre quotidien face à l'épidémie

**M12. Quelles sont les conditions d'exercice de votre profession actuellement ??**<br>☐ Vous travaillez sur votre lieu de travail à temps complet<br>☐ Vous travaillez sur votre lieu de travail à temps partiel<br>☐ Vous télétravaillez à temps complet<br>☐ Vous avez une activité mixte entre télétravail et présence sur votre lieu de travail<br>☐ Vous êtes au chômage technique ou partiel<br>☐ Vous êtes en arrêt de travail pour garde d'enfants<br>☐ Vous êtes en arrêt de travail pour maladie<br>☐ Vous avez perdu votre emploi du fait de la crise sanitaire<br>☐ Vous êtes à la retraite<br>☐ Autre	evol_prof evolpro.
**M13.** # Si profession cochée (autre que « pas de profession ») # **Indiquez votre degré d'inquiétude par rapport à votre emploi (fin de CDD, licenciement,…) :**<br>*Donnez une note entre 0 (pas du tout inquiet.ète) et 10 (très inquiet.ète).*<br>*0 1 2 3 4 5 6 7 8 9 10*	inq_travail likert.
**M14. Indiquez votre degré d'inquiétude par rapport à vos études (rentrée universitaire, validation de l'année, …) :**<br>*Donnez une note entre 0 (pas du tout inquiet(ète)) et 10 (très inquiet(ète)).*<br>*0 1 2 3 4 5 6 7 8 9 10*	inq_etude likert.
**M15. Indiquez votre degré de satisfaction par rapport à l'aménagement de vos cours lors du confinement (en ligne, rendus écrits, etc.) :**<br>*Donnez une note entre 0 (pas du tout satisfait(e)) et 10 (très satisfait(e)).*<br>0 1 2 3 4 5 6 7 8 9 10<br>**M16.**	satisf_etude likert.
**D11. Si vous devez travailler ou étudier de chez vous, comment évaluez-vous votre capacité à effectuer les tâches qui sont attendues de vous dans votre environnement actuel ?**<br>☐ Je suis loin de fournir le travail attendu et c'est un problème pour moi<br>☐ Je ne suis pas aussi performant(e)/concentré(e) que d'habitude mais j'arrive à travailler<br>☐ Je suis satisfait(e) de ce que j'arrive à faire<br>☐ Je travaille plutôt mieux que d'habitude<br>☐ *Je ne suis pas concerné(e) par cette question*<br>**M17.**	eval_travail evaltr.
**M18.** **<u>Au cours des 7 derniers jours</u>, avez-vous pratiqué une activité physique ?**<br>❒ Non	

❒ Oui, mais pas tous les jours<br>❒ Oui, tous les jours	sport tsjours.
**M19.** **<u>Au cours des 7 derniers jours</u>, sur une échelle de 0 à 10 (0=pas du tout, 10=totalement), à quel point vous sentez-vous seul.e ?**<br><br>0 1 2 3 4 5 6 7 8 9 10	seul_eva likert.

Partie C. Votre moral

**M20.** **À quel point êtes-vous inquiet(ète) ou stressé(e) en ce moment sur une échelle de 0 à 10 ?**<br>*Donnez une note entre 0 (no stress) et 10 (très stressé(e)).*<br>0 1 2 3 4 5 6 7 8 9 10	stress_act likert.
**M21.** **<u>Au cours des 7 derniers jours</u>, comment avez-vous dormi ?**<br>❒ Bien<br>❒ Plutôt bien<br>❒ Ni bien ni mal<br>❒ Plutôt mal<br>❒ Mal	dormi_qlt qual.
**M22.** **<u>Au cours des 7 derniers jours,</u> avez-vous eu des difficultés d'endormissement et/ou de maintien de votre sommeil (réveils nocturnes) ?**<br>❒ Jamais ou moins d'1 fois par mois<br>❒ Moins d'1 fois par semaine<br>❒ 1 à 2 jours par semaine<br>❒ 3 à 5 jours par semaine<br>❒ Tous les jours ou presque	dormi_diff freqdorm.
**M23.** **Si cela vous arrive de boire 6 verres d'alcool en une seule occasion et sur un temps court : cela vous est-il arrivé plus fréquemment <u>au cours des 7 derniers jours</u>?**<br>☐ Non, la fréquence de ces épisodes n'a pas changé<br>☐ Oui, la fréquence a un peu augmenté<br>☐ Oui, elle a un beaucoup augmenté<br>☐ *Je ne suis pas concerné*	alcool_conso alcool.

Nous vous proposons de répondre maintenant à plusieurs questions sur votre santé psychique. Si vous avez besoin de soutien psychologique, ou si certaines questions vous ont déstabilisé.e ou perturbé.e, n'hésitez pas à faire appel à l'un des nombreux dispositifs d'aide à distance dont vous trouverez les coordonnées dans l'onglet « Contact ».

<u>Au cours des 2 dernières semaines</u>, à quelle fréquence avez-vous été dérangé.e par les problèmes suivants ?

		Presque jamais	Plusieurs jours durant ces 2 dernières semaines	Plus de la moitié du temps	Presque tous les jours
**M24.**	Peu d'intérêt ou de plaisir à faire les choses	❑	❑	❑	❑
**M25.**	Se sentir triste, déprimé.e ou désespéré.e	❑	❑	❑	❑
**M26.**	Difficultés à s'endormir ou à rester endormi.e, ou trop dormir	❑	❑	❑	❑
**M27.**	Se sentir fatigué.e ou avoir peu d'énergie	❑	❑	❑	❑
**M28.**	Peu d'appétit ou trop manger	❑	❑	❑	❑
**M29.**	Mauvaise perception de vous-même - ou vous pensez que vous êtes un.e perdant.e ou que vous n'avez pas satisfait vos propres attentes ou celles de votre famille	❑	❑	❑	❑
**M30.**	Difficultés à se concentrer sur des choses telles que lire le journal ou regarder la télévision	❑	❑	❑	❑

```
pb_derang1 freq4.
pb_derang2 freq4.
pb_derang3 freq4.
pb_derang4 freq4.
pb_derang5 freq4.
pb_derang6 freq4.
pb_derang7 freq4.
```

M31.	Vous bougez ou parlez si lentement que les autres personnes ont pu le remarquer. Ou au contraire - vous êtes si agité.e que vous bougez beaucoup plus que d'habitude	❑	❑	❑	❑	pb_derang8 freq4.
M32.	Vous avez pensé que vous seriez mieux mort.e ou vous avez pensé à vous blesser d'une façon ou d'une autre	❑	❑	❑	❑	pb_derang9 freq4.

Au cours des 2 dernières semaines, à quelle fréquence avez-vous été gêné.e par les problèmes suivants ?

		Presque jamais	Plusieurs jours au cours de ces 2 dernières semaines	Plus de la moitié du temps	Presque tous les jours	
M33.	Un sentiment de nervosité, d'anxiété ou de tension	❑	❑	❑	❑	pb_gene1 freq4.
M34.	Une incapacité à arrêter de s'inquiéter ou à contrôler ses inquiétudes	❑	❑	❑	❑	pb_gene2 freq4.
M35.	Une inquiétude excessive à propos de différentes choses	❑	❑	❑	❑	pb_gene3 freq4.
M36.	Des difficultés à me détendre	❑	❑	❑	❑	pb_gene4 freq4.
M37.	Une agitation telle qu'il m'est difficile de tenir en place	❑	❑	❑	❑	pb_gene5 freq4.
M38.	Une tendance à être facilement contrarié.e	❑	❑	❑	❑	

	ou irritable					pb_gene6 freq4.
**M39.**	Un sentiment de peur comme si quelque chose de terrible risquait de se produire	❑	❑	❑	❑	pb_gene7 freq4.

M40. **<u>Au cours des 2 dernières semaines</u>, vous est-il arrivé de penser à vous suicider (d'avoir des idées suicidaires) ?**

❐ Non, jamais
❐ Oui, quelquefois
❐ Oui, à de multiples reprises

suicide onqqf.

# Si oui, quelquefois ou Oui, à de multiples reprises, → M35 ET M36 #

M41. **Avez-vous pensé à la manière dont vous vous y prendriez ?**

❐ Non
❐ Oui, mais pas dans le détail
❐ Oui, j'ai réfléchi à un scénario précis

suic_maniere suicman.

M42. **Vous est-il arrivé de penser à vous suicider (d'avoir des idées suicidaires) au cours des 12 derniers mois ?**

❐ Non, jamais
❐ Oui, quelquefois
❐ Oui, à de multiples reprises

suic_annee onqqf.

M43. **Actuellement, quelle note donneriez-vous à votre qualité de vie ?**
(0 pour la pire qualité de vie possible et 10 pour la meilleure qualité de vie possible)

qdv_act likert.

0 1 2 3 4 5 6 7 8 9 10

Partie D. Votre confinement

**D1. Quel est votre lieu de confinement ?**<br>☐ Mon domicile habituel :<br>☐ un appartement<br>☐ une maison<br>☐ une chambre en cité universitaire ou foyer<br>☐ Autre<br>☐ Un autre endroit :<br>☐ un appartement<br>☐ une maison<br>☐ une chambre en cité universitaire ou foyer<br>☐ Autre<br>**D2. Quelle est la surface de votre lieu de confinement ?**<br>I_I_I_I m^2	conf_lieu conflieu.<br>conf_lieu_type lieutype.<br>conf_lieu_type lieutype.<br>conf_lieu_surf 4.
**Votre lieu de confinement dispose t'il :**<br>**D3.** D'un **balcon** ☐ Oui ☐ Non<br>**D4.** D'une **terrasse** ☐ Oui ☐ Non<br>**D5.** D'un **jardin** ☐ Oui ☐ Non	conf_balcon ouinon.<br>conf_terrase ouinon.<br>conf_jardin ouinon.
**D6. Nombre de personnes avec qui vous êtes confiné.e :**<br>☐ Je suis seul(e)<br>☐ 1 ☐ 2 ☐ 3 ☐ 4 ☐ 5 ☐ plus de 5<br>**D7.** # Si nombre de personnes ≥ 1, # **Ces personnes sont :** *(plusieurs réponses possibles)*<br>☐ Des membres de ma famille :<br>☐ Mon conjoint<br>☐ Des enfants (les miens ou ceux de mon cercle familial)<br>*Pour ajouter un enfant cliquer sur (+). Pour les enfants e moins de 1 an, renseigner 0.*<br>Age de l'enfant : ___<br>Age de l'enfant : ___<br>(+)	conf_nbpers nbpers2.<br>conf_famille coch.<br>conf_conjoint coch.<br>conf_enfant coch.<br>enfant_age 2.

☐ Vos parents<br>☐ Mes frère(s)/ soeur(s)<br>☐ Autre(s) membre(s) de la famille élargie<br><br>☐ Des amis ou colocataires	conf_pere coch.<br>conf_frere coch.<br>conf_autrefam coch.<br><br>conf_amis coch.
