# Supplementary material for: Effects of alcohol misuse on the evolution of anxiety during the COVID-19 pandemic in France: results from CONFINS cohort
Source: BMJ Open. 2026 Jan 6;16(1):e105567. doi: 10.1136/bmjopen-2025-105567 (PMC12778323; doi:10.1136/bmjopen-2025-105567)
Supplement: online supplemental file 4 [file bmjopen-16-1-s004.pdf]

Cohorte confins

Questionnaire de suivi mensuel

v2.6 30/09/2021

Etude CONFINS

Date	Version	Commentaire
14/04/2020	v1.0	Questionnaire inclusion Population Générale + étudiants (en 2 parties)<br><br>retours utilisateurs sur la partie 1
15/04/2020	v1.1	Annotation questionnaire hebdo
05/05/2020	v1.2	Ajout question université
15/05/2020	V2.0	Questionnaire inclusion post confinement
22/05/2020	V2.1	Questionnaire à destination des étudiants et personnels de santé
15/06/2020	v2.2	Questionnaire déconfinement
28/08/2020	v2.3	Questionnaire déconfinement 2
28/09/2020	v2.4	Ajout partie perception santé dans questionnaire déconfinement 2
16/11/2020	v2.5	Mise à jour questionnaire inclusion reconfinement<br><br>mise à jour questionnaire de suivi reconfinement
30/09/2021	V2.6	MAJ Questionnaire de suivi mensuel

Questionnaire SUIVI MENSUEL

Date de remplissage du questionnaire : |__|__|/ |__|__|/ 2020
Jour Mois

(Date système remplie de manière automatique)

Vos informations

1) Pour les étudiants à l'inclusion :

- **Êtes-vous toujours étudiant.e ?**

☐ Oui ☐ Non

- **#si non#**

- **Quel est votre diplôme le plus élevé ?**

☐ Aucun

☐ BEPC (Brevet des collèges), BEP, CAP, BP (Brevet professionnel)

☐ Baccalauréat (général, technologique ou professionnel)

☐ Bac+2 (DEUG, BTS, DUT ou équivalent)

☐ Bac+3 à Bac+4 (Licence, Master 1, DEI ou équivalent)

☐ Bac+5 ou plus (Master 2, école d'ingénieur, doctorat ou équivalent)

- **Quelle est votre profession ?**

☐ Agriculteur.trice (ex. éleveur.se, viticulteur.trice)

☐ Artisan, commerçant.e, chef.fe d'entreprise (ex. électricien.ne à son compte, gérant.e)

☐ Profession intermédiaire (ex. professeur.e des écoles, technicien.ne, infirmièr.e)

☐ Cadre, ingénieur.e, profession libérale (ex. médecin, journaliste)

☐ Employé.e administratif d'entreprise ou de la fonction publique (ex. secrétaire, hôtesse de l'air/stewart, policier.e, aide-soignant.e)

☐ Employé.e de commerce (ex. caissièr.e, vendeur.se en magasin, pompiste)

☐ Personnel des services directs aux particuliers (ex. serveur.se, assistant.e maternelle)

☐ Ouvrier.e (ex. opérateur.e, chauffeur.e, peintre en bâtiment, magasinier.e)

☐ Vous n'avez pas de profession

# Si profession cochée (autre que « pas de profession ») #

- **Votre profession est-elle en rapport avec le domaine de la santé ?**

  ☐ Oui ☐ Non

# Si oui, #

- **Etes-vous soignant.e au contact de malades ?**

☐ Oui ☐ Non

- **Quel est habituellement le montant des revenus mensuels de votre ménage (c'est-à-dire vous-même et votre conjoint.e si vous habitez avec quelqu'un) en incluant toutes les sources de revenus : salaires + allocations + pensions etc. ?**

  ☐ 500 euros ou moins

  ☐ De 501 à 1700 euros

  ☐ De 1701 à 2500 euros

  ☐ De 2501 à 4000 euros

  ☐ De 4001 à 7000 euros

  ☐ Plus de 7000 euros

  ☐ Ne souhaite pas répondre

2) Pour les non étudiants à l'inclusion :

- **Souhaitez-vous indiquer une modification de votre statut professionnel depuis votre dernier questionnaire ?**

  ☐ Oui ☐ Non

  - **#si oui#**

  - **Quelle est votre profession ?**

    ☐ Agriculteur.trice (ex. éleveur.se, viticulteur.trice)

    ☐ Artisan, commerçant.e, chef.fe d'entreprise (ex. électricien.ne à son compte, gérant.e)

    ☐ Profession intermédiaire (ex. professeur.e des écoles, technicien.ne, infirmièr.e)

    ☐ Cadre, ingénieur.e, profession libérale (ex. médecin, journaliste)

    ☐ Employé.e administratif d'entreprise ou de la fonction publique (ex. secrétaire, hôtesse de l'air/stewart, policier.e, aide-soignant.e)

    ☐ Employé.e de commerce (ex. caissièr.e, vendeur.se en magasin, pompiste)

    ☐ Personnel des services directs aux particuliers (ex. serveur.se, assistant.e maternelle)

    ☐ Ouvrier.e (ex. opérateur.e, chauffeur.e, peintre en bâtiment, magasinier.e)

☐ Vous n’avez pas de profession

# Si profession cochée (autre que « pas de profession ») #

- **Votre profession est-elle en rapport avec le domaine de la santé ?**

☐ Oui ☐ Non

# Si oui, #

- **Etes-vous soignant.e au contact de malades ?**

☐ Oui ☐ Non

- **Quel est habituellement le montant des revenus mensuels de votre ménage (c'est-à-dire vous-même et votre conjoint.e si vous habitez avec quelqu’un) en incluant toutes les sources de revenus : salaires + allocations + pensions etc. ?**

☐ 500 euros ou moins

☐ De 501 à 1700 euros

☐ De 1701 à 2500 euros

☐ De 2501 à 4000 euros

☐ De 4001 à 7000 euros

☐ Plus de 7000 euros

☐ Ne souhaite pas répondre

3) Pour tous :

- **Souhaitez-vous indiquer une modification de votre situation familiale depuis votre dernier questionnaire ?**

☐ Oui ☐ Non

- **#si oui#**

- **Votre situation de famille**

☐ Célibataire

☐ En couple (depuis au moins 3 mois) sans être marié.e ni pacsé.e

☐ Marié.e, Pacsé.e

☐ Autre (divorcé.e, veuf.ve)

- **Avez-vous eu des enfants depuis le remplissage de votre dernier questionnaire ?**

☐ Oui ☐ Non

Votre suivi mensuel

M1. Depuis le dernier questionnaire, pensez-vous avoir contracté la COVID-19 ?

- ❒ Oui, j'ai été testé.e positif/ve
- ❒ Oui, cela a été évoqué par un médecin mais je n'ai pas été testé.e ou j'ai été testé.e négatif/ve
- ❒ C'est possible, je présente des symptômes (fièvre, toux, courbatures, fatigue intense, diarrhées, douleur thoracique, perte de l'odorat, gêne respiratoire)
- ❒ C'est peu probable, je ne me sens pas malade
- ❒ Je suis certain.e de ne pas être atteint.e
- ❒ Je ne sais pas

# Si Oui, j'ai été testé(e) positif, ajouter M2 #

M2. Pensez-vous avoir contracté la COVID-19 au cours de votre exercice professionnel ?

- ❒ Oui, j'en suis certain.e
- ❒ Oui, c'est possible
- ❒ Non, je ne pense pas
- ❒ Je ne sais pas

M3. <u>Au cours des 7 derniers jours</u>, avez-vous pratiqué une activité physique ?

- ❒ Non
- ❒ Oui, mais pas tous les jours
- ❒ Oui, tous les jours

M4. <u>Au cours des 7 derniers jours</u>, sur une échelle de 0 à 10 (0=pas du tout, 10=totalement), à quel point vous sentez-vous seul.e ?

0 1 2 3 4 5 6 7 8 9 10

M5. À quel point êtes-vous inquiet.ète ou stressé.e en ce moment sur une échelle de 0 à 10 ?

Donnez une note entre 0 (pas de stress) et 10 (très stressé(e)).

0 1 2 3 4 5 6 7 8 9 10

M6. <u>Au cours des 7 derniers jours</u>, comment avez-vous dormi ?

- ❒ Bien
- ❒ Plutôt bien
- ❒ Ni bien ni mal
- ❒ Plutôt mal
- ❒ Mal

M7. <u>Au cours des 7 derniers jours,</u> avez-vous eu des difficultés d'endormissement et/ou de maintien de votre sommeil (réveils nocturnes) ?

- ❒ Jamais ou moins d'1 fois par mois
- ❒ Moins d'1 fois par semaine
- ❒ 1 à 2 jours par semaine
- ❒ 3 à 5 jours par semaine
- ❒ Tous les jours ou presque

Nous vous proposons de répondre maintenant à plusieurs questions sur votre santé psychique. Si vous avez besoin de soutien psychologique, ou si certaines questions vous ont déstabilisé.e ou perturbé.e, n'hésitez pas à faire appel à l'un des nombreux dispositifs d'aide à distance dont vous trouverez les coordonnées dans l'onglet « Contact ».

<u>Au cours des 2 dernières semaines</u>, à quelle fréquence avez-vous été dérangé.e par les problèmes suivants ?

		Presque jamais	Plusieurs jours durant ces 2 dernières semaines	Plus de la moitié du temps	Presque tous les jours
**M8.**	Peu d'intérêt ou de plaisir à faire les choses	❑	❑	❑	❑
**M9.**	Se sentir triste, déprimé.e ou désespéré.e	❑	❑	❑	❑
**M1(**	Difficultés à s'endormir ou à rester endormi.e, ou trop dormir	❑	❑	❑	❑
**M1:**	Se sentir fatigué.e ou avoir peu d'énergie	❑	❑	❑	❑
**M1:**	Peu d'appétit ou trop manger	❑	❑	❑	❑
**M1:**	Mauvaise perception de vous-même - ou vous pensez que vous êtes un.e perdant.e ou que vous n'avez pas satisfait vos propres attentes ou celles de votre famille	❑	❑	❑	❑
**M1‹**	Difficultés à se concentrer sur des choses telles que lire le journal ou regarder la télévision	❑	❑	❑	❑
**M1!**	Vous bougez ou parlez si lentement que les autres personnes ont pu le remarquer. Ou au contraire - vous êtes si agité.e que	❑	❑	❑	❑

	vous bougez beaucoup plus que d'habitude				
M1[illegible]	Vous avez pensé que vous seriez mieux mort.e ou vous avez pensé à vous blesser d'une façon ou d'une autre	❑	❑	❑	❑

<u>Au cours des 2 dernières semaines</u>, à quelle fréquence avez-vous été gêné.e par les problèmes suivants ?

		**Presque jamais**	**Plusieurs jours au cours de ces 2 dernières semaines**	**Plus de la moitié du temps**	**Presque tous les jours**
**M17.**	Un sentiment de nervosité, d'anxiété ou de tension	❑	❑	❑	❑
**M18.**	Une incapacité à arrêter de s'inquiéter ou à contrôler ses inquiétudes	❑	❑	❑	❑
**M19.**	Une inquiétude excessive à propos de différentes choses	❑	❑	❑	❑
**M20.**	Des difficultés à me détendre	❑	❑	❑	❑
**M21.**	Une agitation telle qu'il m'est difficile de tenir en place	❑	❑	❑	❑
**M22.**	Une tendance à être facilement contrarié.e ou irritable	❑	❑	❑	❑
**M23.**	Un sentiment de peur comme si quelque chose de terrible risquait de se produire	❑	❑	❑	❑

M24. **<u>Au cours des 2 dernières semaines</u>, vous est-il arrivé de penser à vous suicider (d'avoir des idées suicidaires) ?**

- ❒ Non, jamais
- ❒ Oui, quelquefois
- ❒ Oui, à de multiples reprises

# Si oui, quelquefois ou Oui, à de multiples reprises, → M25 ET M26 #

M25. **Avez-vous pensé à la manière dont vous vous y prendriez ?**

- ❒ Non
- ❒ Oui, mais pas dans le détail
- ❒ Oui, j'ai réfléchi à un scénario précis

M26. **Vous est-il arrivé de penser à vous suicider (d'avoir des idées suicidaires) au cours des 12 derniers mois ?**

- ❒ Non, jamais
- ❒ Oui, quelquefois
- ❒ Oui, à de multiples reprises

M27. **Actuellement, quelle note donneriez-vous à votre qualité de vie ?**

(0 pour la pire qualité de vie possible et 10 pour la meilleure qualité de vie possible)

0 1 2 3 4 5 6 7 8 9 10

Si vous souhaitez bénéficier d'un bilan personnalisé de votre santé psychique (stress, anxiété, dépression), basé sur vos réponses à ce questionnaire, rendez-vous sur l'appli CONFINS (lien téléchargeable). Vous y retrouverez vos résultats, leur interprétation et leur évolution graphique chaque mois. Vous aurez également accès à des informations en lien avec la santé mentale. A très bientôt !
