# Supplementary material for: Effects of alcohol misuse on the evolution of anxiety during the COVID-19 pandemic in France: results from CONFINS cohort
Source: BMJ Open. 2026 Jan 6;16(1):e105567. doi: 10.1136/bmjopen-2025-105567 (PMC12778323; doi:10.1136/bmjopen-2025-105567)
Supplement: Supplementary file 5 [file bmjopen-2025-105567.reviewer_comments.pdf]

PEER REVIEW HISTORY

BMJ Open publishes all reviews undertaken for accepted manuscripts. Reviewers are asked to complete a checklist review form and are provided with free text boxes to elaborate on their assessment. These free text comments are reproduced below.

ARTICLE DETAILS

Title (Provisional)

Effects of alcohol misuse on the evolution of anxiety during the COVID-19 pandemic in France: results from CONFINS cohort

Authors

Galesne, Charline; Arsandaux, Julie; MACALLI, Mélissa; TEXIER, Nathalie; SCHÜCK, Stéphane; Tzourio, Christophe; Kinouani, Shérazade

---

VERSION 1 - REVIEW

**Reviewer**	**1**
**Name**	**Blithikioti, Chrysanthi**
**Affiliation**	**Universita degli Studi di Padova Scuola di Psicologia**
**Date**	**03-Sep-2025**
**COI**	**None**

In this manuscript, the authors aim to describe the evolution of anxiety during the COVID-19 pandemic and its relationship with alcohol misuse, using a prospective longitudinal design. There are a few points that need to be addressed before publication:

1) The rationale of the study is not entirely clear. For instance, the authors mention in the abstract and methods that the main outcome is alcohol misuse, however, they do not report how alcohol misuse levels evolved during the pandemic. Additionally, in the methods, they describe alcohol misuse as both the main outcome and the main independent variable, which is not conceptually coherent.

2) Several confounders were included in the analysis, but it is not justified how these confounders were chosen.

3) Was there a relationship between marital/relationship status and perceived loneliness?

4) The authors performed stratified analyses by gender and perceived loneliness, although the interaction terms were not statistically significant. Was this decision pre-specified?

5) Did the authors control for multiple comparisons in the post-hoc tests?

6) There is a typo on page 5, line 11 "and changes in anxiety from in France"

7) The introduction would benefit from narrowing its scope to the study's objectives. For instance, the AUDIT test is mentioned in the first paragraph of the introduction, while it would be more appropriate in the methods section.

**Reviewer**	**2**
**Name**	**Slobodin, Ortal**
**Affiliation**	**Ben-Gurion University of the Negev**
**Date**	**26-Oct-2025**
**COI**	**None**

Thank you for the opportunity to review the paper, which describes the evolution of anxiety during the COVID-19 pandemic in France and its relationship to pre-existing alcohol use. The paper is well-written and is based on a prospective design, with repeated measures over a period of almost 2 years after the first lockdown. I have several minor issues that the authors may wish to address.

1. In the abstract, it is stated that the objective of the study was " to describe the evolution of anxiety during the COVID-19 pandemic in France, according to a pre-existing alcohol misuse". The formulation of this sentence sounds a bit strange, as if the levels of anxiety are measured by the levels of pre-existing alcohol misuse. I think it would be better to clarify the relationship between these two variables.

2. In the method, please explain more specifically how participants were recruited through social networks (professional or academic) and press communications. For instance, which networks were used? This point should also be discussed more systematically in the limitation section by addressing the shortcomings involved in snowball nonprobability convenience sampling. Please see;

Barnes, L. A., Barclay, L., McCaffery, K. J., Rolfe, M. I., & Aslani,

P. (2020). Using Facebook to recruit to a national online survey

investigating complementary medicine product use in

pregnancy and lactation: A case study of method. Research in

Social & Administrative Pharmacy, 17, 864–874. https://doi.

org/10.1016/j.sapharm.2020.07.011

Leighton, K., Kardong-Edgren, S., Schneidereith, T. A., & Foisy-

Doll, C. (2021). Using social media and snowball sampling

as an alternative recruitment strategy for research. Clinical

Simulation in Nursing, 55, 37–42. https://doi.org/10.1016/J.

ECNS.2021.03.006

3. Is there any information about the race/ethnicity/ immigration backgrounds of participants?

4. In the discussion, the finding that women have been identified as being at a greater risk of high anxiety levels during the COVID-19 pandemic is not sufficiently discussed. This is an interesting finding that deserves more attention. For example, why are women at higher risk? is it related to gender role division? Are women are vulnerable to other mental health problems during COVID? Some useful papers are :

Sriharan A, Ratnapalan S, Tricco AC, Lupea D, Ayala AP, Pang H and Lee DD (2020) Occupational Stress, Burnout, and Depression in Women in Healthcare During COVID-19 Pandemic: Rapid Scoping Review. Front. Glob. Womens Health 1:596690. doi: 10.3389/fgwh.2020.596690

Pariente, G., Wissotzky Broder, O., Sheiner, E. et al. Risk for probable post-partum depression among women during the COVID-19 pandemic. Arch Womens Ment Health 23, 767–773 (2020). https://doi.org/10.1007/s00737-020-01075-3

Download citation

Hou, F., Bi, F., Jiao, R. et al. Gender differences of depression and anxiety among social media users during the COVID-19 outbreak in China:a cross-sectional study. BMC Public Health 20, 1648 (2020). https://doi.org/10.1186/s12889-020-09738-7

Tsukamoto R, Kataoka Y, Mino K, Ishibashi N, Shibata M, Matsuo H and Fujiwara H (2021) Gender Differences in Anxiety Among COVID-19 Inpatients Under Isolation: A Questionnaire Survey During the First and Second Waves of the COVID-19 Pandemic in Japan. Front. Public Health 9:708965. doi: 10.3389/fpubh.2021.708965

5. In the limitations, it is stated that the women and young people were overrepresented in the sample. Please address how this fact might affect your results (e.g., given that women suffer from lower levels of alcohol misuse).

VERSION 1 - AUTHOR RESPONSE

Reviewer: 1

Dr. Chrysanthi Blithikioti, Universita degli Studi di Padova Scuola di Psicologia

Comments to the Author:

In this manuscript, the authors aim to describe the evolution of anxiety during the COVID-19 pandemic and its relationship with alcohol misuse, using a prospective longitudinal design. There are a few points that need to be addressed before publication:

1) The rationale of the study is not entirely clear. For instance, the authors mention in the abstract

and methods that the main outcome is alcohol misuse, however, they do not report how alcohol misuse levels evolved during the pandemic. Additionally, in the methods, they describe alcohol misuse as both the main outcome and the main independent variable, which is not conceptually coherent.

We thank the reviewer for pointing out this inconsistency. This was indeed a wording error in the manuscript. In our study, alcohol misuse is the main exposure, and anxiety (measured by GAD-7) is the main outcome. We have corrected the text accordingly in the Abstract and Methods sections to ensure conceptual consistency and avoid confusion.

2) Several confounders were included in the analysis, but it is not justified how these confounders were chosen.

We thank the reviewer for this comment. Potential confounders were selected *a priori* based on existing literature and biological/psychosocial plausibility, with variables known or suspected to be associated both with alcohol misuse and with mental health outcomes. We clarified this rationale in the Methods section as follows: *"We identified potential confounders at baseline that could be associated with the presence of alcohol misuse and mental health outcome, based on prior literature and theoretical considerations regarding factors likely to influence both exposure and outcome."*

3) Was there a relationship between marital/relationship status and perceived loneliness?

We thank the reviewer for this question. As expected and consistent with previous literature, perceived loneliness was significantly associated with marital/relationship status, with higher levels of perceived loneliness among participants who were single, divorced, or widowed compared with those in a relationship. However, perceived loneliness reflects a subjective feeling of social isolation that is not entirely captured by marital status. For this reason, we considered perceived loneliness as an effet modifier in our analyses.

4) The authors performed stratified analyses by gender and perceived loneliness, although the interaction terms were not statistically significant. Was this decision pre-specified?

We thank the reviewer for this comment on an important methodological choice. Yes, the decision to perform stratified analyses by gender and perceived loneliness was pre-specified. These two variables were identified as potential effect modifiers based on previous literature showing that both gender and loneliness could influence the relationship between alcohol use and mental health outcomes. Although the statistical tests for interaction were not significant, we considered that presenting stratified results could provide valuable information on potential differences in the strength or direction of associations across these subgroups. We have clarified this rationale in the Methods section as follows: *"We tested interactions between gender or perceived loneliness and alcohol misuse, which were pre-specified based on prior literature suggesting possible differences."*

5) Did the authors control for multiple comparisons in the post-hoc tests?

We thank the reviewer for this comment. No correction for multiple comparisons was applied, as both the stratified analyses (by gender and perceived loneliness) and the sensitivity analysis were pre-specified based on prior literature and formed part of our initial analysis plan. These analyses were therefore not considered post-hoc exploratory tests requiring adjustment for multiple testing.

6) There is a typo on page 5, line 11 "and changes in anxiety from in France"

The typo has been corrected in the revised version of the manuscript.

7) The introduction would benefit from narrowing its scope to the study's objectives. For instance, the AUDIT test is mentioned in the first paragraph of the introduction, while it would be more appropriate in the methods section.

We thank the reviewer for this helpful suggestion. We agree that the introduction can be more focused on the study rationale and objectives. Accordingly, we have removed the detailed description of the AUDIT-C from the introduction and relocated this information to the Methods section, where the measurement of alcohol misuse is described in detail.

Reviewer: 2
Dr. Ortal Slobodin, Ben-Gurion University of the Negev

Comments to the Author:

Thank you for the opportunity to review the paper, which describes the evolution of anxiety during the COVID-19 pandemic in France and its relationship to pre-existing alcohol use. The paper is well-written and is based on a prospective design, with repeated measures over a period of almost 2 years after the first lockdown. I have several minor issues that the authors may wish to address.

1. In the abstract, it is stated that the objective of the study was " to describe the evolution of anxiety during the COVID-19 pandemic in France, according to a pre-existing alcohol misuse". The formulation of this sentence sounds a bit strange, as if the levels of anxiety are measured by the levels of pre-existing alcohol misuse. I think it would be better to clarify the relationship between these two variables.

We thank the reviewer for this helpful suggestion. We agree that the original phrasing could be misleading. We have rephrased the objective to better reflect that pre-existing alcohol misuse was considered as the exposure and anxiety as the outcome. The revised sentence now reads: *"Objectives: To describe the evolution of anxiety during the COVID-19 pandemic in France and to assess whether it differed according to pre-existing alcohol misuse."*

2. In the method, please explain more specifically how participants were recruited through social networks (professional or academic) and press communications. For instance, which networks were used? This point should also be discussed more systematically in the limitation section by addressing the shortcomings involved in snowball nonprobability convenience sampling. Please see;

Barnes, L. A., Barclay, L., McCaffery, K. J., Rolfe, M. I., & Aslani, P. (2020). Using Facebook to recruit to a national online survey investigating complementary medicine product use in pregnancy and lactation: A case study of method. Research in Social & Administrative Pharmacy, 17, 864–874. https://doi.org/10.1016/j.sapharm.2020.07.011

Leighton, K., Kardong-Edgren, S., Schneidereith, T. A., & Foisy-Doll, C. (2021). Using social media and snowball

sampling as an alternative recruitment strategy for research. Clinical Simulation in Nursing, 55, 37–42. https://doi.org/10.1016/J.ECNS.2021.03.006

We thank the reviewer for this helpful comment. We have added further details on the recruitment process in the Methods section to clarify how participants were reached and encouraged to participate. The revised paragraph now specifies that participants were recruited through online and media channels, including social media (LinkedIn, Twitter, Facebook), as well as press releases and media interviews with the principal investigators. We also mention that newsletters, emails, and text messages were used to remind participants to complete follow-up questionnaires.
In addition, we have revised the first paragraph of the *Limitations* section to more explicitly discuss the shortcomings associated with convenience and snowball nonprobability sampling. We now acknowledge that this recruitment strategy may have introduced self-selection bias and limited the generalisability of our findings, and we have added the suggested references to support this discussion.

3. Is there any information about the race/ethnicity/ immigration backgrounds of participants?

We thank the reviewer for this question. No data on race, ethnicity, or immigration background were collected in the CONFINS cohort. In France, race and ethnicity are considered sensitive personal data under the General Data Protection Regulation (GDPR) and are subject to strict regulations by the French Data Protection Authority (CNIL). Collecting such data requires a specific authorization from the CNIL, in addition to standard ethical approval. Given the urgent context of launching the CONFINS study at the onset of the COVID-19 pandemic, it was not feasible to obtain this additional authorization without delaying the start of data collection. For this reason, no information on participants' ethnic or migration background was included in the study.

4. In the discussion, the finding that women have been identified as being at a greater risk of high anxiety levels during the COVID-19 pandemic is not sufficiently discussed. This is an interesting finding that deserves more attention. For example, why are women at higher risk? is it related to gender role division? Are women are vulnerable to other mental health problems during COVID? Some useful papers are:

Sriharan A, Ratnapalan S, Tricco AC, Lupea D, Ayala AP, Pang H and Lee DD (2020) Occupational Stress, Burnout, and Depression in Women in Healthcare During COVID-19 Pandemic: Rapid Scoping Review. Front. Glob. Womens Health 1:596690. doi: 10.3389/fgwh.2020.596690

Pariente, G., Wissotzky Broder, O., Sheiner, E. et al. Risk for probable post-partum depression among women during the COVID-19 pandemic. Arch Womens Ment Health 23, 767–773 (2020). https://doi.org/10.1007/s00737-020-01075-3

Hou, F., Bi, F., Jiao, R. et al. Gender differences of depression and anxiety among social media users during the COVID-19 outbreak in China:a cross-sectional study. BMC Public Health 20, 1648 (2020). https://doi.org/10.1186/s12889-020-09738-7

Tsukamoto R, Kataoka Y, Mino K, Ishibashi N, Shibata M, Matsuo H and Fujiwara H (2021) Gender Differences in Anxiety Among COVID-19 Inpatients Under Isolation: A Questionnaire Survey During the First and Second Waves of the COVID-19 Pandemic in Japan. Front. Public Health 9:708965. doi: 10.3389/fpubh.2021.708965

We thank the reviewer for this insightful comment and for suggesting relevant references. We agree that this finding deserves further discussion. We have therefore expanded the section of the Discussion

addressing gender differences in anxiety during the COVID-19 pandemic. The revised paragraph now elaborates on potential biological, psychological, and social mechanisms underlying women's greater vulnerability to anxiety, including gender role division, increased caregiving and occupational burden during lockdowns, and the overrepresentation of women in healthcare professions. We have also incorporated the suggested citations to strengthen this discussion.

5. In the limitations, it is stated that the women and young people were overrepresented in the sample. Please address how this fact might affect your results (e.g., given that women suffer from lower levels of alcohol misuse).

We thank the reviewer for this relevant comment. We have now expanded the Limitations section to discuss how the overrepresentation of women and younger participants may have influenced our results. Because women generally report lower levels of alcohol misuse compared to men, this sampling bias could have led to an underestimation of the overall prevalence of alcohol misuse in our study population. However, it is unlikely to have affected the associations observed between alcohol misuse and anxiety, as our analyses were stratified.

VERSION 2 - REVIEW

**Reviewer**	**1**
**Name**	**Blithikioti, Chrysanthi**
**Affiliation**	**Universita degli Studi di Padova Scuola di Psicologia**
**Date**	**15-Dec-2025**
**COI**	

The authors have addressed all my comments adequately
